# Complex Ancestries of Lager-Brewing Hybrids Were Shaped by Standing Variation in the Wild Yeast *Saccharomyces eubayanus*

**DOI:** 10.1371/journal.pgen.1006155

**Published:** 2016-07-06

**Authors:** David Peris, Quinn K. Langdon, Ryan V. Moriarty, Kayla Sylvester, Martin Bontrager, Guillaume Charron, Jean-Baptiste Leducq, Christian R. Landry, Diego Libkind, Chris Todd Hittinger

**Affiliations:** 1 Laboratory of Genetics, Genome Center of Wisconsin, Wisconsin Energy Institute, J. F. Crow Institute for the Study of Evolution, University of Wisconsin-Madison, Madison, Wisconsin, United States of America; 2 DOE Great Lakes Bioenergy Research Center, University of Wisconsin-Madison, Madison, Wisconsin, United States of America; 3 Institut de Biologie Intégrative et des Systèmes (IBIS), Département de Biologie, PROTEO, Pavillon Charles-Eugène-Marchand, Université Laval, Québec City, Québec, Canada; 4 Laboratorio de Microbiología Aplicada, Biotecnología y Bioinformática, Instituto Andino Patagonico de Tecnologías Biológicas y Geoambientales, IPATEC (CONICET-UNComahue), Centro Regional Universitario Bariloche, Bariloche, Río Negro, Argentina; Washington University School of Medicine, UNITED STATES

## Abstract

Lager-style beers constitute the vast majority of the beer market, and yet, the genetic origin of the yeast strains that brew them has been shrouded in mystery and controversy. Unlike ale-style beers, which are generally brewed with *Saccharomyces cerevisiae*, lagers are brewed at colder temperatures with allopolyploid hybrids of *Saccharomyces eubayanus x S*. *cerevisiae*. Since the discovery of *S*. *eubayanus* in 2011, additional strains have been isolated from South America, North America, Australasia, and Asia, but only interspecies hybrids have been isolated in Europe. Here, using genome sequence data, we examine the relationships of these wild *S*. *eubayanus* strains to each other and to domesticated lager strains. Our results support the existence of a relatively low-diversity (π = 0.00197) lineage of *S*. *eubayanus* whose distribution stretches across the Holarctic ecozone and includes wild isolates from Tibet, new wild isolates from North America, and the *S*. *eubayanus* parents of lager yeasts. This Holarctic lineage is closely related to a population with higher diversity (π = 0.00275) that has been found primarily in South America but includes some widely distributed isolates. A second diverse South American population (π = 0.00354) and two early-diverging Asian subspecies are more distantly related. We further show that no single wild strain from the Holarctic lineage is the sole closest relative of lager yeasts. Instead, different parts of the genome portray different phylogenetic signals and ancestry, likely due to outcrossing and incomplete lineage sorting. Indeed, standing genetic variation within this wild Holarctic lineage of *S*. *eubayanus* is responsible for genetic variation still segregating among modern lager-brewing hybrids. We conclude that the relationships among wild strains of *S*. *eubayanus* and their domesticated hybrids reflect complex biogeographical and genetic processes.

## Author Summary

Yeasts are key industrial microbes, most notably *Saccharomyces cerevisiae*, which is used to make a variety of products, including bread, wine, and ale-style beers. However, lager-style beers are brewed with interspecies hybrids of *S*. *cerevisiae x Saccharomyces eubayanus*. After its discovery in South America in 2011, rare strains of *S*. *eubayanus* have also been isolated outside of South America. Here we compare the genome sequences of several new and recent isolates of *S*. *eubayanus* from South America, North America, Australasia, and Asia to unravel the relationships of these wild isolates and their domesticated European hybrids. Two South American populations have the highest genetic diversity. One of these populations is closely related to a relatively low-diversity lineage that is spread across the Northern Hemisphere and includes the *S*. *eubayanus* parents of lager yeasts. Interestingly, we find that none of the wild isolates of *S*. *eubayanus* is the sole closest relative of lager-brewing hybrids. Instead, we show that standing variation among wild *S*. *eubayanus* strains contributed to the genetic makeup of lager yeasts. Our findings highlight the complex ancestries of lager yeasts and the importance of broader sampling of wild yeasts to illuminate our understanding of the sources of genetic variation among industrial hybrids.

## Introduction

Humans changed from living in hunter-gatherer societies to agricultural societies in part through the domestication of animals and plants [1,2]. At the same time, humans began unwittingly domesticating microorganisms for the production of fermented beverages and foods, but the underlying source populations and genetic processes for microbial domestication are not well understood [3]. Beer is the most common fermented beverage in the world and can be classified as ale or lager, depending on the fermentation conditions and yeasts used. Ale-style beers are mainly produced by strains of *S*. *cerevisiae* [4]. In contrast, 94% of the beer market is dominated by lager-style beers, which are fermented at colder temperatures by allopolyploid hybrids of *S*. *cerevisiae x S*. *eubayanus* (syn. *S*. *pastorianus* syn. *S*. *carlsbergensis*) [5].

Two hybrid lineages of lager-brewing yeasts have been described based on genome content and phenotypic traits [6–9], leading to extensive debate about their origins. The two simplest models proposed to explain the origins of the Saaz and Frohberg lineages are through a single shared hybridization event [9–11] or through two or more independent hybridization events [6,12–15]. More complex models involving backcrossing have also been discussed by several authors [9–11,14,15]. All known modern lager strains are aneuploid. Genetic contributions from *S*. *eubayanus* have been argued to confer enhanced cold-tolerance, while genetic contributions from *S*. *cerevisiae* may confer other adaptions to the brewing environment, such as maltotriose fermentation [16–19].

Although the *S*. *cerevisiae* parent of lager yeasts seems to be closely related to modern ale strains [6,13,15], identifying close relatives of the *S*. *eubayanus* parent has proven more challenging. Since the discovery of the species in 2011 in Patagonia, South America [5], rare strains of *S*. *eubayanus* have been isolated in North America [20], Asia [21], and New Zealand [22]. Other than interspecies hybrids [5,23], no European isolates of *S*. *eubayanus* have been reported. Genome sequence comparisons have shown the Patagonian type strain to be 99.56% identical to the *S*. *eubayanus* subgenome of a lager-brewing hybrid [5], while a Tibetan isolate was shown to be 99.82% identical [21].

Previous population and phylogenetic studies of *S*. *eubayanus* suggest that it may contain up to five known phylogenetically distinct clades. Two distinct and highly diverse populations have been described in South America (Patagonia A and Patagonia B) where they have been commonly associated with *Nothofagus* [20], as well as *Araucaria araucana* [24]. Recently, an isolate from New Zealand was shown to belong to the Patagonia B clade by multi-locus phylogenetic analysis [22]. Previously isolated North American strains were shown to be the result of recent admixture between the two Patagonian populations [20]. Three lineages have been isolated in Asia, mostly in association with *Quercus*, including the Tibetan lineage and two early-diverging lineages that could be regarded as distinct subspecies (Sichuan and West China) [21]. Analyses of population differentiation and genetic diversity have not been performed on the latter three lineages, and all five lineages have not been thoroughly analyzed together in the same phylogenetic study.

To improve our understanding of the genetic diversity and phylogeography of *S*. *eubayanus* and its domesticated European hybrids, we have integrated existing multi-locus datasets and added several new isolates from North America (North Carolina, Washington, and New Brunswick). To extend these analyses, we have also performed whole genome sequencing (WGS) on available isolates. These results support the existence of a relatively low-diversity Holarctic lineage, which includes wild isolates from Tibet and North Carolina, as well as the hypothetical ancestor of the European interspecies hybrids. Depending on the region of the genome examined, this Holarctic lineage is embedded within or sister to one of the Patagonian populations of *S*. *eubayanus*. Genomic analyses further show that none of the known wild *S*. *eubayanus* strains is the sole closest relative to the lager-brewing hybrids. Instead, we infer that lager yeasts drew from alleles that were segregating among a Holarctic lineage of *S*. *eubayanus*.

## Results

### Broad *Saccharomyces eubayanus* geographic and ecological distribution

**Fig 1:**
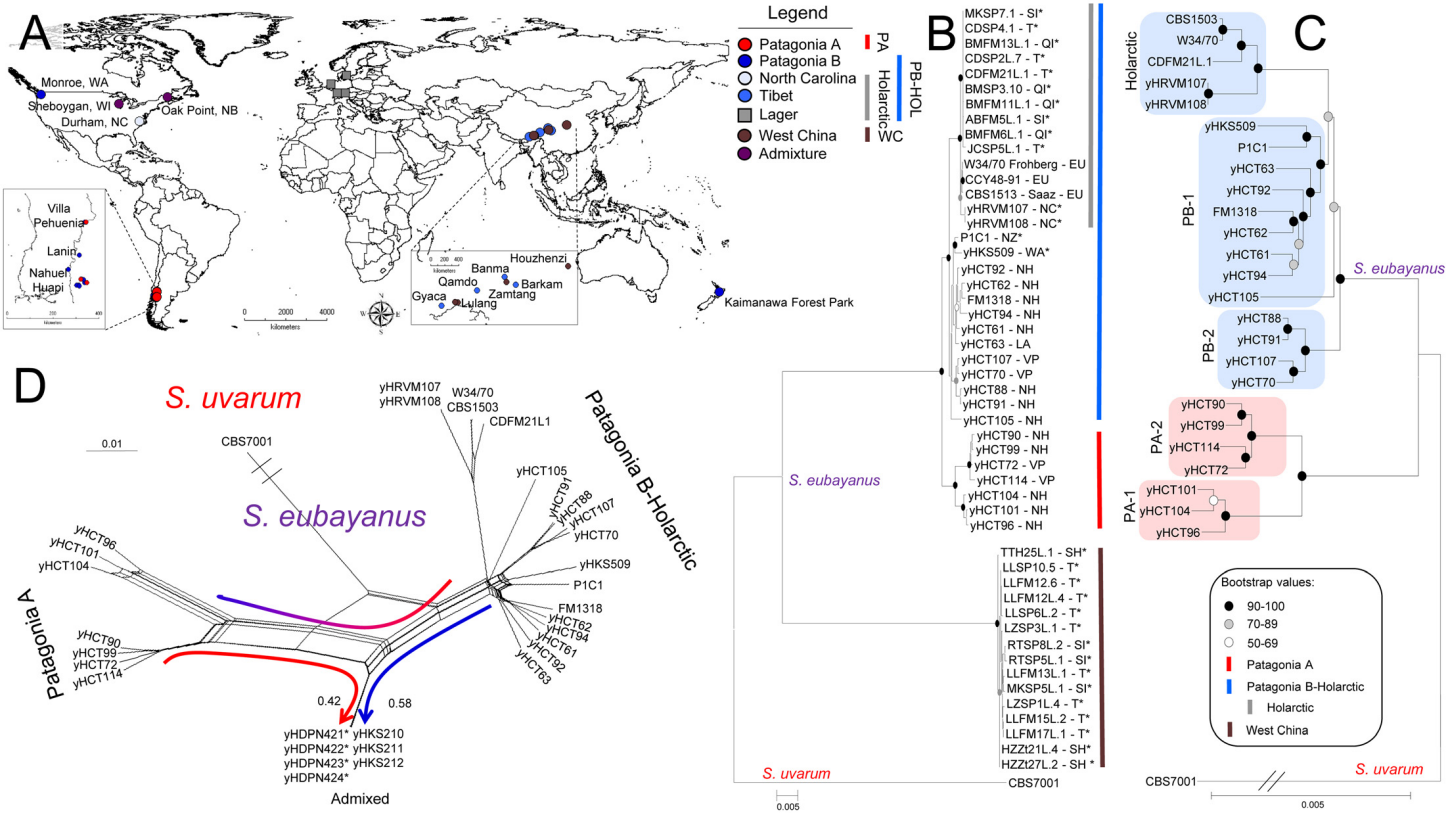
*S*. *eubayanus* distribution and phylogeography. A) Geographic distribution of *S*. *eubayanus* isolates. B) Maximum-Likelihood (ML) phylogenetic tree reconstructed using the concatenated multi-locus Dataset A (S1 Text). Bar colors are defined in the legend in panel A. Asterisks highlight new isolates or strains not previously studied together [20]. EU: Europe; QI: Qinghai (China); LA: Lanin (Argentina); NC: North Carolina (USA); NH: Nahuel Huapi (Argentina); NZ: New Zealand; SH: Shaanxi (China); SI: Sichuan (China); T: Tibet (China); VP: Villa Pehuenia (Argentina); WA: Washington (USA). C) ML phylogenetic tree reconstructed using the complete genome sequence data. Phylogenetic trees were rooted using *S*. *uvarum* (CBS7001) as the outgroup. The scale bars show the number of substitutions per site. The strain FM1318 is a monosporic derivative of CRUB1568^T^ (= CBS12357^T^ = PYCC6148^T^). Bootstrap values above 50 are reported at their corresponding nodes. D) Neighbor-Net phylogenetic network reconstructed with the SNP dataset. In phylogenetic networks, incongruent data are represented by nodes subtended by multiple edges. Blue and red arrows indicate the fractional genomic contributions from PB-1 and PA-2, respectively. The scale bar represents the number of substitutions. Note that the admixed strains from Wisconsin [20] and New Brunswick (Fig 2) are only shown in panel D to avoid implying a linear bifurcating ancestry.

Our ongoing high-sugar enrichment surveys of yeast from soil, leaves, bark, mushrooms, and other natural substrates in North America isolated seven new strains of *S*. *eubayanus*: one from Washington State, USA; two from North Carolina, USA; and four from New Brunswick, Canada (Fig 1A, S1 Table). The new *S*. *eubayanus* strains were isolated from novel tree hosts, including the bark of *Cedrus* sp., the bark and soil of *Pinus taeda*, and the bark of *Quercus rubra*. North American isolates of *S*. *eubayanus* remained quite rare overall (<1% of yeast isolates), except at specific sampling sites, and were only slightly biased toward the tree order Fagales (S1 Text, S1 Fig).

### Some North American strains are closely related to lager-brewing yeasts

To determine how the new North American strains are related to South American [5,20], Asian [21], and New Zealand strains [22], we performed multi-locus phylogenetic analyses. Specifically, we partially sequenced nine nuclear coding sequences and three nuclear intergenic regions, consisting of a total of ~9.8 kbp, as well as one mitochondrial gene (500 bp). Existing multi-locus data was utilized at this stage, rather than WGS data, because the Chinese strains are not available for study.

North American strains displayed three different types of ancestry: 1) the strain from Washington was embedded within the Patagonia B clade and was more closely related to the strain from New Zealand than any other Patagonia B strain, 2) the strains from New Brunswick were identical at these loci to three previously characterized admixed strains from Wisconsin, USA [20], and 3) the strains from North Carolina were closely related to the strains from Tibet and lager beer (Fig 1B, S1 Text). This latter "Holarctic" subgroup of strains (Tibet, North Carolina, and Lager) was well supported phylogenetically and was more closely related to the Patagonia B clade than to any other population (Fig 1B). Phylogenetic supernetwork analysis and examination of the individual gene trees revealed a complex history for the strains in the Patagonian populations and their close Holarctic relatives, but it failed to unambiguously identify the closest relative of lager yeasts (S2 and S3 Figs, S1 Text).

To determine the consensus relationships among the wild populations of *S*. *eubayanus* and the domesticated lager-brewing hybrids, we compared the complete genome sequences of 33 strains, including representatives of both known lager yeast lineages (Saaz and Frohberg) and *S*. *uvarum* as the outgroup. In contrast to previously reported topologies citing a personal communication [25] and weak support in a multi-locus dataset [22], WGS data strongly agreed with our multi-locus phylogenetic tree and placed the Patagonia A population as an outgroup to a clade containing the Patagonia B population plus the strains from the Holarctic lineage (Fig 1C). Even with WGS data, it remained unclear whether the Holarctic subgroup was embedded within the Patagonia B population or was sister to it. In contrast, the New Zealand strain was closely related to the Washington strain, both falling within Patagonia B. These analyses further showed that, on average, the *S*. *eubayanus* subgenomes of both the Saaz and Frohberg lager yeast lineages were more closely related to the representative strain from Tibet than to known strains from North Carolina or Patagonia. Nonetheless, analysis of the full single nucleotide polymorphism (SNP) dataset revealed extensively conflicting phylogenetic signals, which are displayed by the presence of nodes subtended by multiple edges in a phylogenetic network (Fig 1D).

### No wild isolate is the sole closest relative of lager-brewing yeasts

**Fig 2:**
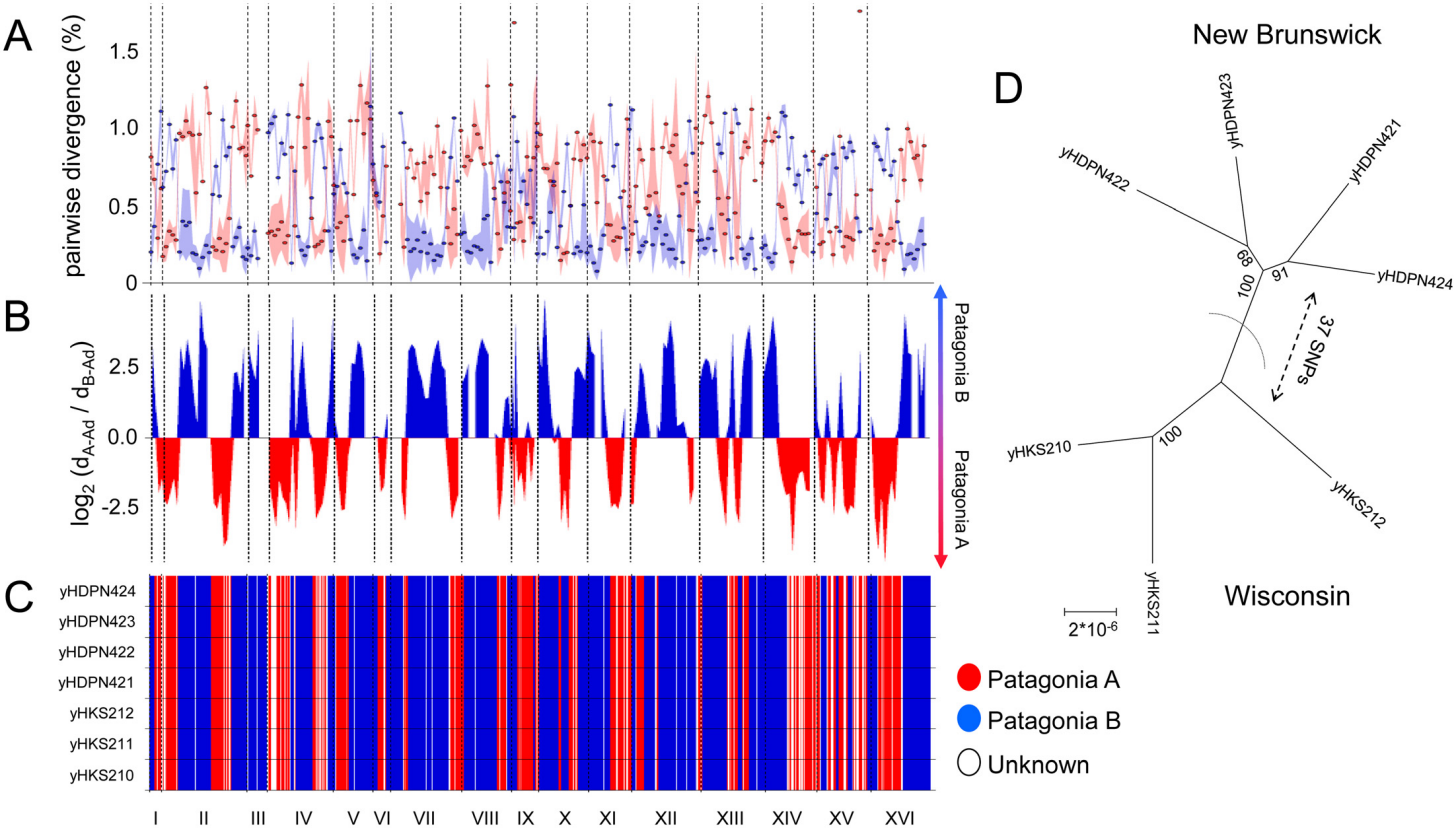
Genome-wide analysis of admixed strains. A) Pairwise nucleotide sequence divergence of the admixed strain yHKS210 compared to strains from the Patagonia A and Patagonia B populations. Average pairwise divergence comparisons are represented with red and blue dots for Patagonia A and Patagonia B, respectively. Standard deviations of pairwise divergence among Patagonia A and Patagonia B are represented by shadows, with broader regions corresponding to higher genetic diversity within populations. B) To directly visualize which population is closest to each region of the genome, we calculated the log_2_ ratio of the minimum PB-Admixed nucleotide sequence divergence (d_B-Ad_) and the minimum PA-Admixed nucleotide sequence divergence (d_A-Ad_) in 50-kbp windows. log_2_ < 0 or >0 indicate that part of the genome is more closely related to Patagonia A or Patagonia B, respectively. Regions lacking values are due to filters imposed based on coverage, data quality, or their absence in some strains (see S1 Text). C) Admixture ancestry assignment based on PCAdmix (i.e. an inference of which population is contributed that portion of the genome). Portions are defined by 20 SNPs. Blue indicates a chromosomal region inferred to share ancestry with PB-1, red indicates shared ancestry with PA-2, and white indicates that the method cannot make an inference. Roman numerals represent chromosomes. D) Unrooted ML phylogenetic tree reconstructed using SNPs. The scale bar shows the number of substitutions per site, corrected for invariant sites.

Concatenated phylogenies display the consensus topology supported by a dataset, which can obscure phylogenetic incongruence due to recombination, incomplete lineage sorting, and other biological processes. When genome-scale datasets are used, maximum support values can be obtained, even when different loci strongly support conflicting topologies [26,27]. To explore how recombination within and between populations has influenced the ancestry of *S*. *eubayanus* strains, we developed a simple and easily visualized test statistic and assessed its performance on one of the seven nearly identical admixed strains from North America (Fig 2D). First, across the genome, we plotted the average pairwise nucleotide sequence divergence (and standard deviation) of this strain compared to the Patagonia B and Patagonia A strains, clearly demonstrating regions more closely related to one population or the other (Fig 2A). This approach also revealed genomic regions of high genetic diversity within populations (Fig 2A) (e.g. the broader standard deviations of the left arm of chromosome IV among Patagonia A, and of the left arm of chromosome VII among Patagonia B strains). Next, for each window, we calculated the log_2_ of the pairwise divergence ratio using the strain with the minimum pairwise divergence value from each population. This ratio produced sharp transitions between positive and negative values, which corresponded to likely recombination breakpoints (Fig 2B). Our quantitative log_2_ ratio approach was generally concordant with a well-established program (PCAdmix) that uses a principal component analysis (PCA)-based method with hidden Markov model smoothing to assign ancestry to chromosomal regions according to the population contributing to it (Fig 2C). All seven admixed strains shared the same population ancestry in each chromosomal region, suggesting a recent radiation of this admixed lineage across the Great Lakes-Saint Lawrence Seaway.

**Fig 3:**
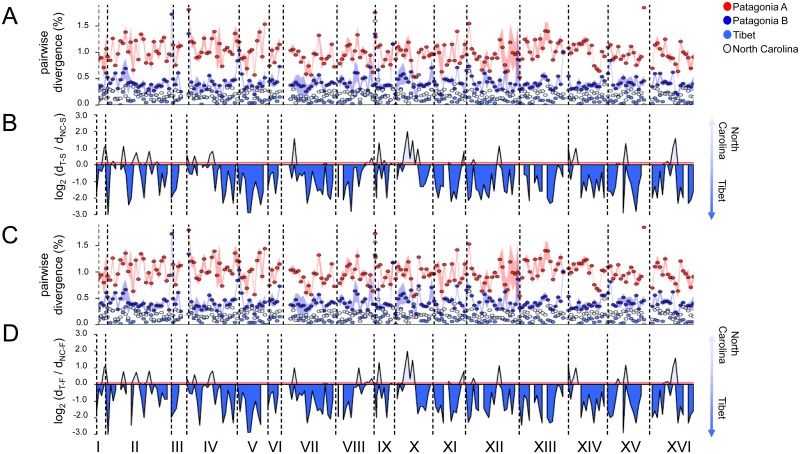
Genome-wide pairwise nucleotide sequence divergence to lager yeasts. A) and C) are pairwise nucleotide divergence comparisons to a Saaz and a Frohberg representative, respectively. Comparisons are made to the Patagonia A population, the Patagonia B strains, the two North Carolina strains, and the Tibetan representative. Dots represent average values, while standard deviations from the average are represented by the colored shadow area; red for Patagonia A, dark blue for Patagonia B, blue for Tibet (T), and light blue for North Carolina (NC). B) and D) are the log_2_ ratios of the minimum NC-Lager divergence (d_NC-X_) and the T-Lager nucleotide divergence (d_T-X_) in 50-kbp windows, where X is B) Saaz (S) or D) Frohberg (F). log_2_ < 0 or >0 indicate whether that part of the genome is more closely related to T or NC, respectively. Red lines in B) and D) are significance thresholds established by permutation tests (unbiased *P <* 0.019). Regions lacking values are due to filters imposed based on coverage, data quality, or their absence in some strains (see S1 Text). Roman numerals represent chromosomes.

**Fig 4:**
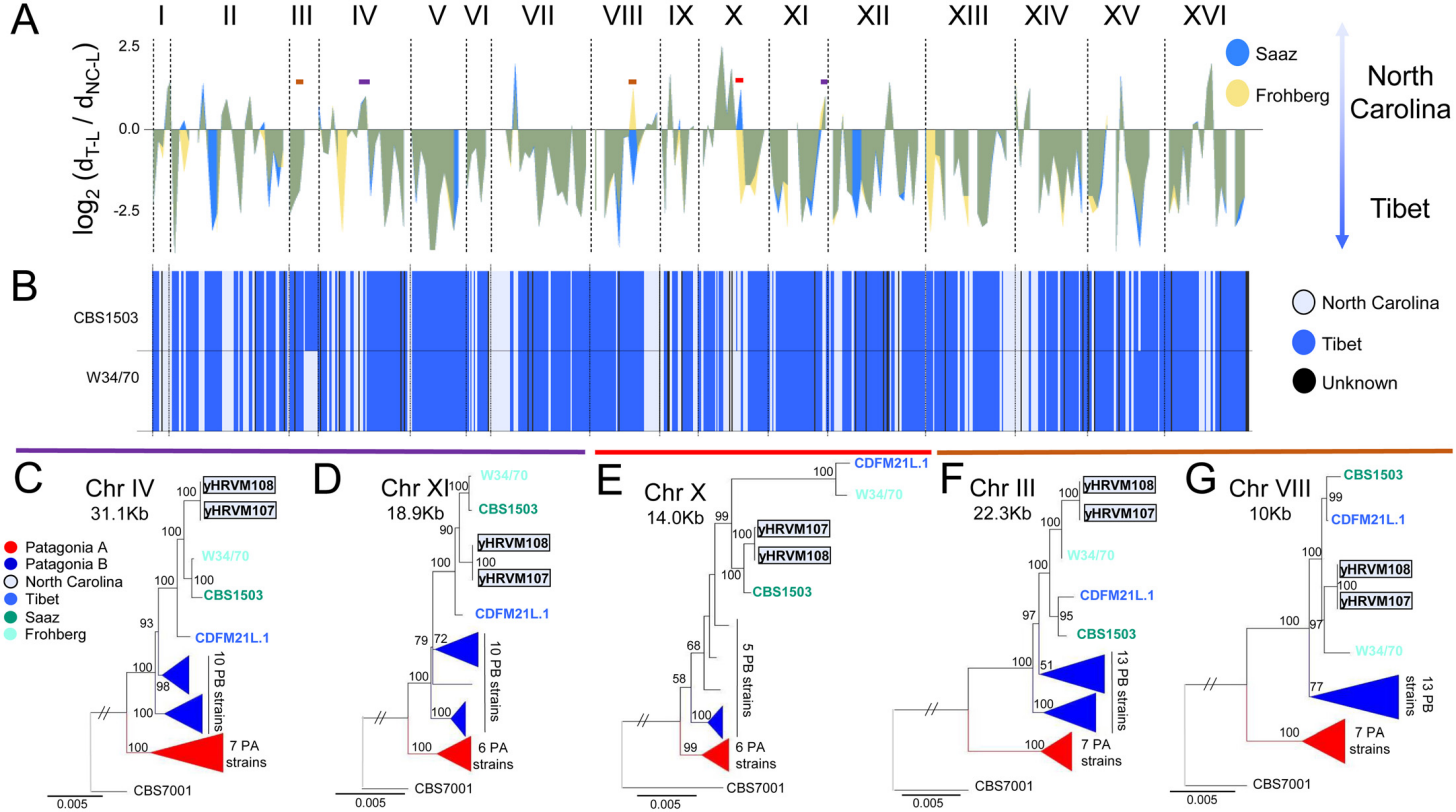
Different lager strains have different *S*. *eubayanus* alleles that were drawn from standing variation. A) Log_2_ ratios for Saaz (blue) and Frohberg (yellow) strains against Tibetan and North Carolina strains. Green values are regions where Frohberg and Saaz log_2_ ratios overlap. Phylogenetically supported regions of interest are indicated with brown bars when North Carolina (NC) was more closely related to Frohberg, while Tibet (T) was more closely related to Saaz; a red bar when NC was more closely related to Saaz and T to Frohberg; and purple bars (2 representative regions shown out of 19) when NC was the closest relatives of both the Saaz and Frohberg strains. Roman numerals represent chromosomes. B) Lager chromosome ancestry based on PCAdmix inference of which strain is most closely related to that portion of the genome. Portions are defined by 20 SNPs. Blue indicates inferred shared ancestry with T, light blue indicates shared ancestry with NC, and black marks where the method cannot infer the ancestry. PCAdmix and the log_2_ ratios produce largely overlapping results, but minor discrepancies are expected due to the differences in how the chromosomes are partitioned. C-G) ML phylogenetic trees supporting the relationships indicated by the colors of the bars, as defined above. Patagonia A and Patagonia B strains are collapsed, and the number of strains included in the reconstruction is indicated (see S4 Fig for complete documentation). The size of each alignment is shown in each panel. The scale bar shows the number of substitutions per site. Phylogenetic trees were rooted using *S*. *uvarum* (CBS7001) as the outgroup. Bootstrap values above 50 are shown to the left of their respective branches.

Similar plots were constructed to determine whether the sequenced Tibetan strain was the closest relative of lager yeasts at all loci or whether there was indeed evidence for a more complex ancestry (Fig 3). Although most of the genomes of both the Saaz and Frohberg lager representatives were more closely related to the Tibetan genome than to the North Carolina genomes (i.e. log_2_ divergence ratio values < 0), 19 regions were more closely related to the North Carolina genomes in both the Saaz and Frohberg strains (i.e. log_2_ divergence ratio > 0.118 or 0.096 for Saaz and Frohberg, respectively, unbiased *P* < 0.019, permutation test) (Figs 3B, 3D and 4A). Each of these regions was supported by PCAdmix (Fig 4B), and PCAdmix detected several additional regions where the lager strains seemed to be more closely related to the North Carolina strains than to the Tibetan strain. The log_2_ ratio statistic and PCAdmix define windows differently, either based on physical genomic distance or the number of SNPs, respectively. Therefore, as expected, the methods did not always partition genomes in exactly the same places.

Strong support for this alternative topology was confirmed by conventional phylogenetic analyses (Fig 4C and 4D, S4 Fig). In a handful of cases, a Patagonia B representative was actually more closely related to the parent of one or both of the lager lineages than the Tibetan strain was (S5 and S6 Figs). These regions could be due to incomplete lineage sorting, introgression, or different rates of evolution among wild *S*. *eubayanus* strains, but overall, they show that lager yeasts and wild strains of *S*. *eubayanus* have complex ancestries. In particular, none of the known wild isolates of *S*. *eubayanus* is the sole closest relative to lager-brewing strains. Instead, as in the case for most natural, sexually reproducing species, the data suggest an important role for outcrossing and incomplete lineage sorting in maintaining genetic variation and creating recombinant individuals.

### Standing genetic variation in *S*. *eubayanus* persists in hybrid lager-brewing yeasts

Surprisingly, comparison of the log_2_ divergence ratio values of the Saaz and Frohberg representatives against the North Carolina strains and the reference of Tibet (Fig 4, S5A and S6A Figs) highlighted at least five genomic regions where the ancestries of the Saaz and Frohberg representatives differed dramatically (Fig 4A). Several additional loci also had non-overlapping log_2_ ratios between Saaz and Frohberg, which provides further evidence of the complex ancestries of these lineages (Fig 4A). We closely inspected seven regions where the log_2_ divergence ratio, PCAdmix, or both methods suggested that the lager lineages had different alleles (Fig 4). The discordant ancestries of three of these regions were strongly supported by conventional phylogenetic analyses (Fig 4E–4G). In each case, the North Carolina strains were more closely related to one lager strain, while the Tibetan strain was more closely related to the other.

To ensure that the phylogenetic signals in these three regions were not artifacts, we closely inspected them using several orthogonal methods, including *de novo* assembly, PCR, local investigation of conflicting phylogenetic signals, examination of heterozygosity, and examination of copy-number variants. For example, the strongest phylogenetic signal for the region on chromosome X came from a 3-kbp region that placed the Frohberg and Tibetan strains sister to each other on a long branch (S7 Fig). Although this region contains a solo LTR in most strains, *de novo* assembly confirmed that the solo LTR was absent in the Tibetan and Frohberg strains and was not responsible for the phylogenetic signal. Additionally, although the Frohberg strain had multiple copies of the *S*. *eubayanus* subgenome in this region, there was no detectable heterozygosity. Heterozygosity was also too low in the other regions of phylogenetic interest to confound results (S8 Fig); indeed, overall these regions had less heterozygosity (1.08*10^−4^ and 8.49*10^−5^ heterozygous sites/bp for Saaz and Frohberg, respectively) than the genome as a whole (2.08*10^−4^ and 4.86*10^−4^ heterozygous sites/bp for Saaz and Frohberg, respectively) (S9 Fig). Differences between the regions of interest and the genome as a whole in copy-number variation (S8 and S9 Figs) and genetic diversity (S8 Fig, S2 Table) were also not the cause of the phylogenetic incongruence. Instead, we infer that the Saaz and Frohberg strains examined possess different alleles that were drawn from standing variation segregating among wild strains of *S*. *eubayanus*.

### Evidence that lager-brewing yeasts are descended from a Holarctic lineage of *S*. *eubayanus*

**Fig 5:**
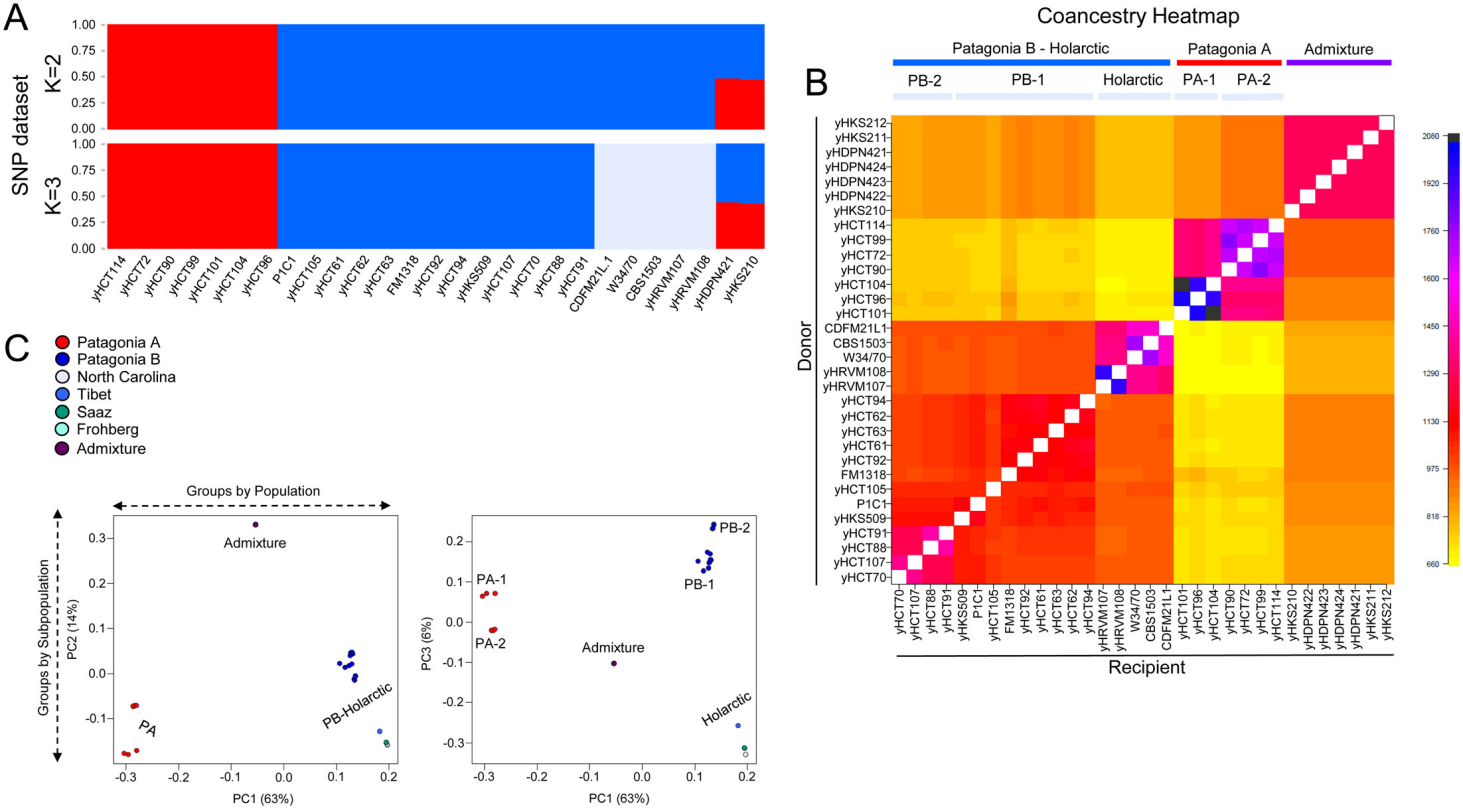
Population structure of *S*. *eubayanus*. A) Inference of the genetic clusters (*K*) and composition of individuals utilizing the WGS data in STRUCTURE. The most consistent number of genetic clusters/populations was *K* = 2 with a *ΔK*_*2*_ value = 805.70 (*K = 3* was not a significant improvement, Evanno’s report *ΔK*_*3*_ = 174.78). *K = 2* and *K* = 3 summary plots from five independent runs are shown. Each color in the bar plots represents the cluster membership coefficients. The presence of several colors in the same strain suggests admixture. B) Coancestry heatmap where darker colors indicate higher coancestry between strains. Hypothesized donor strains are on the y-axis, while hypothesized recipients are on the x-axis. Colored bars indicate populations, and grey bars indicate subpopulations. C) Principal Component Analysis (PCA) plots. PC1 versus PC2 accounted for 77% of the variation in the SNP dataset. PC1 was able to group strains by population. PC2 and PC3 highlight the complexity of the population structure by grouping the strains by subpopulation.

To delineate the number of populations of *S*. *eubayanus* and determine how well differentiated they are, we analyzed the multi-locus data from the complete strain set using STRUCTURE (S1 Text). Strains from West China were inferred to be an independent population and excluded from subsequent analyses. Analyses of WGS data using multiple methods suggested that Patagonia A and Patagonia B-Holarctic were independent populations and recovered the admixed strains (Fig 5). Although divisions beyond *K = 2* were not significant with STRUCTURE (Fig 5A), principal component and coancestry analysis with fineSTRUCTURE provided some support for dividing Patagonia A into two subpopulations (PA-1 and PA-2, Fig 5B and 5C). Similarly, these analyses split Patagonia B-Holarctic into three subpopulations, one containing most of the non-admixed strains from Holarctic ecozone (Holarctic: North Carolina, Lager, Tibet), one containing only *S*. *eubayanus* strains from South America (PB-2), and a final subpopulation containing South-American and non-South American strains (PB-1).

These analyses also provided additional information about closest relatives of the admixed and lager strains. The fineSTRUCTURE coancestry heatmap suggested that PB-1 and PA-2 were the closest relatives of the admixed strains (Fig 5B). These results were also supported by analysis of *D-*statistics, where the most significant values were obtained when PB-1 and PA-2 were tested as donors to the admixed strains (S3 Table). Analysis with PCAdmix suggested that PB-1 contributed about 58% of the genome to the admixed strains, whereas PA-2 contributed 42%, results consistent with the phylogenetic analyses and an *f4*-ratio test (S3 Table, Fig 1D). Analysis with PCAdmix for the lager genomes further suggested that strains more closely related to the Tibetan strain contributed 66% of the *S*. *eubayanus* genetic material, whereas strains more closely related to those from North Carolina contributed 34% (S1 Text). Nonetheless, we caution that the few available data are best interpreted as pointing to the existence of standing variation across the Holarctic lineage, rather than direct ancestry or admixture involving these specific extant strains.

**Fig 6:**
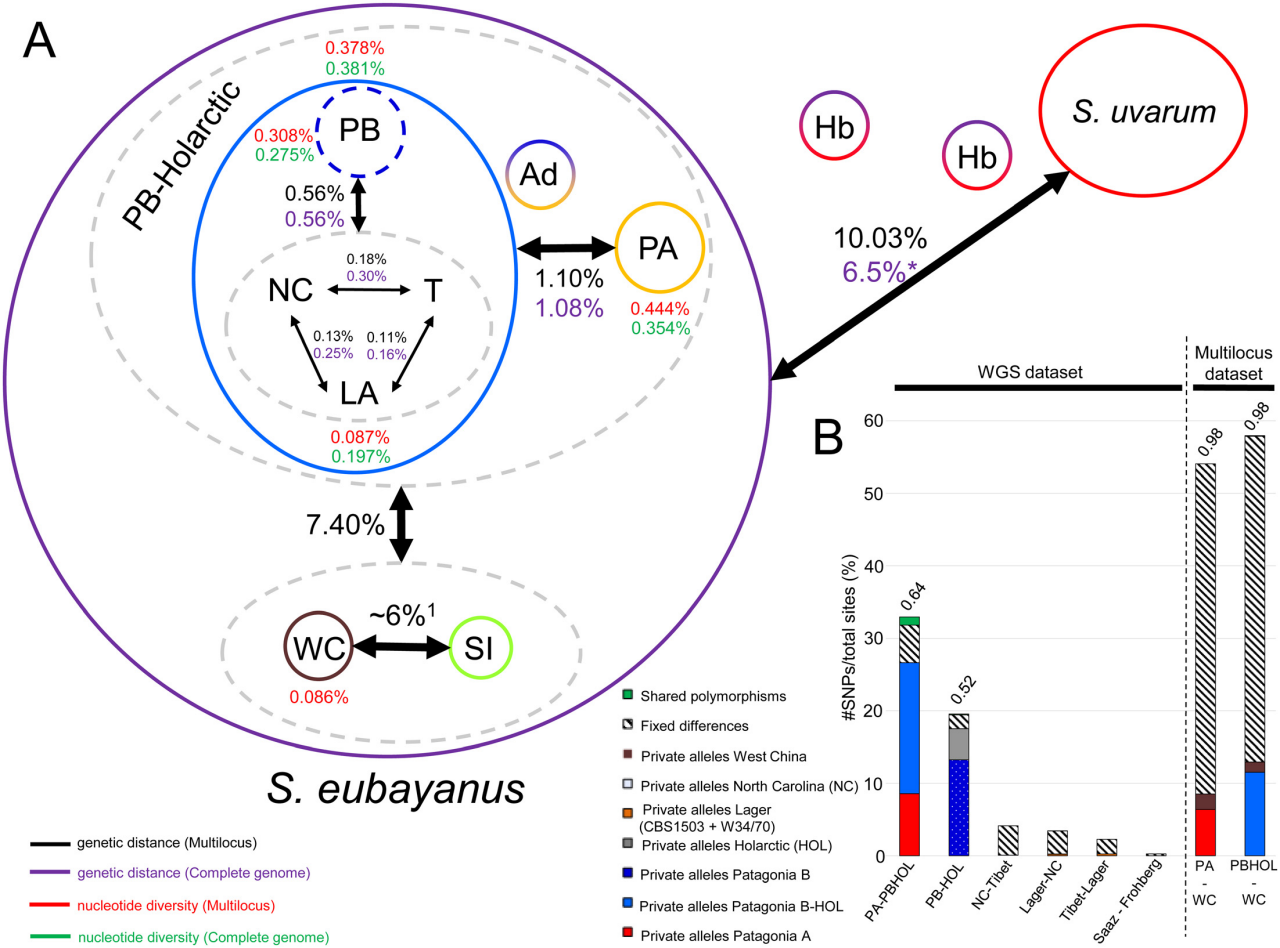
Summary statistics and genetic distances for known *S*. *eubayanus* populations. A) Black and purple values are percentages of the Tamura-Nei corrected pairwise genetic distance for the multi-locus and WGS data, respectively. Red and green percentages are nucleotide diversity statistics for the multi-locus and WGS, respectively. The asterisk indicates that the West China and Sichuan lineages could not be included in the calculation of this value. B) Percentages of private segregating alleles, fixed differences, and shared polymorphisms among SNPs found in pairwise comparisons between known populations, normalized by the total number of sites. Pairwise F_st_ values are displayed above selected bars. Admixed, Ad; Introgressed, Int; Hybrids (interspecies), Hb; *S*. *eubayanus* subgenome from Lager, LA (CBS1503 and W34/70); North Carolina, NC; Patagonia A, PA; Patagonia B, PB; Sichuan, SI; Tibet, T; and West China, WC. The genetic distances estimated by the multi-locus approach and by WGS comparisons were generally congruent, with differences ranging between 0.00% and 0.12%. Prior the estimation of pairwise genetic distance (Tamura-Nei corrected) and nucleotide diversity, we deleted gaps for each pairwise comparison. ^1^Value inferred from [21] since sequence data for the sole strain from Sichuan were not deposited in GenBank.

These results, together with the nucleotide diversity statistics (Fig 6A), the pairwise comparison of F_st_, the distribution of SNPs (Fig 6B), and phylogenetic analysis (Fig 1B) support at least four distinct populations of *S*. *eubayanus*: Patagonia A, Patagonia B-Holarctic, Sichuan, and West China (Fig 6A). The nucleotide diversities of the West China population and the Holarctic lineage were lower than either population from Patagonia (Fig 6A, S4 Table). In contrast to the other populations or groups, including the Holarctic lineage as a whole, only the 10 strains from Tibet had significantly negative values for Tajima’s D, Fu and Li's D, and Fu's F (S4 Table). The Tibet group’s Fay and Wu’s H value was not significantly different from zero (H = 0.76 *P* > 0.05, calculated using Patagonia B strains as an outgroup), which is consistent with a neutral demographic explanation, such as a recent local population expansion across the vast region of Tibet surveyed.

## Discussion

### Parallels between the biogeography of *S*. *eubayanus* and its sister species

The patterns of diversification and differentiation between *S*. *eubayanus* populations are remarkably reminiscent of those described recently for its sister species, *S*. *uvarum* (S10 Fig) [23]. Specifically, both species include early-diverging subspecies in East Asia or Australasia. Both species have two highly diverse, partially sympatric populations in Patagonia that are about 1% divergent in DNA sequence. In both cases, one of these populations is closely related to a relatively low-diversity lineage with a Holarctic distribution that gave rise to domesticated hybrid yeasts that ferment economically important products. In contrast to the process of introgression seen in domesticated strains of *S*. *uvarum*, lager yeasts were generated through allopolyploidization of *S*. *eubayanus* and *S*. *cerevisiae*. Genetic mechanisms of hybridization aside, the deep parallels between the diversifications of these two sister species in the wild suggest that similar biogeographical and ecological forces may explain their distributions. The presence of wild *S*. *uvarum* in Europe further suggests that Holarctic representatives of *S*. *eubayanus* are present, or may have been present in the past, somewhere in Europe.

### The importance of understanding the Holarctic lineage of *S*. *eubayanus*

Although non-hybrid isolates of European *S*. *eubayanus* remain elusive, we expect European strains of *S*. *eubayanus* would have relatively low genetic diversity, belong to the Holarctic lineage, and be genetically similar to isolates from Tibet and North Carolina, as well as to the parents of lager yeasts. Importantly, any European strains that might eventually be discovered will not be the closest relative to all lager yeasts at all loci because, as this study shows, standing genetic variation in *S*. *eubayanus* made it through the bottleneck of hybridization that generated modern lager yeasts. All of the currently proposed models of hybridization are compatible with this data, including multiple hybridization events [6,12–15], differential loss-of-heterozygosity among heterozygous ancestors [11], or more complicated backcrossing scenarios [9–11,14,28]. The complexity of lager yeast ancestry means that identifying the alleles relevant for specific traits may require a broad sampling of *S*. *eubayanus* genetic diversity from across the Holarctic ecozone.

In contrast to the frequent isolation of *S*. *eubayanus* from *Nothofagus* in Patagonia [5], the rare Northern Hemisphere strains of *S*. *eubayanus* described here and in other recent studies [20,21] were isolated in association with several different tree genera (S1 Fig). These findings suggest that our understanding of *S*. *eubayanus* ecology is still quite limited or may be an indication of its generalist character, as has recently been argued for *S*. *cerevisiae* [29]. Expanded sampling of substrates beyond the conventional hosts of *Quercus* and *Nothofagus* [30], even in South America [24], may be critical to gaining a fuller view of the ecological and genetic diversity of *S*. *eubayanus*.

Additional isolates will also be key for evaluating competing demographic models to explain the relationship between the Holarctic lineage and the Patagonia B population. One possibility is that a large ancestral population was split by vicariance, perhaps as the climate warmed following the last glacial period. Alternatively, long-range dispersal could have occurred between the Northern Hemisphere and South America, potentially in either or both directions. The relative diversities of the Holarctic and Patagonia B lineages and the confinement of a signature of recent demographic expansion to the Tibetan strains argue that dispersal from South America into the Holarctic may be more likely. Nonetheless, the distribution of clades defies a simple explanation and appears to require cladogenic events in multiple locations, both for *S*. *eubayanus* and its sister species *S*. *uvarum*.

### Human activity is not required to explain the dispersal of *S*. *eubayanus* to Europe

Although humans undoubtedly played a role in selecting for the allopolyploid hybrids that became lager yeasts, human activity is not required to explain the spread of wild *S*. *eubayanus* across the Holarctic ecozone. Even conservative molecular clock estimations place all *S*. *eubayanus* cladogenic events, including the origin of the Holarctic lineage, well outside of the range of written human history (S11 Fig). Moreover, no known strain is a close enough relative to the ancestor of lager yeasts to be compatible with human-mediated transfer to Europe via the Silk Road [21] or any hypothesis involving colonial era transfer to Europe from South America [5] or North America.

How yeasts migrate is still controversial. Proposed natural mechanisms include long-range dispersal by birds [31,32], short-range dispersal by insects [33], or dispersal by wind [34]. The former may be particularly relevant because some bird migration flyways from Patagonia to Greenland or Alaska, overlap with European or Asian migration routes, respectively [35]. Clear cases for human-associated yeast dispersal have been made for industrial strains of *S*. *cerevisiae*, including the dispersal of Wine/European strains to wine-making regions all over the world [36–41], as well as some interspecies hybrids used in wine production [42]. Interestingly, Wine/European strains of *S*. *cerevisiae* have retained considerable genetic diversity, perhaps because large effective population sizes were maintained and because of the semipermeable nature of the vineyard environment [41]. European strains of *S*. *paradoxus* have also been inferred to have been dispersed to North America and New Zealand, possibly in association with *Quercus* [25,39,43]. A recent population genomic analysis of the former case revealed extremely low levels of diversity and a coalescence date consistent with colonial era dispersal [44].

The genomic diversity that we observed among the admixed strains of *S*. *eubayanus* from Wisconsin and New Brunswick is also consistent with a very recent dispersal to opposite ends of the Great Lakes-Saint Lawrence Seaway. The number of inferred breakpoints (40 total crossovers, Fig 2B) is similar to the number observed in one round of meiosis in *S*. *cerevisiae* [45], and each Patagonian population seems to have contributed approximately half of their genomes. Since all seven admixed strains share the same breakpoints and have nearly identical genome sequences (of 325 variable SNPs, only 37 differentiate Wisconsin from New Brunswick, Fig 2D), they are likely descended quite recently from a single individual that underwent haploselfing after an outcrossing event and one round of meiosis. Although we cannot be certain whether this dispersal across North America and the dispersal of *S*. *paradoxus* to North America were anthropic [44], they demonstrate that recent continent-scale dispersal is detectable in yeast using WGS data. In contrast, the mean genetic distance among *S*. *eubayanus* Holarctic genomes is well over 100 times higher (0.1989% for the Tibetan, North Carolina, and lager strains versus 0.0013% for the admixed strains of *S*. *eubayanus* and 0.0009% for the North American strains of *S*. *paradoxus* from Europe).

### Conclusion

In conclusion, *S*. *eubayanus* biogeography and the origins of lager yeasts have proven more complex, but also much richer, than initially hypothesized. Here we have presented evidence that lager yeasts are derived from a relatively low-diversity lineage of *S*. *eubayanus* with a Holarctic distribution. These strains from the Holarctic lineage diversified from within one of two diverse populations found primarily in Patagonia. This pattern of diversification is similar to that of its sister species, *S*. *uvarum*. Although the *S*. *eubayanus* subgenomes of lager yeasts were drawn from the Holarctic lineage, none of the known *S*. *eubayanus* isolates is their sole nearest relative. Indeed, for the first time, we have shown that variation segregating among wild *S*. *eubayanus* persists among the allopolyploid lager-brewing yeasts. These findings strongly suggest that further sampling of the Northern Hemisphere for *S*. *eubayanus* will, not only enhance our understanding of the natural history and genetic diversity of this important species, but offer valuable insight into the sources of diversity among modern brewing strains.

## Materials and Methods

### Yeast isolation

New *S*. *eubayanus* strains were isolated from two locations in the USA, Washington State (yHKS509) and North Carolina (yHRVM107, yHRVM108), by following previously described high-sugar enrichment protocols at 10°C [46]. Four new *S*. *eubayanus* were isolated by enrichment from New Brunswick (yHDPN421-yHDPN424), Canada, as previously described [47], with the exception that the samples were incubated in liquid medium for seven months at 4°C, followed by a second culture step on solid medium for two weeks at 4°C. Strains were initially identified by PCR and Sanger-sequencing of the ITS region of the *rDNA* locus (see S1 Text). Complete results of these yeast biodiversity surveys will be reported elsewhere, and our recent publications represent less than half of the yeast strains isolated [46,47].

### Multi-locus sequence data generation

For the phylogenetic and nucleotide diversity analyses, we selected genes and intergenic sequences to integrate the maximum amount of sequencing data available from previous studies [20–22] (S1 Table). Additional genes from Patagonian and the newly isolated *S*. *eubayanus* strains were PCR-amplified and Sanger-sequenced (S4 Table). Reads from sequenced genes were assembled using the STADEN Package v1.7 [48]. The *COX2* sequence of strain CDFM21L.1 was assembled in GENEIOUS v6.1.6 using the reads retrieved by BLASTing the *S*. *eubayanus COX2* sequence against SRR1507225 from the SRA database of NCBI [21]. Individual genes of strain P1C1 were retrieved by BLASTing against its genome assembly (S1 Text). New sequences generated were deposited in GenBank under accession numbers KR871406-KR871626.

### Individual phylogenetic gene trees and supernetworks

Phylogenetic gene trees and the supernetwork were reconstructed following our previous approach [20]. The supernetwork was reconstructed using the relative average for edge weights and using the filter option to discard the splits from *PDR10* (a gene undergoing balancing selection or reciprocal introgression between some populations) (Dataset A) (S1 Text). An additional Neighbor-Net phylogenetic network was reconstructed for the SNP dataset using SplitsTree v4.12.8 [49].

### Genome sequencing and analyses

Genomic libraries for available *S*. *eubayanus* strains (S1 Table), one representative strain from the Saaz lineage of lager yeast (CBS1503), and one representative strain from the Frohberg lineage of lager yeast (W34/70) were generated as described previously [50] and sequenced using Illumina paired-end sequencing (S5 Table). Details on the identification of high-quality single nucleotide polymorphisms (SNPs) can be found in S1 Text. Illumina reads were deposited in the SRA database of NCBI under accession number SRP064616.

After removing positions with gaps in any strain, whole genome nucleotide divergence graphs were constructed by calculating the pairwise number of segregating sites per nucleotide or divergence (*d*) in windows of 50,000 bp using the PopGenome package for R [51]. To compare how closely related various strains of interest (i.e. lager or admixed) were to a portion of the genome of two defined reference strains (e.g. North Carolina and Tibet), the value of the log_2_ of the ratio of the *d* values were calculated for each window (see S1 Text).

The whole genome phylogenetic tree was reconstructed from WGS data using RAxML v8.1 [52]. For phylonetwork and population analyses, SNPs were selected using strict coverage and quality filters (details in S1 Text). Based on the comparisons of the log_2_ divergence ratios or the PCAdmix results, genomic regions of interest were extracted for phylogenetic analyses (see S1 Text). Regions of interest were extracted from whole genome assemblies reconstructed using iWGSv1.01 [53].

### Population genetics and genomics

A multi-locus concatenated alignment from Dataset A (~7.7 kbp) was generated using FASconCAT v1.0 [54]. Multi-locus concatenated alignment and WGS data were used for diversity statistics, polymorphism comparisons, and population analyses (see S1 Text). The concatenated alignment was also used to reconstruct a Maximum-Likelihood phylogenetic tree in RAxML v8.1 using the same parameters as for the individual gene trees.

A second recombinant-free concatenated alignment of the coding sequences from Dataset B (Dataset A where *IntMD*, *MET2*, and *MLS1* sequences, which had low information content, were discarded) was generated using IMGC [55] and FASconCAT. The 380 fourfold degenerate sites in this alignment were used to estimate divergence times. Divergence time reconstruction was performed as we described previously [20].

The number of populations for the SNP dataset were inferred using STRUCTURE v2.3.4 [56]. fineSTRUCTURE v2 [57] was used to generate coancestry heatmaps and to perform PCA. Parental contributions to the genomes of Wisconsin, New Brunswick, Saaz, and Frohberg strains were estimated using a hidden Markov model of evolution implemented in PCAdmix v1.0 [58], and chromosomes were partitioned according to the output results. Analyses of *f-* and *D-*statistics were performed in ADMIXTOOLS v3.0 [59].

## Supporting Information

S1 Text Supplementary materials. (DOCX)

S1 Table Strains used in this study. (XLSX)

S2 Table Genes within the regions of interest. (XLSX)

S3 Table *f3*-, *D-*statistics and *f4*-ratio tests performed in ADMIXTOOLS. (XLSX)

S4 Table Summary statistics for each population or group using multi-locus data. (DOCX)

S5 Table Summary of whole genome sequencing statistics. (XLSX)

S6 Table PCR primer sequences and conditions used in the present study. (DOCX)

S1 Fig Distribution of host trees for *S*. *eubayanus* isolates. A) Pie chart representing the tree genera from which *S*. *eubayanus* was isolated. The asterisk indicates the tree host for the 13 strains isolated by Rodríguez *et al*. [24]. B) Proportion of *S*. *eubayanus* associated to different tree orders. Populations were not designated by Rodríguez *et al*. [24], so these strains were excluded from S1B Fig. The P1C1 strain [22] lacks host information and it was not included in this figure.
(TIF)

S2 Fig Multi-locus phylogenetic supernetwork summarizes cases of likely reticulation, including introgression, gene flow, and hybridization. Phylogenetic supernetwork removing splits, excluding *PDR10* (a gene under balancing selection or reciprocal introgression) from the multi-locus dataset. Population assignment is represented by a blue, red, or brown shadow for Patagonia B-Holarctic, Patagonia A, or West China, respectively. The scale bar in the phylogenetic supernetwork represents the inferred edges’ weights using the average relative tree size option to normalize for different individual tree scales.
(TIF)

S3 Fig Individual gene trees. Each panel represent the phylogenetic tree reconstructed using A) *CCA1*, B) *FSY1*, C) *FUN14*, D) *GDH1*, E) *HIS3*, F) Intergenic region between *APP1* and *YPT53*, G) Intergenic region between *FAR8* and *RSF1*, H) Intergenic region between *MSL1* and *DSN1*, I) *MET2*, J) *MSL1*, K) *PDR10*, L) *RIP1*, and M) *COX2* sequence. Cases of introgression or incomplete lineage sorting can be observed between Patagonia A and Patagonia B strains, such as yHCT96 (Patagonia A) whose *FUN14* allele is identical to the *FUN14* allele of several Patagonia B-Holarctic strains (S9C Fig). Bootstrap values above 50 are reported to the left of their respective nodes. Scale bars represent nucleotide substitutions per site.
(PDF)

S4 Fig Phylogenetic tree reconstruction of the regions of interest without collapsing the Patagonia A and Patagonia B strains. Reconstruction of the phylogenetic tree of four of five regions of interest. These trees are identical to those shown in Fig 4 but the Patagonia A and Patagonia B clades were not collapsed. Bootstrap values above 50 are reported to the left of their respective nodes. Scale bars represent nucleotide substitutions per site.
(TIF)

S5 Fig Genome-wide log_2_ ratios of pairwise divergence of the Saaz lager representative to key populations and lineages. A) Tibet-Saaz versus North Carolina-Saaz, B) Tibet-Saaz versus Patagonia B-Saaz, and C) North Carolina-Saaz versus Patagonia B-Saaz. Arrows indicate the direction where log_2_ ratios of pairwise divergence suggest a relatively closer relationship to a particular lineage or population. The Patagonia B value reported is the lowest pairwise divergence value of all Patagonia B strains for that window. The window size is 50-kbp.
(TIF)

S6 Fig Genome-wide log_2_ ratios of pairwise divergence of the Frohberg lager representative to key populations or lineages. A) Tibet-Frohberg versus North Carolina-Frohberg, B) Tibet-Frohberg versus Patagonia B-Frohberg, and C) North Carolina-Frohberg versus Patagonia B-Frohberg. Arrows indicate the direction where log_2_ ratios of pairwise divergence suggest a relatively closer relationship to a particular lineage or population. The Patagonia B value reported is the lowest pairwise divergence value of all Patagonia B strains for that window. The window size is 50-kbp.
(TIF)

S7 Fig Region of interest on chromosome X. A) Alignment of the region of interest on chromosome X. Genes annotated in this region are represented above the alignment. Black lines represents nucleotide differences compared with the reference sequence of FM1318. Gaps are represented as white spaces; gaps in FM1318 or CBS7001 are gaps in the alignment, rather than gaps in the assemblies. B) and C) are ML phylogenetic trees reconstructed using the segments of chromosome X region indicated by the light blue and dark blue colors, respectively. Bootstrap values above 50 are reported to the left of their respective nodes. Scale bars represent nucleotide substitutions per site.
(TIF)

S8 Fig Copy number variation, heterozygosity levels, and gene annotations of the regions of interest for the Frohberg and Saaz representatives. Copy number graphs of chromosomes III, IV, VIII, X, and XI for the regions of interest for the Saaz (CBS1503) and Frohberg (W34/70) representatives. These graphs were extracted from the complete chromosome representations in S9 Fig. The coordinates correspond to the FM1318 reference genome. The lower panels correspond only to the regions demarcated by the dashed lines in the upper panels. The lower panels report the coverage values (using 1-kbp windows) for the regions of interest, gene annotations, and the absolute counts of homozygous and heterozygous SNPs (using 1-kbp windows) compared with the FM1318 reference genome.
(PDF)

S9 Fig Copy number and heterozygosity levels of *S*. *eubayanus* and Lager strains. Coverage levels normalized using the median value of coverage for the complete genome are shown for the *S*. *eubayanus* subgenome in the Saaz (CBS1503) and Frohberg (W34/70) in A) and B). Normalized coverage levels for non-hybrid strains of *S*. *eubayanus* are shown in C) CDFM21L.1, D) yHRVM108, E) yHCT61, F) yHCT70, G) yHCT96, H) yHCT114, I) yHKS212, and J) FM1318. The chromosome copy numbers of hybrids were inferred by establishing the lowest average coverage values for one copy (i.e. chromosome II of the Saaz, CBS1503, and chromosome I of the Frohberg, W34/70). Absolute counts of homozygous and heterozygous SNPs (using 50-kbp windows) compared with the FM1318 reference genome are shown in the bottom graph for each strain. High levels of heterozygosity were detected in subtelomeric regions and a handful of other regions outside of the regions of interest (S9 Fig). These regions of high heterozygosity were shared among strains, including the monosporic and homozygous strain FM1318 (panel J), suggesting they were false positives. The regions of interest (S8 Fig) have less heterozygosity (1.08*10^−4^ and 8.49*10^−5^ heterozygous site/bp for Saaz and Frohberg, respectively) than the average heterozygosity detected genome-wide (2.08*10^−4^ and 4.86*10^−4^ heterozygous site/bp for Saaz and Frohberg, respectively). Moreover, heterozygosity was not positively correlated with an increase in the number of copies inferred (linear regression r^2^ = 0.097, p-value = 0.381). Nucleotide diversity levels of the annotated genes within the regions of interest (S8 Fig, S2 Table) were, in general, lower than the average value found genome-wide among the strains from the Patagonia A-Patagonia B-Holarctic clade (0.57%). For 14 of 44 genes the values were higher but less than twice the genome-wide diversity values. Based on comparisons to the multi-locus dataset, the false positive rate of our pipeline at calling non-heterozygous sites was low (4.63*10^−5^SNPs/site) and not sufficient to influence conclusions regarding the regions of interest.
(PDF)

S10 Fig *S*. *eubayanus* and *S*. *uvarum* phylogenetic tree comparison. *S*. *eubayanus* and *S*. *uvarum* phylogenetic trees are shown in A) and B), respectively. Color bars represent populations for each species, and are colored according to the colors used in the previous *S*. *eubayanus* phylogenetic tree figures. Demographically similar populations of *S*. *uvarum* use the analogous colors from *S*. *eubayanus*. The multi-locus *S*. *eubayanus* phylogenetic tree is from Fig 1B, while the *S*. *uvarum* phylogenetic tree is reconstructed from Almeida *et al*. [23] after correcting branch lengths for the presence of invariant sites. Phylogenetic trees were rooted using *S*. *uvarum* (CBS7001) or *S*. *eubayanus* (FM1318) as the outgroup in A) and B), respectively. The scale bar represents the number of substitutions per site.
(TIF)

S11 Fig Time-calibrated phylogenetic tree. Blue, red, and brown bars indicate the population designation for Patagonia B-Holarctic, Patagonia A, and West China, respectively. The scale bar represents divergence time in thousands of years (kya).
(TIF)

S12 Fig The recombinant TTH27L.1 *MLS1* gene sequence is likely an artifact. The TTH27L.1 *MLS1* sequence reported in GenBank appeared to be a recombinant version between *S*. *eubayanus* West China and *S*. *uvarum*. Black and gray colors represent polymorphisms from *S*. *uvarum* and *S*. *eubayanus* West China, respectively. The phylogenetic trees in S2 Fig of Bing *et al*. [21] suggested that the TTH27L.1 and PYCC 6148^T^ (= CRUB 1568^T^) *MLS1* sequences were not recombinant; however, the sequences deposited in GenBank (KF892364 and KF892348, respectively) appeared to be recombinant. Our copy of the strain PYCC 6148^T^ did not possess a recombinant *MLS1*, but we could not check the strain TTH27L.1 because it is not available for study. We noted that the apparent recombination point for both strains is at the junction of the promoter and coding sequence, so we suspect that errors were introduced *in silico* while the sequences were uploaded to GenBank or when multiple Sanger sequencing reads were assembled. Absent further direct verification of TTH27L.1 *MLS1*, we suggest that the apparent recombination is likely an artifact.
(DOCX)
