# Supplementary material for: Complex Ancestries of Lager-Brewing Hybrids Were Shaped by Standing Variation in the Wild Yeast Saccharomyces eubayanus
Source: PLoS Genet. 2016 Jul 6;12(7):e1006155. doi: 10.1371/journal.pgen.1006155 (PMC4934787; doi:10.1371/journal.pgen.1006155)
Supplement: S1 Text — (DOCX) [file pgen.1006155.s001.docx]

Supplementary Materials for

**Complex ancestries of lager-brewing hybrids were shaped by standing variation in the wild yeast *Saccharomyces eubayanus***

**Authors:** David Peris, Quinn K. Langdon, Ryan V. Moriarty, Kayla Sylvester, Martin Bontrager. Guillaume Charron, Jean-Baptiste Leducq, Christian Landry, Diego Libkind, Chris Todd Hittinger

correspondence to: Chris Todd Hittinger ([cthittinger@wisc.edu](mailto:cthittinger@wisc.edu)).

**This PDF file includes:**

Supplementary Text

**Table of Contents**

Yeast identification and spore viability test [3](#Yeast_identification)

Illumina read manipulation, SNP calling, and

ancestry inference [3-6](#Illumina_reads)

Tree reconstruction for genomic regions of interest [6-7](#Tree_reconstruction_int_regions)

Population genetic and genomic analysis [8-](#Pop_gen_genomic_anal)10

Conflicting data in the multi-locus dataset suggests

a complex genomic structure [10-1](#Conflicting_data)1

Extensive reticulate evolution in *S. eubayanus* and its

hybrids [11-1](#Reticulation)3

*S. eubayanus* host distribution [14-1](#Host_distribution)5

Postzygotic isolation among Patagonia A and Patagonia B [1](#Sporulation)5

References [15-](#References)20

Description of supporting information [20-2](#Tables)4

Supplementary figures [25-](#Supp_Figures)70

**Supplementary Text**

**Yeast identification and spore viability test**

The ITS region of the *rDNA* locus (containing ITS1, 5.8S, and ITS2) was amplified by colony PCR and Sanger-sequenced. Putative identifications of *S. eubayanus* isolates were made using the ITS sequences as a BLAST query against the NCBI nr/nt database, which includes the ITS sequence of the type strain of *S. eubayanus* [1].

Sixteen tetrads from a representative strain from each of the New Brunswick, North Carolina, and Wisconsin locations were dissected to analyze their spore viability. Tetrads were microdissected and micromanipulated using the SporePlay Dissector (Singer Instruments, UK). yHDPN421 and yHKS211 tetrad formation was observed on YPD plates (2% glucose, 2% peptone, 1% yeast extract, and 2% agar) after 6 days at room temperature. However, it was necessary to pre-culture yHRVM108 overnight in 5mL of pre-sporulation medium (0.8% yeast extract, 0.3% peptone, and 10% glucose), followed by plating on sporulation plates (1% potassium acetate, 0.1% yeast extract, 2% agar, and 0.05% glucose) for 7 days at room temperature.

**Illumina read manipulation, SNP calling, and ancestry inference**

Adapters were trimmed from Illumina raw reads using CUTADAPT v1.2.1 [2]. Paired-end reads were mapped to the *S. eubayanus* FM1318 (a diploid generated by the self-mating of a monosporic derivative, of the type strain, CRUB1568^T^) reference genome sequence [3] using BWA v0.7.12 with “bwa mem” default settings [4]. In the case of lager yeast sequences, a combined reference genome consisting of *S. eubayanus* FM1318 and *S. cerevisiae* S288c was used to distinguish the *S. cerevisiae* and *S. eubayanus* component of each strain. Only the *S. eubayanus* portion of the lager genomes was retained for downstream analyses. The resulting SAM files were viewed and sorted using SAMTOOLS v0.1.19 [5], filtering for high quality reads “samtools view -q 20 –bhSu”, followed by “samtools sort”. PCR duplicates were removed with PICARD v1.98 MarkDuplicates.jar using the settings “REMOVE_DUPLICATES = True; AS = true; VALIDATION_STRINGENCY = SILENT”. Read groups were set using PICARD AddOrReplaceReadGroups.jar with settings “VALIDATION_STRINGENCY = SILENT; SORT_ORDER = coordinate; CREATE_INDEX = true”. Single nucleotide polymorphisms (SNPs) were called using the GATK v3.3-0 haplotype caller using the setting “--genotyping_mode DISCOVERY -mbq 10 -stand_emit_conf 31 ‑stand_call_conf 31” [6]. Genome coverage was measured using BEDTOOLS v2.18.1 genomeCoverageBed -d -ibam [7] and manipulated using custom R scripts. The VCF output of GATK was converted into FASTA format using a custom python script. For both reference genomes, repeats were masked using REPEATMASKER v4.0.5 (<http://www.repeatmasker.org/>), and this masked genome was used to create a strain specific FASTA file. This VCF-FASTA conversion script uses the reference genome as a template and replaces the called variant with the SNP reported by GATK. The presence of heterozygous sites in the sample were coded according to their IUPAC ambiguity codes. In downstream analyses, we considered only the homozygous SNPs due to the low levels of heterozygosity. Insertions and deletions were masked by replacing the genomic sequence with a number of Ns corresponding to the length of the indel called. FASTA files for each sample were generated by masking regions with extremely high coverage (i.e. values greater than the 99.9th percentile of genome-wide coverage) and by masking regions with low coverage (i.e. either regions below 10X coverage or, for genomes with low coverage, below the 10th percentile of genome-wide coverage). These masked regions were replaced by Ns prior to downstream analyses, a procedure that also removed all missing data. A median false positive rate of 4.63*10^-5^SNPs/site was estimated by comparing the sequences generated by this pipeline to the Sanger sequences used in our multi-locus Dataset A.

To compare how close portions of the genome of query strains (i.e. Lager or Admixed) were to reference populations (e.g. Tibet or North Carolina strains or all Patagonia A or all Patagonia B), we used the raw pairwise divergence (Fig 2A, 3A,C) to calculate the log_2_ divergence ratio graphs for each window as follows:

*f(x) = log_2_ (d_ik_ /d_jk_)*

where *i*,*j* are the two reference genomes, and *k* is the genome of the query strain. log_2_ > 0 indicates that the portion of genome corresponding to that window is more closely related to *j*, and log_2_ < 0 indicates that the more closely related strain for that portion of the genome is *i*. To determine whether deviations from zero were significant for the number of tests being performed across the genome, a permutation test was implemented using 100 pseudoreplicates of randomized genomes analyzed in 50-kbp windows. The maximum of the distribution of the maximum log_2_ values for these permuted genomes was used to establish the threshold beyond which regions were significantly more closely related to strain *j* (unbiased *P* < 0.019, a threshold calculated as *P* = [(*m* x *α*) + 1]/(*m* + 1) where *m* is the number of randomized genomes and *α* is the probability of a type I error) (Saaz = 0.118, Frohberg = 0.096, Fig 3B,D). This approach was compared to PCAdmix v1 analysis [8], which estimates the proportion of ancestry by using principal component analysis (PCA) and a hidden Markov model. FASTA files were converted to PCAdmix input format, which is based on BEAGLE phased format, using custom scripts. We performed two runs: i) North American strains (Wisconsin and New Brunswick) were considered the “admixed” group, whereas PA-2 and PB-1 strains were the ancestral groups; ii) lager strains (CBS1503 and W34/70) were considered the “admixed” group, whereas Tibet and North Carolina were considered the ancestral groups. The run was performed with default values, which picks 20 contiguous SNPs for each window, and we represented the chromosome contributions using a custom R script.

**Tree reconstruction for genomic regions of interest**

Genomic regions of interest were selected for phylogenetic analysis based on the log_2_ divergence ratio comparisons and PCAdmix. We defined regions of interest as those showing lager strains as potentially more closely related to North Carolina strains than to the strain from Tibet or where Saaz and Frohberg strains differed in their potential relationships with the strains from North Carolina and Tibet (i.e. where Saaz was more closely related to one of these groups, and Frohberg was more closely related to the other). We detected several statistically significant regions showing lager strains as potentially more closely related to North Carolina strains and report two representative examples (out of 19) in Fig 4A. For regions showing different ancestries between Saaz and Frohberg, we reported the regions strongly supported phylogenetically. A maximum-likelihood phylogenetic tree for each region of interest was reconstructed in RAxML v8.1 [9], performing 100 iterations to search for the best tree, using the model GTRGAMMA. Bootstrap branch support was assessed by performing 1000 pseudoreplicates using the same model parameters as above.

Genome assemblies were generated using iWGS v1.01 [10]. In short, iWGS is a wrapper for a pipeline of genome assembly packages and quality control tools that allows the user to explore and evaluate several alternative genome assemblies. In this case, it was used to recover and evaluate the best assemblies for the regions of interest. Contigs containing these regions were recovered from the genome assemblies by BLAST. When regions contained gaps, as was the case for some Patagonian strains with low coverage, we closed them using the reference-based assemblies described above; strains that still had a large amount of missing data were excluded from further analyses. Regions of interest were annotated in Geneious R6 (S7 Fig, S8 Fig).

In total, five regions of phylogenetic interest were investigated manually (on chromosomes III, IV, VIII, X and XI) (Fig 4). The log_2_ ratios suggested clear differences between the Saaz and Frohberg lineages for a region of chromosome X (Fig 4), but this region posed several challenges that required close inspection. First, this region encodes the gene *TDH2* (S7 Fig), which has a two close paralogs in the genome, *TDH3* and *TDH1.* Since *TDH2* is nearly identical to these genes for long stretches, it was difficult to assemble *de novo* complete contigs for this region in most strains. Second, the Frohberg (W34/70) genome has a translocation between the *S. cerevisiae* and *S. eubayanus* subgenomes at *TDH2* coding sequence [11], which we confirmed in our copy of W34/70 using two different *de novo* assemblers (MaSuRCA and SPAdes) (S8 Fig). Third, most strains have a solo long terminal repeat (solo LTR) on the right side of this region, near where most of the phylogenetic signal was found (S7 Fig). The solo LTR was observed in all strains, except the Tibetan and W34/70 strains. In the case of W34/70, we confirmed the absence of the LTR by PCR amplification of the region, using primers described in S4 Table; we also confirmed its presence in FM1318. Unfortunately, we could not manually test the strain from Tibet, but the *de novo* assembly in this region contained no hint of the solo LTR, nor did it have an assembly gap in its place.

**Population genetic and genomic analyses**

Basic population genetic statistics of the concatenated multi-locus dataset A were calculated in DnaSP v5 [12]. Nucleotide diversity statistics (S2 Table) of individual genes from the regions of interest were analyzed using the PopGenome package in R [13]. Except for *SNT1* on chromosome III, none of the genes from the regions of interest showed unusually high nucleotide diversity values (>2X the diversity of strains in the Patagonia A-Patagonia B-Holarctic clade, 0.57). We observed some genes with low nucleotide diversity values (≤0.2%), most of them related to ribosomal functions, except for *GNA1* (an essential gene in *S. cerevisiae* [14]) and *TDH2,* which is involved in catalyzing the conversion of glyceraldehyde-3-phosphate to phosphoenolpyruvate, an important step in glycolysis (S2 Table).

The WGS alignment dataset with gaps removed was analyzed in PopGenome to calculate genetic diversity statistics and the fixation index, F_ST_ (Fig 6). Polymorphisms from the whole genome dataset were calculated in DnaSP v5. We found 124 SNPs among the Wisconsin strains and 122 SNPs among the New Brunswick strains. 37 SNPs differentiated the strains based on geography (Fig 2C), and 42 SNPs were shared between the Wisconsin and New Brunswick strains. In the case of the Saaz and Frohberg representatives, these two strains were differentiated by 1757 SNPs in their *S. eubayanus* subgenomes. 630 SNPs were observed between the two North Carolina strains, while P1C1 and yHKS509 had 31016 SNPs. This data suggests P1C1 and yHKS509 strains have been diverging for longer than the admixed, lager, and North Carolina strains.

To delimit the number of populations of *S. eubayanus* represented by the known strains, we used the program STRUCTURE v2.3.4 [15–17] after picking 10,000 SNPs from the VCF data and converting them to the STRUCTURE input format using custom scripts. We determined the number of populations supported by two datasets, first for all *S. eubayanus* strains using the multi-locus dataset, and second with the 10,000-SNPs dataset for the available *S. eubayanus* strains (our Patagonian and North American strains, the two lager strains, the New Zealand strain, and one Tibetan strain). When we ran STRUCTURE with the complete admixture group, we observed that it was assigned as an independent population. Numerous assumptions made by STRUCTURE are violated by fungal systems; in this case, the 7 nearly identical admixed strains possess a unique constellation of alleles and seem not have undergone additional outcrossing events after all variation was purged by haploselfing. Selecting two representative admixed strains (one from Wisconsin and one from New Brunswick) allowed us to avoid the bias introduced by their life cycles. We tested *K* clusters from 1 to 10, assuming an admixture model, with a 10000-iteration burn-in and 100000 iterations of sampling. Five independent runs were performed for each *K* cluster. The STRUCTURE output was used as input for STRUCTURE HARVESTER web v0.6.94 [18] to select the most likely number of populations. STRUCTURE HARVESTER output files were aligned in CLUMPP v1.1.2 [19] and visualized in STRUCTURE PLOT package for R [20]. The most consistent number of genetic clusters/populations was *K*=2 with a *ΔK_2_* value = 805.70 (*K=3* was not a significant improvement, Evanno’s report *ΔK_3_* = 174.78).

**Conflicting data in the multi-locus dataset suggests a complex genomic structure**

Using the multi-locus dataset A, we did not detect gene flow or incongruent data between West China and Patagonia-Holarctic strains (S2 Fig). The strains were clearly split into three groups: i) *S. uvarum*, ii) West China, and iii) Patagonia-Holarctic. Three triple hybrid “*S. bayanus*” brewing contaminant strains were located between *S. uvarum* and the Patagonia-Holarctic group with a distance to *S. uvarum* or *S. eubayanus* that depended on the contribution from each parent to the genes in our dataset (S3 Fig). Only in the case of TTH27L.1 did we find an additional edge indicating the presence of conflicting data. A close inspection of individual gene trees showed *MLS1* to be the conflicting gene tree. The *MLS1* gene in TTH27L.1 appeared to be a recombinant allele between *S. uvarum* and *S. eubayanus*, but this sequences is likely an artifact introduced prior to its deposition in GenBank (S3J Fig, S12 Fig). yHCT94 clustered with Patagonia A for the *MLS1* gene (S3J Fig).

The *PDR10* gene, which was removed from all multi-locus phylogenetic analyses, showed some strains from PB-2 group (yHCT70, yHCT88, yHCT91, yHCT105, and yHCT107) clustering with Patagonia A (S3K Fig). Similarly, we found that the *PDR10* gene for some Patagonia A strains, including the PA-2 group strains yHCT72, yHCT90, and yHCT99, clustered with Patagonia B strains. *PDR10* encodes an ATP-binding cassette (ABC) transporter that functions as a multidrug transporter [21]. The presence of both alleles in both populations could be due to balancing selection or reciprocal introgression, possibly driven by selection for drug resistance in the environment. Regardless of the precise evolutionary scenario, these significant departures from neutrality make it an unreliable indicator of the organismal phylogeny, an inference confirmed by the analyses of WGS data (Fig 1C).

The strains from North Carolina (yHRVM107 and yHRVM108), Tibet, and lager clustered with the Patagonia B strains for all genes, except for *FSY1*, where they formed an independent clade that was separated from both Patagonian populations (S9B Fig). Although we did not detect any unusual signatures of selection at *FSY1* and retained it in all analyses, we note that it is the only subtelomeric gene in the multi-locus dataset. The strains from Tibet, lager, and North Carolina had similar *COX2* gene sequences (recombinant between *S. eubayanus* and *S. uvarum* as described in [1]); the Tibetan *COX2* gene sequence was identical to the lager *COX2* sequences, while the North Carolina *COX2* sequences had one nucleotide difference (S9M Fig). We were not able to get complete mitochondrial genomes from WGS assemblies; however, the hypothesis of a close mitochondrial relationship between Tibet and lager has recently been confirmed [22].

**Extensive reticulate evolution in *S. eubayanus* and its hybrids**

To get a deeper picture of coancestry among *S. eubayanus* strains and to further evaluate the STRUCTURE results, we converted the FASTA dataset with the complete set of SNPs to a PHASED format, the input format of fineSTRUCTURE v2 [23], using custom scripts. To reconstruct the coancestry heatmap with the linkage model and to perform a principal component analysis of the SNP dataset (Fig 5), fineSTRUCTURE was run with default paremters, except ¨-ploidy 1” due to the low heterozygosity in the dataset; the genetic distance map was inferred by applying the specific genetic distance for each chromosome described on the SGD database.

To detect and quantify the directionality and contributions of gene flow among the described populations and subgroups (Fig 5), we calculated the *f3*-, *D*-, and *f4*-statistics implemented in ADMIXTOOLS v3.0 [24]. The PED format generated in PLINK was converted to EIGENSTRAT format using the “convertf” command implemented in ADMIXTOOLS. Principal components (PC1 vs PC3) suggested 5 small subgroups: the Holarctic subgroup, including the lager, North Carolina and Tibetan strains; two Patagonia B subgroups, PB-1 and PB-2; all seven admixed strains (Wisconsin and New Brunswick); and two Patagonia A subgroups, PA-1 and PA-2 (Fig 5C). The two new strains, yHKS509 from Washington State and P1C1 from New Zealand, clustered closely with the PB1 subgroup (Fig 5B). The *f3*-statistics for each subgroup had positive values, which could be interpreted to suggest the absence of gene flow among the subgroups tested (S3 Table). However, *f3*-statistics can also be positive if the testing population (either admixed or lager strains) has experienced a population-specific genetic drift after the admixture event [24]; in both cases, the known strains of these lineages experienced extreme genetic bottlenecks and retain very limited genetic diversity. The *D-*statistics, calculated with ADMIXTOOLS, agree with the results from fineSTRUCTURE. The more significant Z-scores were obtained for contributions of PB-1 and PA-2 to the admixture strains. Similarly, the *D-*statistics produced the most significant *Z-*scores when the North Carolina and Tibetan strains were evaluated as contributing to the lager strains (S3 Table). For the *f4*-ratio test, ADMIXTOOLS calculated that 58% of the genome of the admixed strains was contributed by PB-1, and 42% was contributed by PA-2 (S3 Table), values that agree with PCAdmix (Fig 1D). However, in the case of the lager strains, we could not infer *α* (i.e. values were outside the interpretable range of 0 to 1, and the standard errors were above 1). *f-*statistics are based on genetic drift [24–26], and we suspect that the low number of strains available from North Carolina (2 nearly identical strains) and Tibet (a single strain) were insufficient to properly estimate *α*.

As suggested by the multi-locus data, a complex evolutionary history for Patagonia A and B was also observed in the SNP phylonetwork (Fig 1D). fineSTRUCTURE detected a higher coancestry between Patagonia A strains and FM1318 (Fig 5B). A close inspection of SNP counts in Patagonia A strains and FM1318 reveal at least two chromosomal regions, chromosome VII and chromosome XII, with an extremely low number of SNP differences (S9G Fig). The high number of SNPs in these regions detected when comparing FM1318 to other strains from Patagonia B (S9E,F Fig) and Holarctic strains (S9A-D Figure) suggests a recent introgression of these regions from PA-1 into FM1318 (Fig 5B). The number of SNP differences were higher when FM1318 was compared with PA-2 (S9H Figure), suggesting PA-2 was not the Patagonia A donor.

***S. eubayanus* host distribution**

To further understand the mechanisms of diversification in yeasts in general, and *Saccharomyces* in particular, consideration of the ecological distribution of these isolates is important. Unfortunately, scarce ecological information and sampling biases, have created, at best, an incomplete picture of yeasts habitats (for recent reviews, see [27,28]). Extreme caution is therefore warranted about the following analysis of the currently known ecological distribution of *S. eubayanus*.

The host distribution of the known *S. eubayanus* strains is biased to the tree order Fagales: Fagaceae, Juglandaceae, and Nothofagaceae (χ^2^ test df=4, *P* = 2.2e-16) (S1A Fig). The ecological distribution of previously described populations, as well as some new strains, are mostly limited to Fagales (35 of 42 strains) (S1A Fig). The Tibetan strains are more broadly distributed, being found in association with Malpighiales and Rosales trees, and are not significantly associated with a particular tree genus. North American strains also had a broader apparent habitat. One admixed strain was isolated from Sapindales (S1 Table, S1B Fig). The Washington (yHKS509) and the North Carolina strains were found in association with trees of the order Pinales. Recent *S. eubayanus* isolates from South America were also found in association with Pinales, specifically *Araucaria araucana* [29]. Patagonia A and Patagonia B strains were associated with *Nothofagus* (Fagales) (Fisher’s exact test, *P*=0.0184 and *P*=7.8e-04, respectively). We did not include the strains isolated from *Araucaria araucana* (Pinales) in our population analyses because there is insufficient genetic information to make definitive population assignments [29]. Nonetheless, it is clear that even *S. eubayanus* from South America are not limited to Fagales, and these recent discoveries further highlight biases in sampling. In contrast, the West China strains were significantly associated with *Quercus* (Fisher’s exact test, *P*=1.2e-07) (S1 Table, S1B Fig). Collectively, these analyses suggest that the ecological distribution of *S. eubayanus* is broader and more complex than previously hypothesized. Given its possible generalist niche and broad geographical distribution, screening additional hosts and regions may be productive.

**Postzygotic isolation among Patagonia A and Patagonia B**

Admixed strains offer opportunities to study for the presence of incompatible alleles. For this reason, we studied the spore viability of a strain from Wisconsin and a strain from New Brunswick. yHDPN421 had 50% spore viability, while yHKS211 had 80% spore viability. 100% is the expected spore viability of pure Patagonia A or B strains, as we observed for yHRVM108. The reduced viability in yHDPN421 and yHKS211 might provide hints of partial genetic incompatibilities between the Patagonia A and Patagonia B populations.

**References**

1. Peris D, Sylvester K, Libkind D, Gonçalves P, Sampaio JP, Alexander WG, Hittinger CT (2014) Population structure and reticulate evolution of *Saccharomyces eubayanus* and its lager-brewing hybrids. Mol Ecol 23: 2031-2045.

2. Martin M (2011) Cutadapt removes adapter sequences from high-throughput sequencing reads. EMBnet journal 17: 10-12.

3. Baker E, Wang B, Bellora N, Peris D, Hulfachor AB, Koshalek JA, Adams M, Libkind D, Hittinger CT (2015) The genome sequence of *Saccharomyces eubayanus* and the domestication of lager-brewing yeasts. Mol Biol Evol 32: 2818-2831.

4. Li H, Durbin R (2009) Fast and accurate short read alignment with Burrows-Wheeler transform. Bioinformatics 25: 1754-1760.

5. Li H, Handsaker B, Wysoker A, Fennell T, Ruan J, Homer N, Marth G, Abecasis G, Durbin R, Genome Project Data Processing Subgroup (2009) The Sequence Alignment/Map format and SAMtools. Bioinformatics 25: 2078-2079.

6. McKenna A, Hanna M, Banks E, Sivachenko A, Cibulskis K, Kernytsky A, Garimella K, Altshuler D, Gabriel S, Daly M, DePristo MA (2010) The Genome Analysis Toolkit: a MapReduce framework for analyzing next-generation DNA sequencing data. Genome Res 20: 1297-1303.

7. Quinlan AR, Hall IM (2010) BEDTools: a flexible suite of utilities for comparing genomic features. Bioinformatics 26: 841-842.

8. Henn BM, Botigué LR, ravel S, ang W, risbin A, yrnes JK, adhlaoui-Zid K, alloua PA, oreno-Estrada A, ertranpetit J, ustamante CD, omas D (2012) Genomic ancestry of North Africans supports back-to-africa migrations. PLoS Genet 8: e1002397.

9. Stamatakis A (2014) RAxML version 8: a tool for phylogenetic analysis and post-analysis of large phylogenies. Bioinformatics 30: 1312-1313.

10. Zhou X, Peris D, Hittinger CT, Rokas A (2015) *in silico* Whole Genome Sequencer & Analyzer (iWGS): a computational pipeline to guide the design and analysis of de novo genome sequencing studies. bioRxiv doi: http://dx.doi.org/10.1101/028134.

11. Nakao Y, Kanamori T, Itoh T, Kodama Y, Rainieri S, Nakamura N, Shimonaga T, Hattori M, Ashikari T (2009) Genome sequence of the lager brewing yeast, an interspecies hybrid. DNA Res 16: 115-129.

12. Librado P, Rozas J (2009) DnaSP v5: a software for comprehensive analysis of DNA polymorphism data. Bioinformatics 25: 1451-1452.

13. Pfeifer B, Wittelsbürger U, Ramos-Onsins SE, Lercher MJ (2014) PopGenome: an efficient Swiss Army Knife for population genomic analyses in R. Mol Biol Evol 31: 1929-1936.

14. Mio T, Yamada-Okabe T, Arisawa M, Yamada-Okabe H (1999) *Saccharomyces cerevisiae* GNA1, an essential gene encoding a novel acetyltransferase involved in UDP-N-acetylglucosamine synthesis. J Biol Chem 274: 424-429.

15. Pritchard JK, Stephens M, Donnelly P (2000) Inference of population structure using multilocus genotype data. Genetics 155: 945-959.

16. Falush D, Stephens M, Pritchard JK (2003) Inference of population structure using multilocus genotype data: linked loci and correlated allele frequencies. Genetics 164: 1567-1587.

17. Hubisz M, Falush D, Stephens M, Pritchard JK (2009) Inferring weak population structure with the assistance of sample group information. Mol Ecol Resour 9: 1322-1332.

18. Earl D, vonHoldt B (2012) STRUCTURE HARVESTER: a website and program for visualizing STRUCTURE output and implementing the Evanno method. Conservation Genet Resour 4: 359-361.

19. Jakobsson M, Rosenberg NA (2007) CLUMPP: a cluster matching and permutation program for dealing with label switching and multimodality in analysis of population structure. Bioinformatics 23: 1801-1806.

20. Krishnan R, R S, B B, Naik V (2014) STRUCTURE PLOT: a program for drawing elegant STRUCTURE bar plots in user friendly interface. SpringerPlus 3: 431.

21. Rogers B, Decottignies A, Kolaczkowski M, Carvajal E, Balzi E, Goffeau A (2001) The pleitropic drug ABC transporters from *Saccharomyces cerevisiae*. J Mol Microbiol Biotechnol 3: 207-214.

22. Okuno M, Kajitani R, Ryusui R, Morimoto H, Kodama Y, Itoh T (2016) Next-generation sequencing analysis of lager brewing yeast strains reveals the evolutionary history of interspecies hybridization. DNA Res 23: 67-80.

23. Lawson DJ, Hellenthal G, Myers S, Falush D (2012) Inference of population structure using dense haplotype data. PLoS Genet 8: e1002453.

24. Patterson NJ, Moorjani P, Luo Y, Mallick S, Rohland N, Zhan Y, Genschoreck T, Webster T, Reich D (2012) Ancient admixture in Human history. Genetics 192: 1065-1093.

25. Reich D, Thangaraj K, Patterson N, Price AL, Singh L (2009) Reconstructing Indian population history. Nature 461: 489-494.

26. Peter BM (2016) Admixture, population structure and F-Statistics. Genetics Early online doi: 10.1534/genetics.115.183913

27. Boynton PJ, Greig D (2014) The ecology and evolution of non-domesticated *Saccharomyces* species. Yeast 31: 449-462.

28. Goddard MR, Greig D (2015) *Saccharomyces cerevisiae*: a nomadic yeast with no niche? FEMS Yeast Res 15.

29. Rodríguez ME, Pérez-Través L, Sangorrín MP, Barrio E, Lopes CA (2014) *Saccharomyces eubayanus* and *Saccharomyces uvarum* associated with the fermentation of *Araucaria araucana* seeds in Patagonia. FEMS Yeast Res 14: 948-965.

30. Libkind D, Hittinger CT, Valério E, Gonçalves C, Dover J, Johnston M, Gonçalves P, Sampaio JP (2011) Microbe domestication and the identification of the wild genetic stock of lager-brewing yeast. Proc Natl Acad Sci U S A 108: 14539-14544.

31. McCullough MJ, Clemons KV, McCusker JH, Stevens DA (1998) Intergenic transcribed spacer PCR ribotyping for differentiation of *Saccharomyces* species and interspecific hybrids. J Clin Microbiol 36: 1035-1038.

32. Rainieri S, Kodama Y, Kaneko Y, Mikata K, Nakao Y, Ashikari T (2006) Pure and mixed genetic lines of *Saccharomyces bayanus* and *Saccharomyces pastorianus* and their contribution to the lager brewing strain genome. Appl Environ Microbiol 72: 3968-3974.

33. Sampaio JP, Gonçalves P (2008) Natural populations of *Saccharomyces kudriavzevii* in Portugal are associated with oak bark and are sympatric with *S. cerevisiae* and *S. paradoxus*. Appl Environ Microbiol 74: 2144-2152.

34. Nguyen HV, Gaillardin C (2005) Evolutionary relationships between the former species *Saccharomyces uvarum* and the hybrids *Saccharomyces bayanus* and *Saccharomyces pastorianus*; reinstatement of *Saccharomyces uvarum* (Beijerinck) as a distinct species. FEMS Yeast Res 5: 471-483.

35. Belloch C, Orlic S, Barrio E, Querol A (2008) Fermentative stress adaptation of hybrids within the *Saccharomyces sensu stricto* complex. Int J Food Microbiol 122: 188-195.

36. Bing J, Han PJ, Liu WQ, Wang QM, Bai FY (2014) Evidence for a Far East Asian origin of lager beer yeast. Curr Biol 24: R380-R381.

37. Gayevskiy V, Goddard MR (2015) *Saccharomyces eubayanus* and *Saccharomyces arboricola* reside in North Island native New Zealand forests. Environ Microbiol 18: 1137-1147.

38. Almeida P, Gonçalves C, Teixeira S, Libkind D, Bontrager M, Masneuf-Pomarède I, Albertin W, Durrens P, Sherman DJ, Marullo P, Todd Hittinger C, Gonçalves P, Sampaio JP (2014) A Gondwanan imprint on global diversity and domestication of wine and cider yeast *Saccharomyces uvarum*. Nat Commun 5: 4044.

**Description of supporting information**

**S1 Table (Supplementary File S1 Table.xlsx).** Strains used in this study. Geographical, ecological, and gene/genome sequence information associated with *S. eubayanus* strains.

**S2 Table (Supplementary File S2 Table.xlsx).** Genes within the regions of interest.

**S3 Table (Supplementary File S4 Table.xlsx).** *f3*-, *D-*statistics and *f4*-ratio tests performed in ADMIXTOOLS.

**S4 Table.** Summary statistics for each population or group using multi-locus data.

**#seq #hap s k π Hd Tajima's D Fu & Li D Fu & Li F Fu's Fs**

**Holarctic Lineage**

Tibet 10 4 7 1.400 0.00018±0.00010 0.53 -1.8391* -2.1369* -2.3170* -0.1750

Lager 3 2 3 2.000 0.00026±0.00012 0.67 N.A. N.A. N.A. 1.6090

North Carolina 2 0 0 0.000 0.00000±0.00000 0.00 N.A. N.A. N.A. N.A.

**Subpopulation**

Holarctic 15 7 26 6.971 0.00090±0.00019 0.78 -0.53477 -0.08067 -0.23747 1.7660

Patagonia B 12 12 87 25.470 0.00328±0.00027 1.00 -0.58588 -0.69191 -0.75738 -2.1620

**Populations**

Patagonia B-Holarctic 28 20 130 28.899 0.00374±0.00136 0.94 -0.5499 -0.9334 -0.9532 0.2640

Patagonia A 7 7 72 34.381 0.00442±0.00071 1.00 0.9884 0.7028 0.8496 0.2510

West China 15 13 24 6.686 0.00086±0.00009 0.98 -0.3911 -0.2190 -0.3067 -5.0570

*p-value < 0.05

#seq: number of sequences; #hap: number of haplotypes; *k*: average number of differences between sequences; π: nucleotide diversity; Hd: Haplotype diversity.

**S5 Table (Supplementary File S5 Table.xlsx).** Summary of whole genome sequencing statistics.

**S6 Table.** PCR primer sequences and conditions used in the present study.

Gene Primer_name Strand Sequence(5'-3') Annealing

Tº

*FSY1*^a^ oHDP003 Forward GGATCYTCRACAAGCGTTTCTC 52ºC

*FSY1*^a^ oHDP004 Reverse AAGGCAAACAYGTAAAGCAAAG 52ºC

ITS5^b^ oHDP005 Forward GGAAGTAAAAGTCGTAACAAGG 52ºC

LR6^b^ oHDP006 Reverse CGCCAGTTCTGCTTACC 52ºC

ITS1^b^ oHDP007 Forward TCCGTAGGTGAACCTGCGG Sequencing

*FUN14*^c^ oHDP008 Forward TATTAAGCTGGGAGTGCCCTT 52ºC

*FUN14*^c^ oHDP009 Reverse TTATTGGCGTTTAGGCTTGA 52ºC

*RIP1*^c^ oHDP010 Forward AGATCGTCTGTTAATTCCTGC 50ºC

*RIP1*^c^ oHDP011 Reverse CCTTTTCACCTTCAAATTCG 50ºC

*MET2*^d^ oHDP012 Forward CGAAAACGCTCCAAGAGCTGG 55.5ºC

*MET2*^d^ oHDP013 Reverse GACCACGATATGCACCAGGCAG 55.5ºC

*GDH1*^e^ oHDP014 Forward TGGAAATGAGCGGAAGAAGAAAGC 55.5ºC

*GDH1*^f^ oHDP015 Reverse CTGTAGGCACCGAACAAGTAACC 55.5ºC

*HIS3*^c^ oHDP016 Forward ATGTCAGAGCAAAAGGCCCTA 55ºC

*HIS3*^c^ oHDP017 Reverse CATGAGAACACCCTTTGTGGA 55ºC

*COX2*^g^ oHDP018 Forward GGTATTTTAGAATTACATGA 45ºC

*COX2*^g^ oHDP019 Reverse ATTTATTGTTCRTTTAATCA 45ºC

*CCA1*^h^ oHDP119 Forward GCGATGAGGTTACCCTTG 49ºC

*CCA1*^h^ oHDP120 Reverse ATACTTGGCATAATGCTGCTG Sequencing

*CCA1*^h^ oHDP131 Reverse GGTGTGAGTTTAGTATGTTATCA 49ºC

*MLS1*^h^ oHDP121 Forward TTCTCCAAAGCGTGTCGTAG 49ºC

*MLS1*^h^ oHDP122 Reverse GTCCATGAAGGGGGAGGTCA 49ºC

*MLS1*^h^ oHDP132 Forward GCACAGGACTTTCAACGG 49ºC

*MLS1*^h^ oHDP133 Reverse ATGCTCTCAGTTTCAGGTAAG 49ºC

*PDR10*^h^ oHDP123 Forward ATTATGCCCACCGTGTCGTC 51ºC

*PDR10*^h^ oHDP124 Reverse ATACAGGGTTGACACCTTTGA Sequencing

*PDR10*^h^ oHDP134 Reverse CTTGCCACGGTGTATAAGGT 51ºC

*PDR10*^h^ oHDP135 Reverse TGGCAATCAGGTTCCAC Sequencing

Intergenic A-Y^h^ oHDP125 Forward CAAGAAGAAATTCCGATCACGACC 55ºC

Intergenic A-Y^h^ oHDP126 Reverse TCTGGCGATCCGAGATTGATTCC 55ºC

Intergenic F-R^h^ oHDP127 Forward CCAGTTCCCATGTGATTCTAT 50ºC

Intergenic F-R^h^ oHDP128 Reverse TCGTTCATCAGCACTTGCACTT 50ºC

Intergenic M-D^h^ oHDP129 Forward CAGTTTGGCTCAGATTTCATT 50ºC

Intergenic M-D^h^ oHDP130 Reverse GCGTGGGTTCCAGACTCATCC 50ºC

LTR_chrX^i^ oHDP144 Forward CGTGGAAAAGAAGGAAGAG 55ºC

LTR_chrX^i^ oHDP145 Reverse GGTGCAAGTAAGATGTAGTG 55ºC

^a^ [30]

^b^ [31]

^c^ [32]

^d^ [33]

^e^ [1]

^f^ [34]

^g^ [35]

^h^ [36]

^i^ This study

Annealing T = "Sequencing" are primers only used in the sequencing reaction.

**S1 Fig.** Distribution of host trees for *S. eubayanus* isolates.

A) Pie chart representing the tree genera from which *S. eubayanus* was isolated. The asterisk indicates the tree host for the 13 strains isolated by Rodríguez *et al.* [29]. B) Proportion of *S. eubayanus* associated to different tree orders. Populations were not designated by Rodríguez *et al.* [29], so these strains were excluded from Figure S1B. The P1C1 strain [37] lacks host information and it was not included in this figure.

**S2 Fig.** Multi-locus phylogenetic supernetwork summarizes cases of likely reticulation, including introgression, gene flow, and hybridization.

Phylogenetic supernetwork removing splits, excluding *PDR10* (a gene under balancing selection or reciprocal introgression) from the multi-locus dataset. Population assignment is represented by a blue, red, or brown shadow for Patagonia B-Holarctic, Patagonia A, or West China, respectively. The scale bar in the phylogenetic supernetwork represents the inferred edges’ weights using the average relative tree size option to normalize for different individual tree scales.

**S3 Fig.** Individual gene trees.

Cases of introgression or incomplete lineage sorting can be observed between Patagonia A and Patagonia B strains, such as yHCT96 (Patagonia A) whose *FUN14* allele is identical to the *FUN14* allele of several Patagonia B-Holarctic strains (S9C). Bootstrap values above 50 are reported to the left of their respective nodes. Scale bars represent nucleotide substitutions per site.

**S4 Fig.** Phylogenetic tree reconstruction of the regions of interest without collapsing the Patagonia A and Patagonia B strains.

Reconstruction of the phylogenetic tree of four of five regions of interest. These trees are identical to those shown in Fig 4 but the Patagonia A and Patagonia B clades were not collapsed. Bootstrap values above 50 are reported to the left of their respective nodes. Scale bars represent nucleotide substitutions per site.

**S5 Fig.** Genome-wide log_2_ ratios of pairwise divergence of the Saaz lager representative to key populations and lineages.

A) Tibet-Saaz versus North Carolina-Saaz, B) Tibet-Saaz versus Patagonia B-Saaz, and C) North Carolina-Saaz versus Patagonia B-Saaz. Arrows indicate the direction where log_2_ ratios of pairwise divergence suggest a relatively closer relationship to a particular lineage or population. The Patagonia B value reported is the lowest pairwise divergence value of all Patagonia B strains for that window. The window size is 50-kbp.

**S6 Fig.** Genome-wide log_2_ ratios of pairwise divergence of the Frohberg lager representative to key populations or lineages.

A) Tibet-Frohberg versus North Carolina-Frohberg, B) Tibet-Frohberg versus Patagonia B-Frohberg, and C) North Carolina-Frohberg versus Patagonia B-Frohberg. Arrows indicate the direction where log_2_ ratios of pairwise divergence suggest a relatively closer relationship to a particular lineage or population. The Patagonia B value reported is the lowest pairwise divergence value of all Patagonia B strains for that window. The window size is 50-kbp.

**S7 Fig.** Region of interest on chromosome X.

A) Alignment of the region of interest on chromosome X. Genes annotated in this region are represented above the alignment. Black lines represents nucleotide differences compared with the reference sequence of FM1318. Gaps are represented as white spaces; gaps in FM1318 or CBS7001 are gaps in the alignment, rather than gaps in the assemblies. B) and C) are ML phylogenetic trees reconstructed using the segments of chromosome X region indicated by the light blue and dark blue colors, respectively. Bootstrap values above 50 are reported to the left of their respective nodes. Scale bars represent nucleotide substitutions per site.

**S8 Fig.** Copy number variation, heterozygosity levels, and gene annotations of the regions of interest for the Frohberg and Saaz representatives.

Copy number graphs of chromosomes III, IV, VIII, X, and XI for the regions of interest for the Saaz (CBS1503) and Frohberg (W34/70) representatives. These graphs were extracted from the complete chromosome representations in S9 Fig. The coordinates correspond to the FM1318 reference genome. The lower panels correspond only to the regions demarcated by the dashed lines in the upper panels. The lower panels report the coverage values (using 1-kbp windows) for the regions of interest, gene annotations, and the absolute counts of homozygous and heterozygous SNPs (using 1-kbp windows) compared with the FM1318 reference genome.

**S9 Fig.** Copy number and heterozygosity levels of *S. eubayanus* and Lager strains.

Coverage levels normalized using the median value of coverage for the complete genome are shown for the *S. eubayanus* subgenome in the Saaz (CBS1503) and Frohberg (W34/70) in A) and B). Normalized coverage levels for non-hybrid strains of *S. eubayanus* are shown in C) to J). The chromosome copy numbers of hybrids were inferred by establishing the lowest average coverage values for one copy (i.e. chromosome II of the Saaz, CBS1503, and chromosome I of the Frohberg, W34/70). Absolute counts of homozygous and heterozygous SNPs (using 50-kbp windows) compared with the FM1318 reference genome are shown in the bottom graph for each strain. High levels of heterozygosity were detected in subtelomeric regions and a handful of other regions outside of the regions of interest (S9 Fig). These regions of high heterozygosity were shared among strains, including the monosporic and homozygous strain FM1318 (panel J), suggesting they were false positives. The regions of interest (S8 Fig) have less heterozygosity (1.08*10^-4^ and 8.49*10^-5^ heterozygous site/bp for Saaz and Frohberg, respectively) than the average heterozygosity detected genome-wide (2.08*10^-4^ and 4.86*10^-4^ heterozygous site/bp for Saaz and Frohberg, respectively). Moreover, heterozygosity was not positively correlated with an increase in the number of copies inferred (linear regression r^2^=0.097, p-value=0.381). Nucleotide diversity levels of the annotated genes within the regions of interest (S8 Fig, S2 Table) were, in general, lower than the average value found genome-wide among the strains from the Patagonia A-Patagonia B-Holarctic clade (0.57%). For 14 of 44 genes the values were higher but less than twice the genome-wide diversity values. Based on comparisons to the multi-locus dataset, the false positive rate of our pipeline at calling non-heterozygous sites was low (4.63*10^-5^SNPs/site) and not sufficient to influence conclusions regarding the regions of interest.

**S10 Fig.** *S. eubayanus* and *S. uvarum* phylogenetic tree comparison.

*S. eubayanus* and *S. uvarum* phylogenetic trees are shown in A) and B), respectively. Color bars represent populations for each species, and are colored according to the colors used in the previous *S. eubayanus* phylogenetic tree figures. Demographically similar populations of *S. uvarum* use the analogous colors from *S. eubayanus*. The multi-locus *S. eubayanus* phylogenetic tree is from Fig 1B, while the *S. uvarum* phylogenetic tree is reconstructed from Almeida *et al.* [38] after correcting branch lengths for the presence of invariant sites. Phylogenetic trees were rooted using *S. uvarum* (CBS7001) or *S. eubayanus* (FM1318) as the outgroup in A) and B), respectively. The scale bar represents the number of substitutions per site.

**S11 Fig.** Time-calibrated phylogenetic tree.

Blue, red, and brown bars indicate the population designation for Patagonia B-Holarctic, Patagonia A, and West China, respectively. The scale bar represents divergence time in thousands of years (kya).

**S12 Fig.** The recombinant TTH27L.1 *MLS1* gene sequence is likely an artifact.

The TTH27L.1 *MLS1* sequence reported in GenBank appeared to be a recombinant version between *S. eubayanus* West China and *S. uvarum*. Black and gray colors represent polymorphisms from *S. uvarum* and *S. eubayanus* West China, respectively. The phylogenetic trees in S2 Fig of Bing *et al.* [36] suggested that the TTH27L.1 and PYCC 6148^T^ (=CRUB 1568^T^) *MLS1* sequences were not recombinant; however, the sequences deposited in GenBank (KF892364 and KF892348, respectively) appeared to be recombinant. Our copy of the strain PYCC 6148^T^ did not possess a recombinant *MLS1*, but we could not check the strain TTH27L.1 because it is not available for study. We noted that the apparent recombination point for both strains is at the junction of the promoter and coding sequence, so we suspect that errors were introduced *in silico* while the sequences were uploaded to GenBank or when multiple Sanger sequencing reads were assembled. Absent further direct verification of TTH27L.1 *MLS1*, we suggest that the apparent recombination is likely an artifact.
