# Supplementary material for: Complex Ancestries of Lager-Brewing Hybrids Were Shaped by Standing Variation in the Wild Yeast Saccharomyces eubayanus
Source: PLoS Genet. 2016 Jul 6;12(7):e1006155. doi: 10.1371/journal.pgen.1006155 (PMC4934787; doi:10.1371/journal.pgen.1006155)
Supplement: S4 Table — (DOCX) [file pgen.1006155.s005.docx]

**S4 Table.** Summary statistics for each population or group using multi-locus data.

**#seq #hap s k π Hd Tajima's D Fu & Li D Fu & Li F Fu's Fs**

**Holarctic Lineage**

Tibet 10 4 7 1.400 0.00018±0.00010 0.53 -1.8391* -2.1369* -2.3170* -0.1750

Lager 3 2 3 2.000 0.00026±0.00012 0.67 N.A. N.A. N.A. 1.6090

North Carolina 2 0 0 0.000 0.00000±0.00000 0.00 N.A. N.A. N.A. N.A.

**Subpopulation**

Holarctic 15 7 26 6.971 0.00090±0.00019 0.78 -0.53477 -0.08067 -0.23747 1.7660

Patagonia B 12 12 87 25.470 0.00328±0.00027 1.00 -0.58588 -0.69191 -0.75738 -2.1620

**Populations**

Patagonia B-Holarctic 28 20 130 28.899 0.00374±0.00136 0.94 -0.5499 -0.9334 -0.9532 0.2640

Patagonia A 7 7 72 34.381 0.00442±0.00071 1.00 0.9884 0.7028 0.8496 0.2510

West China 15 13 24 6.686 0.00086±0.00009 0.98 -0.3911 -0.2190 -0.3067 -5.0570

*p-value < 0.05

#seq: number of sequences; #hap: number of haplotypes; *k*: average number of differences between sequences; π: nucleotide diversity; Hd: Haplotype diversity.
