# Supplementary material for: Complex Ancestries of Lager-Brewing Hybrids Were Shaped by Standing Variation in the Wild Yeast Saccharomyces eubayanus
Source: PLoS Genet. 2016 Jul 6;12(7):e1006155. doi: 10.1371/journal.pgen.1006155 (PMC4934787; doi:10.1371/journal.pgen.1006155)
Supplement: S6 Table — (DOCX) [file pgen.1006155.s007.docx]

**S6 Table.** PCR primer sequences and conditions used in the present study.

Gene Primer_name Strand Sequence(5'-3') Annealing

Tº

*FSY1*^a^ oHDP003 Forward GGATCYTCRACAAGCGTTTCTC 52ºC

*FSY1*^a^ oHDP004 Reverse AAGGCAAACAYGTAAAGCAAAG 52ºC

ITS5^b^ oHDP005 Forward GGAAGTAAAAGTCGTAACAAGG 52ºC

LR6^b^ oHDP006 Reverse CGCCAGTTCTGCTTACC 52ºC

ITS1^b^ oHDP007 Forward TCCGTAGGTGAACCTGCGG Sequencing

*FUN14*^c^ oHDP008 Forward TATTAAGCTGGGAGTGCCCTT 52ºC

*FUN14*^c^ oHDP009 Reverse TTATTGGCGTTTAGGCTTGA 52ºC

*RIP1*^c^ oHDP010 Forward AGATCGTCTGTTAATTCCTGC 50ºC

*RIP1*^c^ oHDP011 Reverse CCTTTTCACCTTCAAATTCG 50ºC

*MET2*^d^ oHDP012 Forward CGAAAACGCTCCAAGAGCTGG 55.5ºC

*MET2*^d^ oHDP013 Reverse GACCACGATATGCACCAGGCAG 55.5ºC

*GDH1*^e^ oHDP014 Forward TGGAAATGAGCGGAAGAAGAAAGC 55.5ºC

*GDH1*^f^ oHDP015 Reverse CTGTAGGCACCGAACAAGTAACC 55.5ºC

*HIS3*^c^ oHDP016 Forward ATGTCAGAGCAAAAGGCCCTA 55ºC

*HIS3*^c^ oHDP017 Reverse CATGAGAACACCCTTTGTGGA 55ºC

*COX2*^g^ oHDP018 Forward GGTATTTTAGAATTACATGA 45ºC

*COX2*^g^ oHDP019 Reverse ATTTATTGTTCRTTTAATCA 45ºC

*CCA1*^h^ oHDP119 Forward GCGATGAGGTTACCCTTG 49ºC

*CCA1*^h^ oHDP120 Reverse ATACTTGGCATAATGCTGCTG Sequencing

*CCA1*^h^ oHDP131 Reverse GGTGTGAGTTTAGTATGTTATCA 49ºC

*MLS1*^h^ oHDP121 Forward TTCTCCAAAGCGTGTCGTAG 49ºC

*MLS1*^h^ oHDP122 Reverse GTCCATGAAGGGGGAGGTCA 49ºC

*MLS1*^h^ oHDP132 Forward GCACAGGACTTTCAACGG 49ºC

*MLS1*^h^ oHDP133 Reverse ATGCTCTCAGTTTCAGGTAAG 49ºC

*PDR10*^h^ oHDP123 Forward ATTATGCCCACCGTGTCGTC 51ºC

*PDR10*^h^ oHDP124 Reverse ATACAGGGTTGACACCTTTGA Sequencing

*PDR10*^h^ oHDP134 Reverse CTTGCCACGGTGTATAAGGT 51ºC

*PDR10*^h^ oHDP135 Reverse TGGCAATCAGGTTCCAC Sequencing

Intergenic A-Y^h^ oHDP125 Forward CAAGAAGAAATTCCGATCACGACC 55ºC

Intergenic A-Y^h^ oHDP126 Reverse TCTGGCGATCCGAGATTGATTCC 55ºC

Intergenic F-R^h^ oHDP127 Forward CCAGTTCCCATGTGATTCTAT 50ºC

Intergenic F-R^h^ oHDP128 Reverse TCGTTCATCAGCACTTGCACTT 50ºC

Intergenic M-D^h^ oHDP129 Forward CAGTTTGGCTCAGATTTCATT 50ºC

Intergenic M-D^h^ oHDP130 Reverse GCGTGGGTTCCAGACTCATCC 50ºC

LTR_chrX^i^ oHDP144 Forward CGTGGAAAAGAAGGAAGAG 55ºC

LTR_chrX^i^ oHDP145 Reverse GGTGCAAGTAAGATGTAGTG 55ºC

^a^ [30]

^b^ [31]

^c^ [32]

^d^ [33]

^e^ [1]

^f^ [34]

^g^ [35]

^h^ [36]

^i^ This study

Annealing T = "Sequencing" are primers only used in the sequencing reaction.
