# Supplementary figures and images for: Complex Ancestries of Lager-Brewing Hybrids Were Shaped by Standing Variation in the Wild Yeast Saccharomyces eubayanus
Source: PLoS Genet. 2016 Jul 6;12(7):e1006155. doi: 10.1371/journal.pgen.1006155 (PMC4934787; doi:10.1371/journal.pgen.1006155)

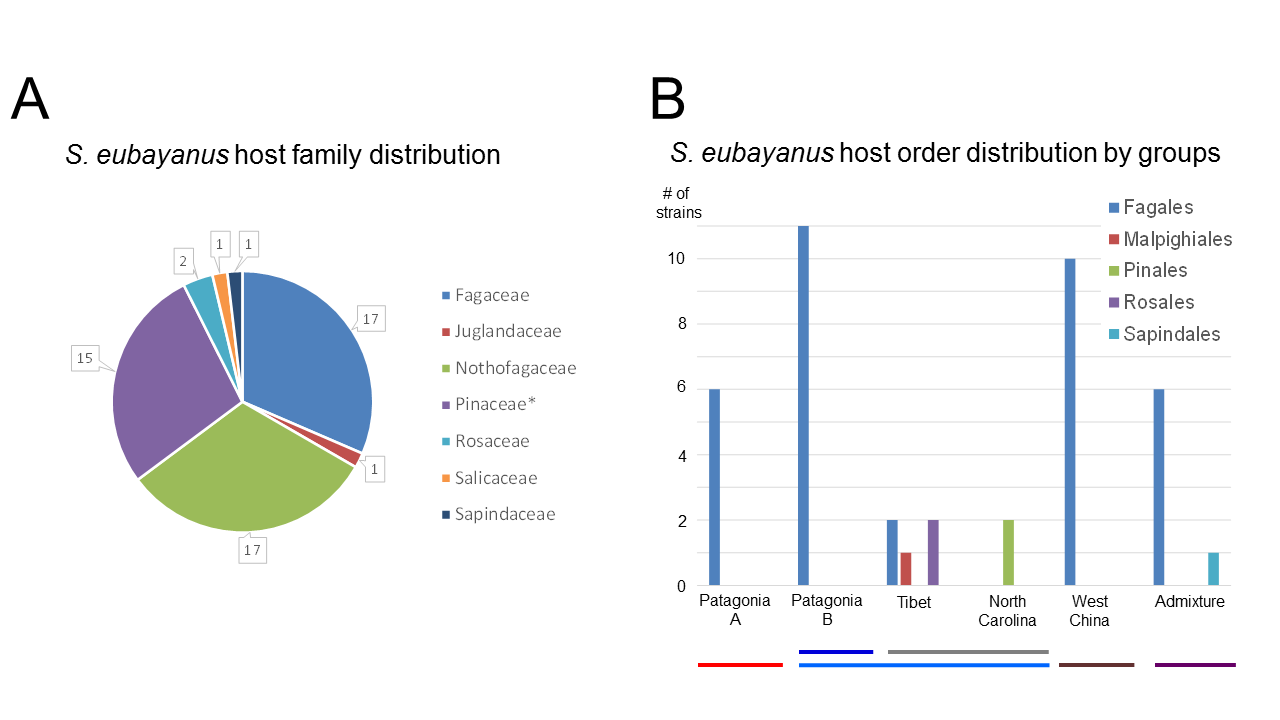

Supplement: S1 Fig — A) Pie chart representing the tree genera from which S. eubayanus was isolated. The asterisk indicates the tree host for the 13 strains isolated by Rodríguez et al. [24]. B) Proportion of S. eubayanus associated to different tree orders. Populations were not designated by Rodríguez et al. [24], so these strains were excluded from S1B Fig. The P1C1 strain [22] lacks host information and it was not included in this figure. (TIF) [file pgen.1006155.s008.tif]

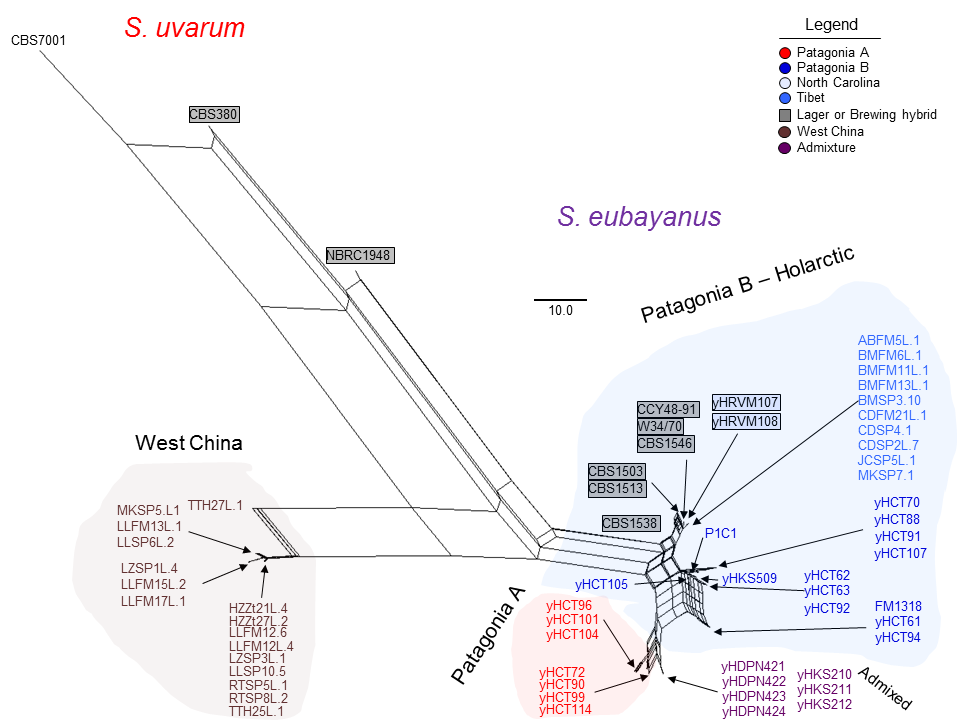

Supplement: S2 Fig — Phylogenetic supernetwork removing splits, excluding PDR10 (a gene under balancing selection or reciprocal introgression) from the multi-locus dataset. Population assignment is represented by a blue, red, or brown shadow for Patagonia B-Holarctic, Patagonia A, or West China, respectively. The scale bar in the phylogenetic supernetwork represents the inferred edges’ weights using the average relative tree size option to normalize for different individual tree scales. (TIF) [file pgen.1006155.s009.tif]

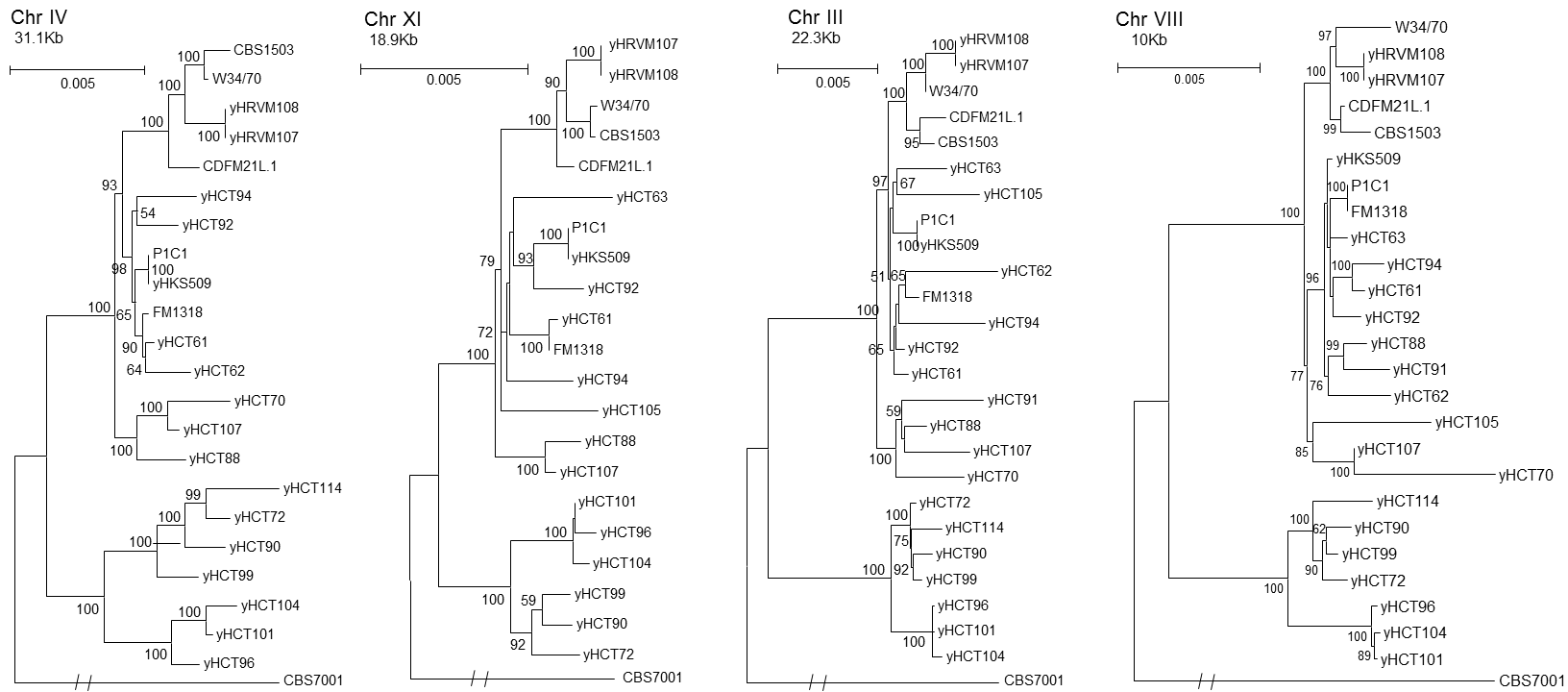

Supplement: S4 Fig — Reconstruction of the phylogenetic tree of four of five regions of interest. These trees are identical to those shown in Fig 4 but the Patagonia A and Patagonia B clades were not collapsed. Bootstrap values above 50 are reported to the left of their respective nodes. Scale bars represent nucleotide substitutions per site. (TIF) [file pgen.1006155.s011.tif]

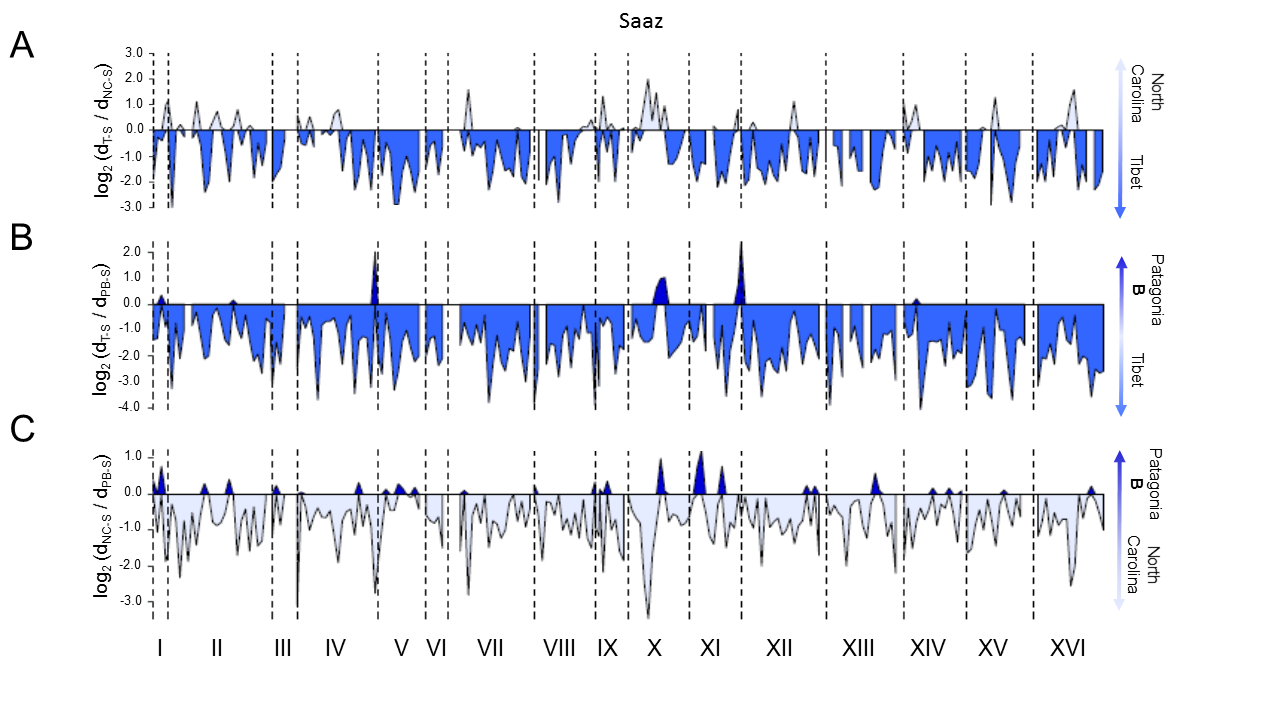

Supplement: S5 Fig — A) Tibet-Saaz versus North Carolina-Saaz, B) Tibet-Saaz versus Patagonia B-Saaz, and C) North Carolina-Saaz versus Patagonia B-Saaz. Arrows indicate the direction where log2 ratios of pairwise divergence suggest a relatively closer relationship to a particular lineage or population. The Patagonia B value reported is the lowest pairwise divergence value of all Patagonia B strains for that window. The window size is 50-kbp. (TIF) [file pgen.1006155.s012.tif]

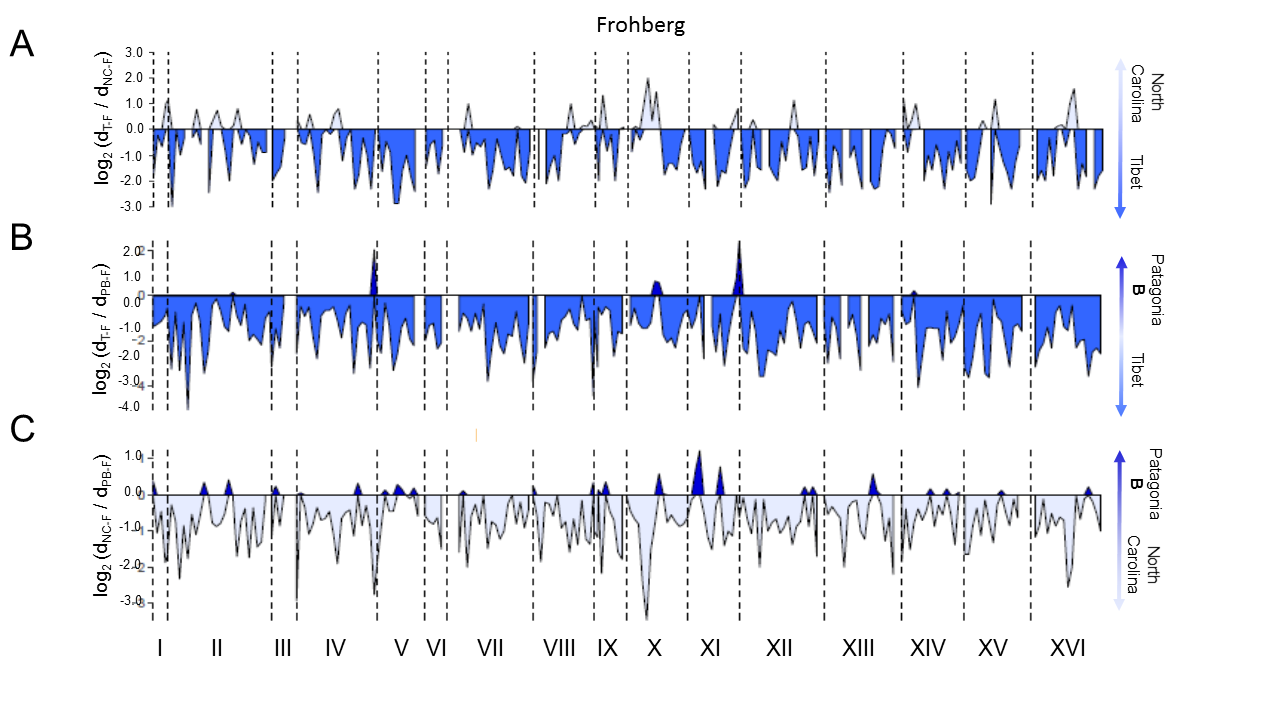

Supplement: S6 Fig — A) Tibet-Frohberg versus North Carolina-Frohberg, B) Tibet-Frohberg versus Patagonia B-Frohberg, and C) North Carolina-Frohberg versus Patagonia B-Frohberg. Arrows indicate the direction where log2 ratios of pairwise divergence suggest a relatively closer relationship to a particular lineage or population. The Patagonia B value reported is the lowest pairwise divergence value of all Patagonia B strains for that window. The window size is 50-kbp. (TIF) [file pgen.1006155.s013.tif]

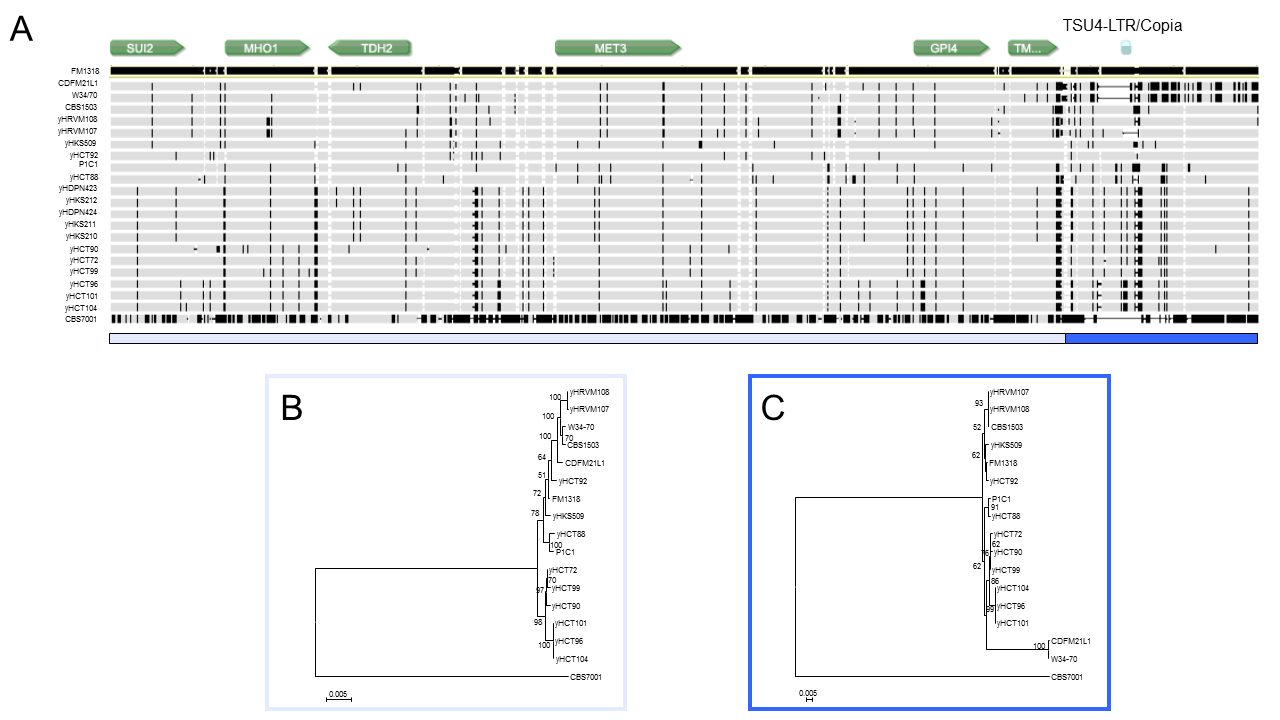

Supplement: S7 Fig — A) Alignment of the region of interest on chromosome X. Genes annotated in this region are represented above the alignment. Black lines represents nucleotide differences compared with the reference sequence of FM1318. Gaps are represented as white spaces; gaps in FM1318 or CBS7001 are gaps in the alignment, rather than gaps in the assemblies. B) and C) are ML phylogenetic trees reconstructed using the segments of chromosome X region indicated by the light blue and dark blue colors, respectively. Bootstrap values above 50 are reported to the left of their respective nodes. Scale bars represent nucleotide substitutions per site. (TIF) [file pgen.1006155.s014.tif]

Chromosome III

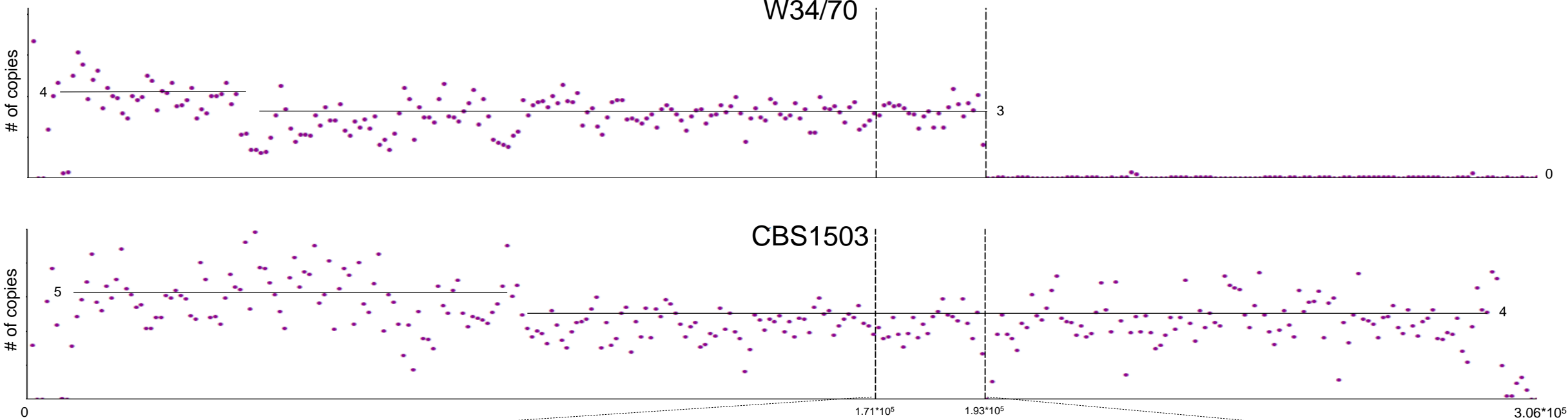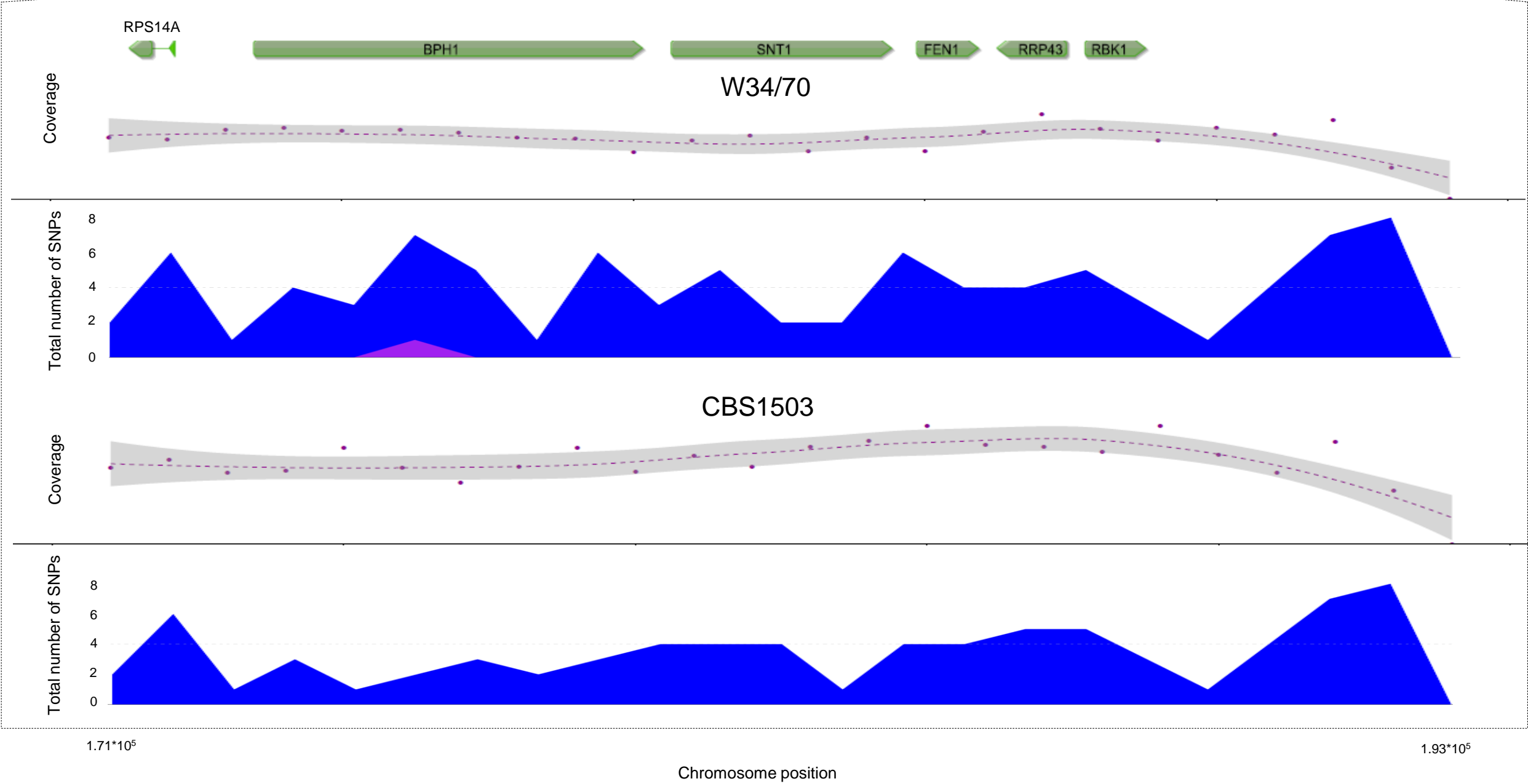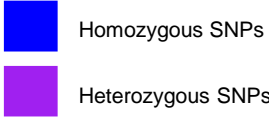

Chromosome IV

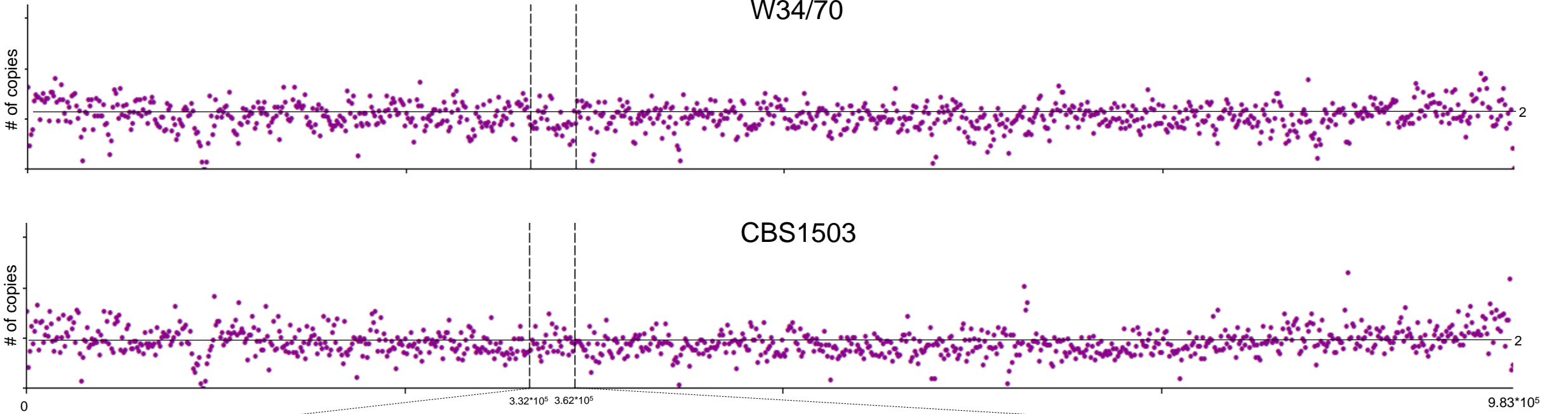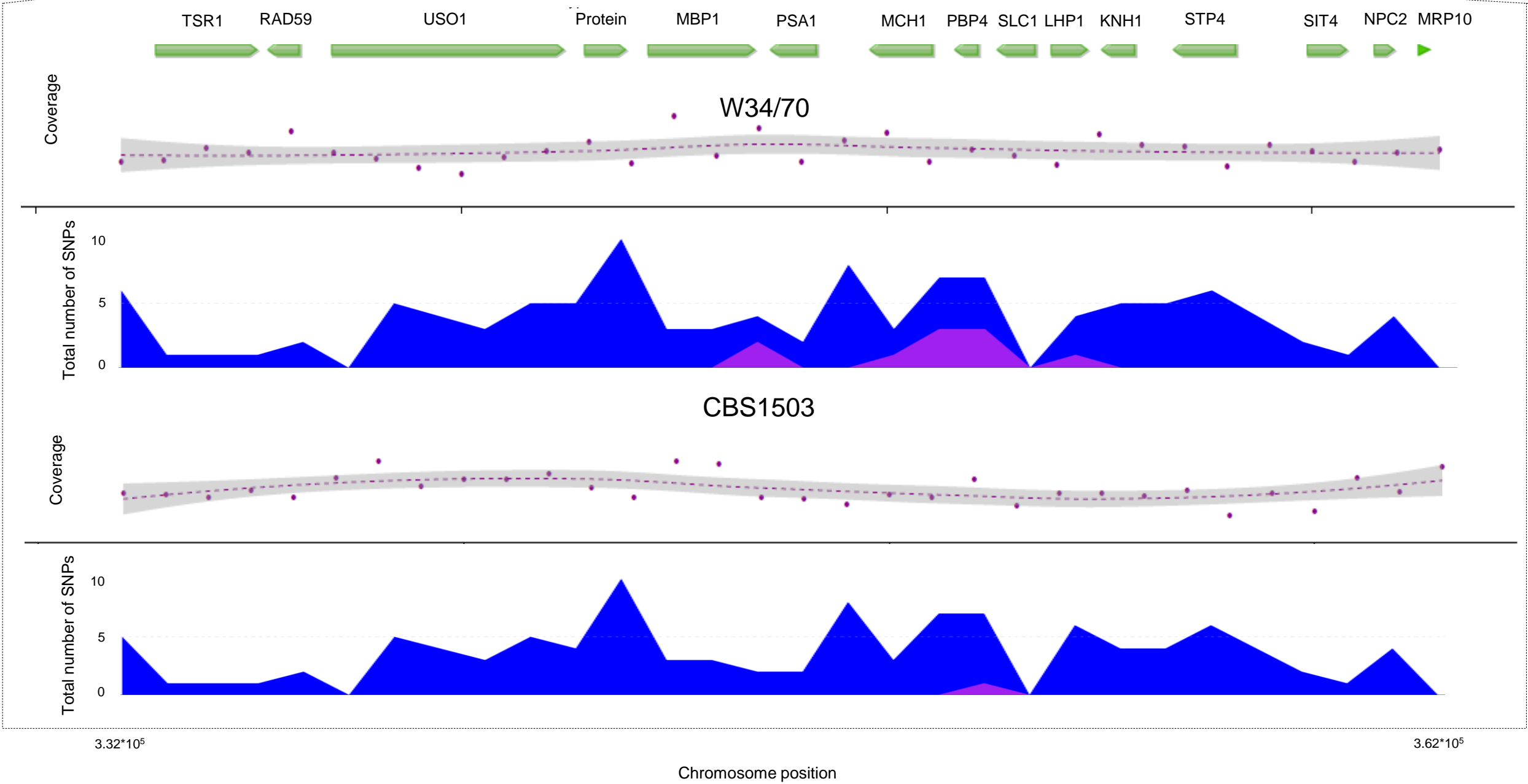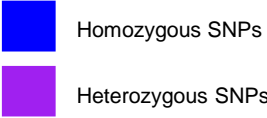

Chromosome VIII

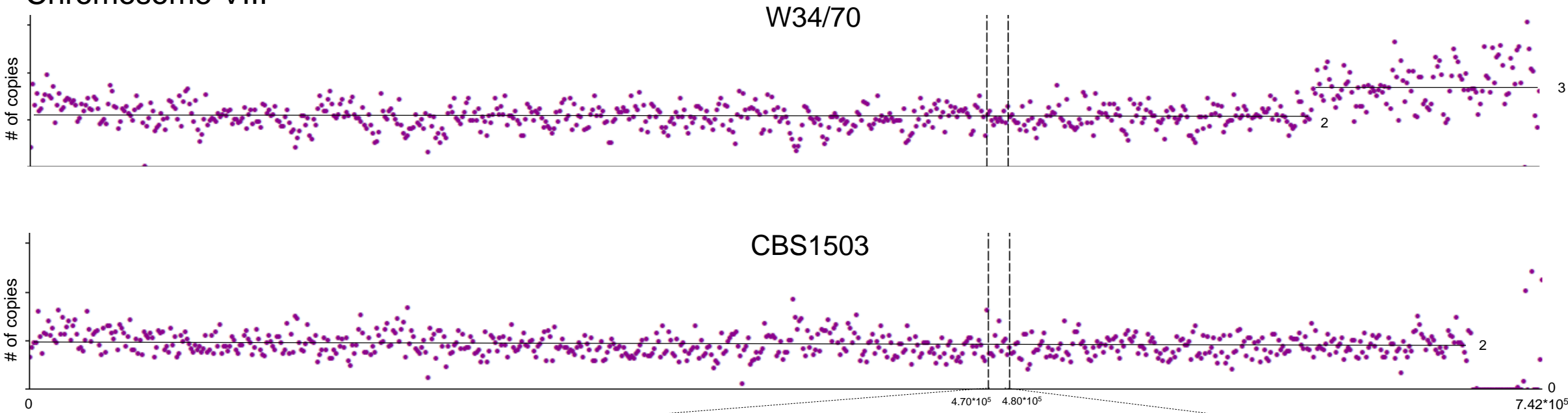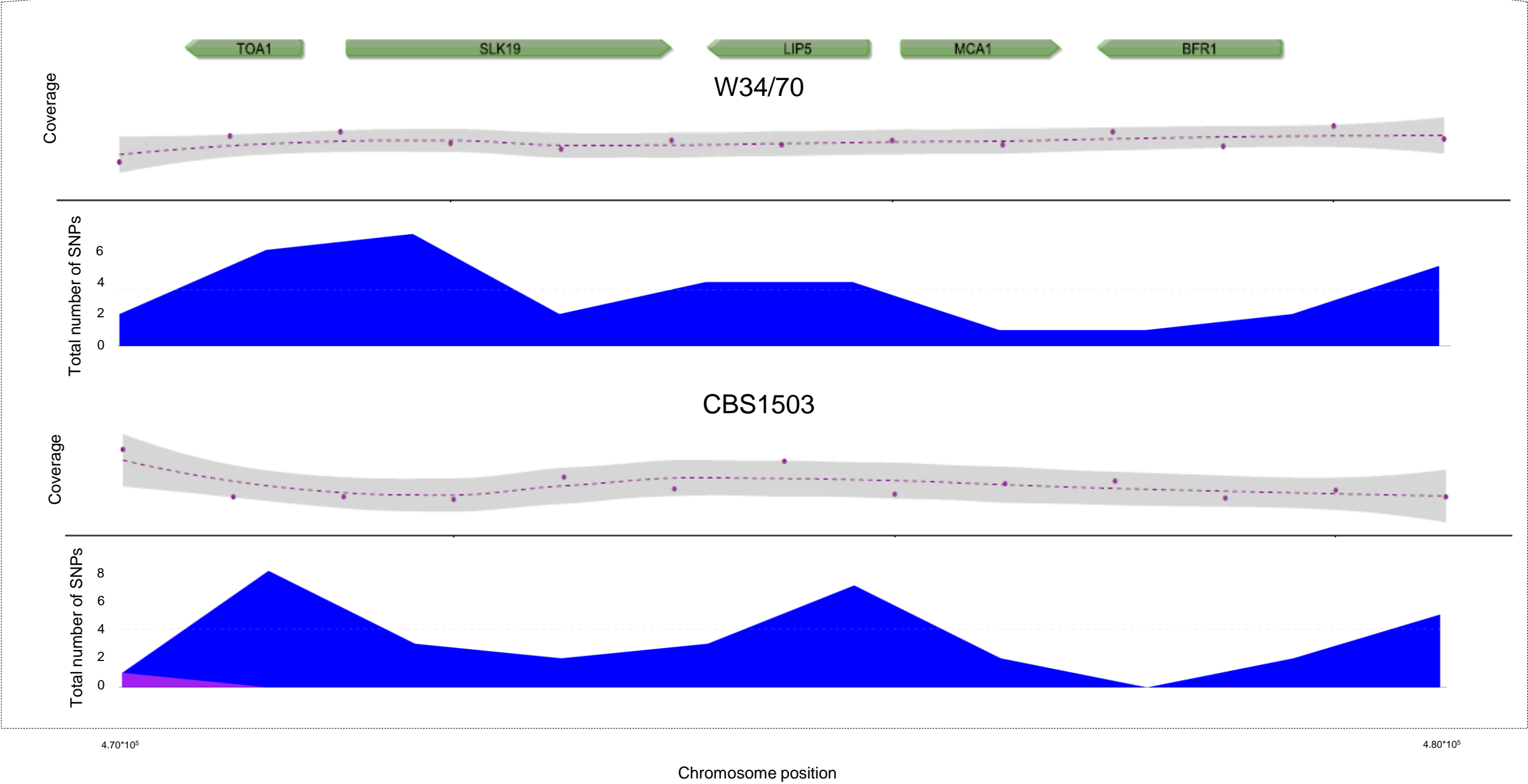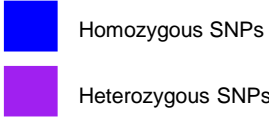

Chromosome X

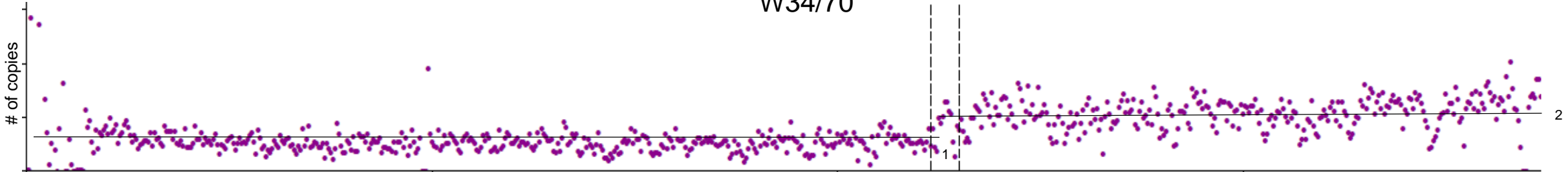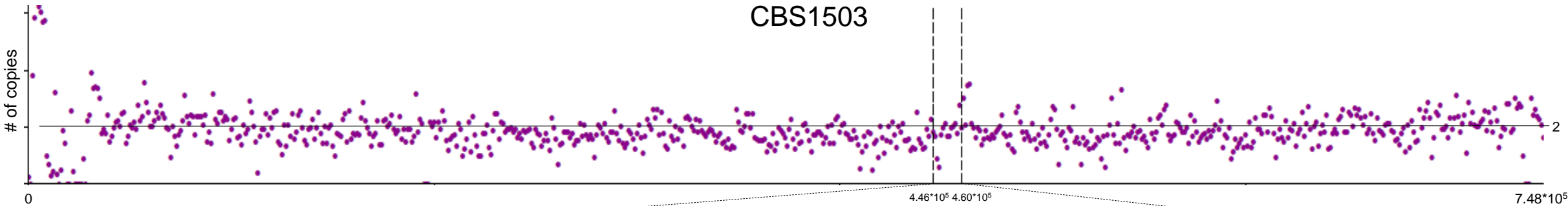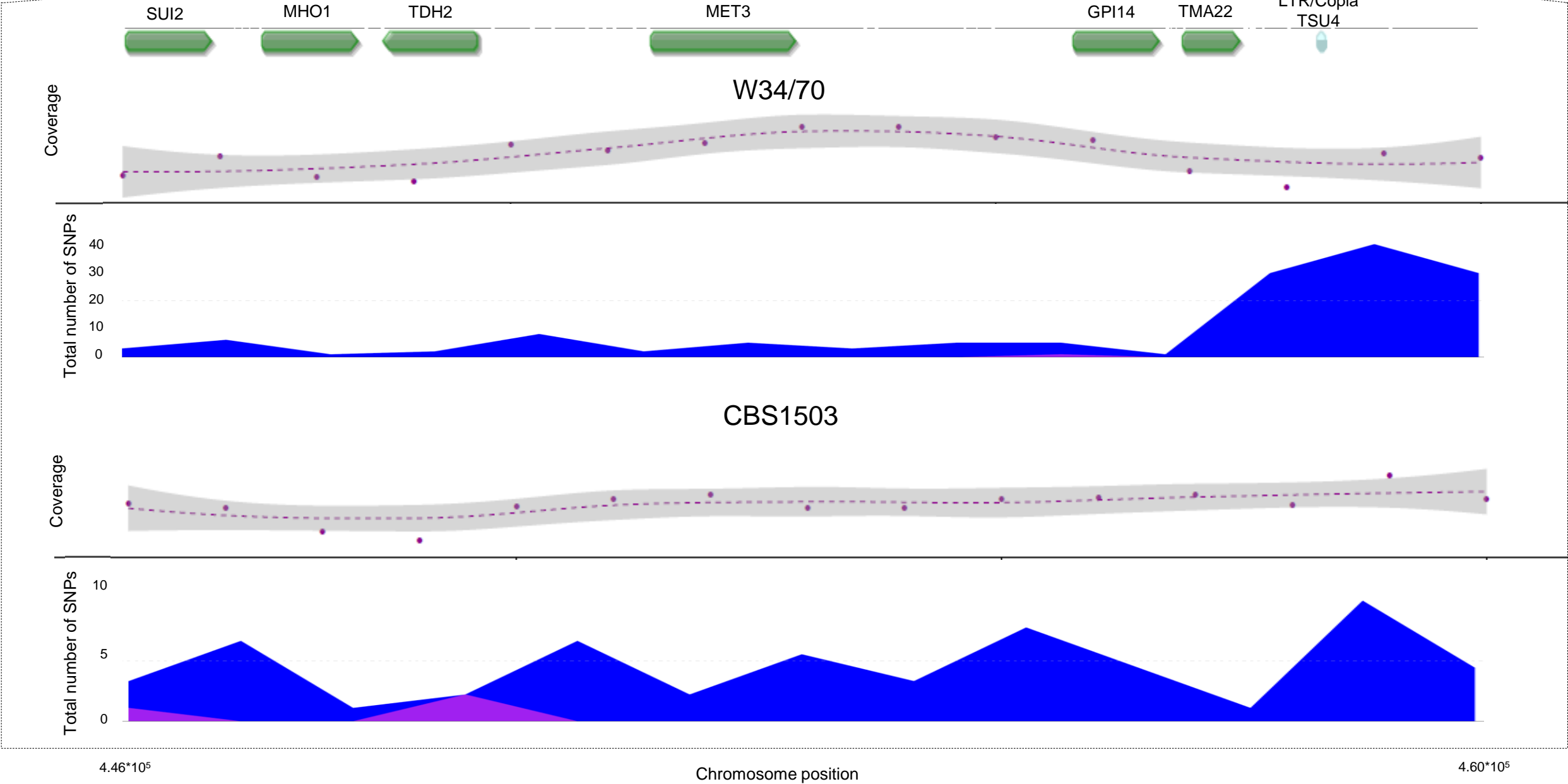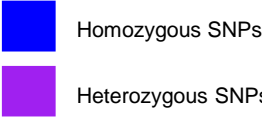

Chromosome XI

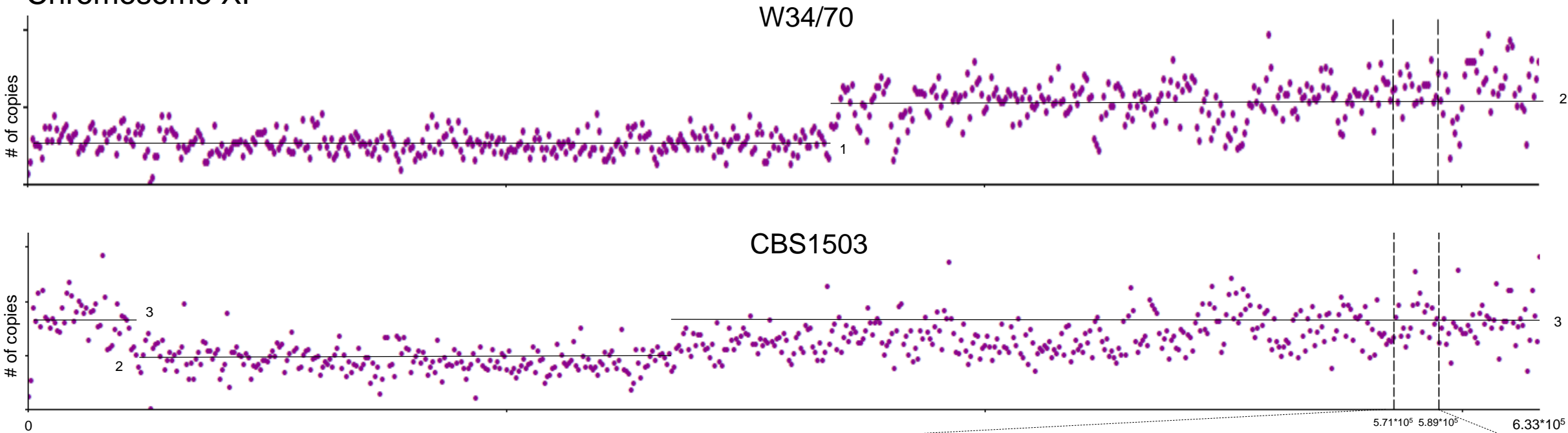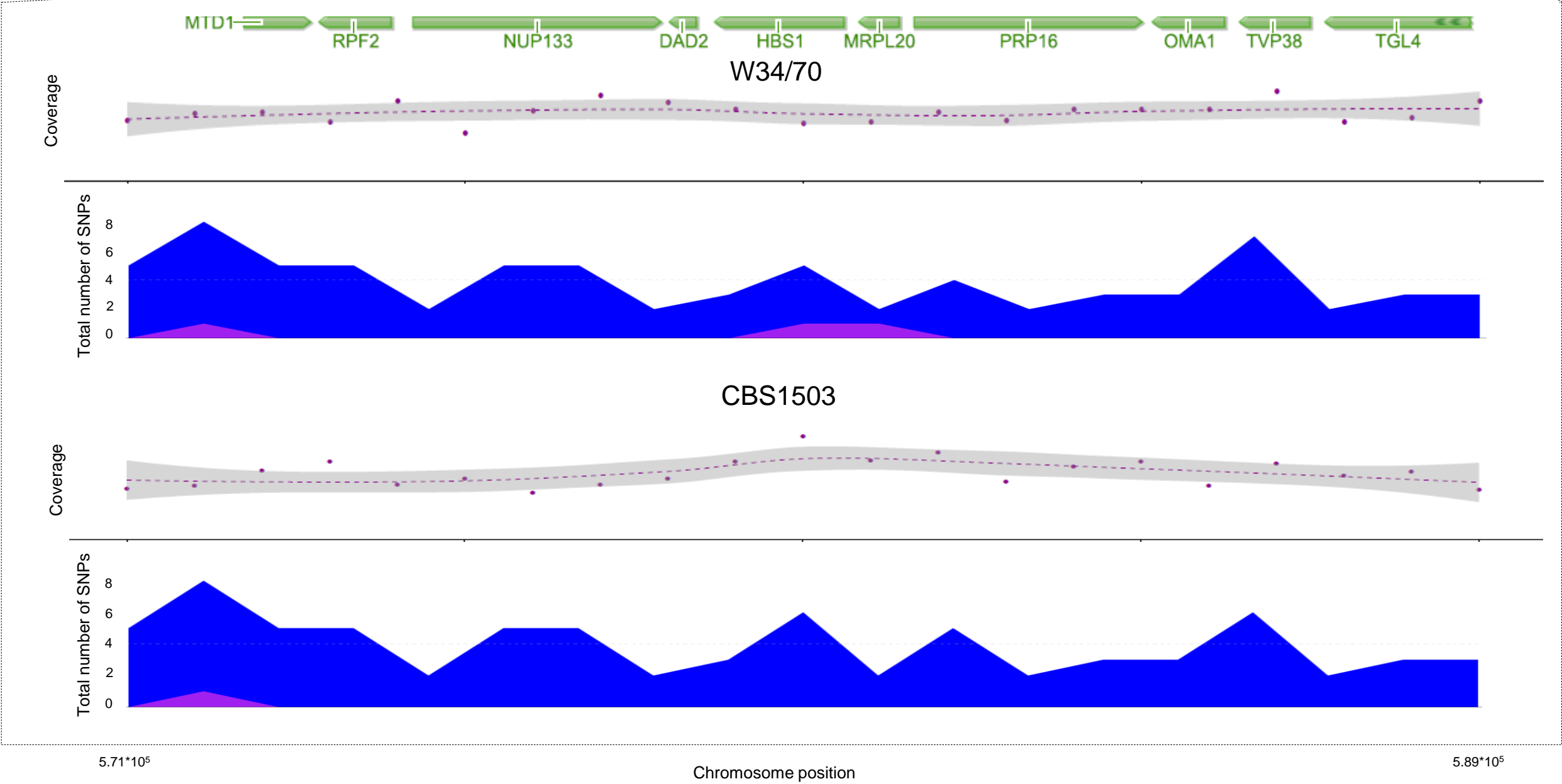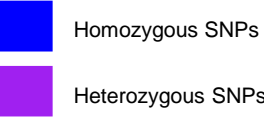

Supplement: S8 Fig — Copy number graphs of chromosomes III, IV, VIII, X, and XI for the regions of interest for the Saaz (CBS1503) and Frohberg (W34/70) representatives. These graphs were extracted from the complete chromosome representations in S9 Fig. The coordinates correspond to the FM1318 reference genome. The lower panels correspond only to the regions demarcated by the dashed lines in the upper panels. The lower panels report the coverage values (using 1-kbp windows) for the regions of interest, gene annotations, and the absolute counts of homozygous and heterozygous SNPs (using 1-kbp windows) compared with the FM1318 reference genome. (PDF) [file pgen.1006155.s015.pdf]

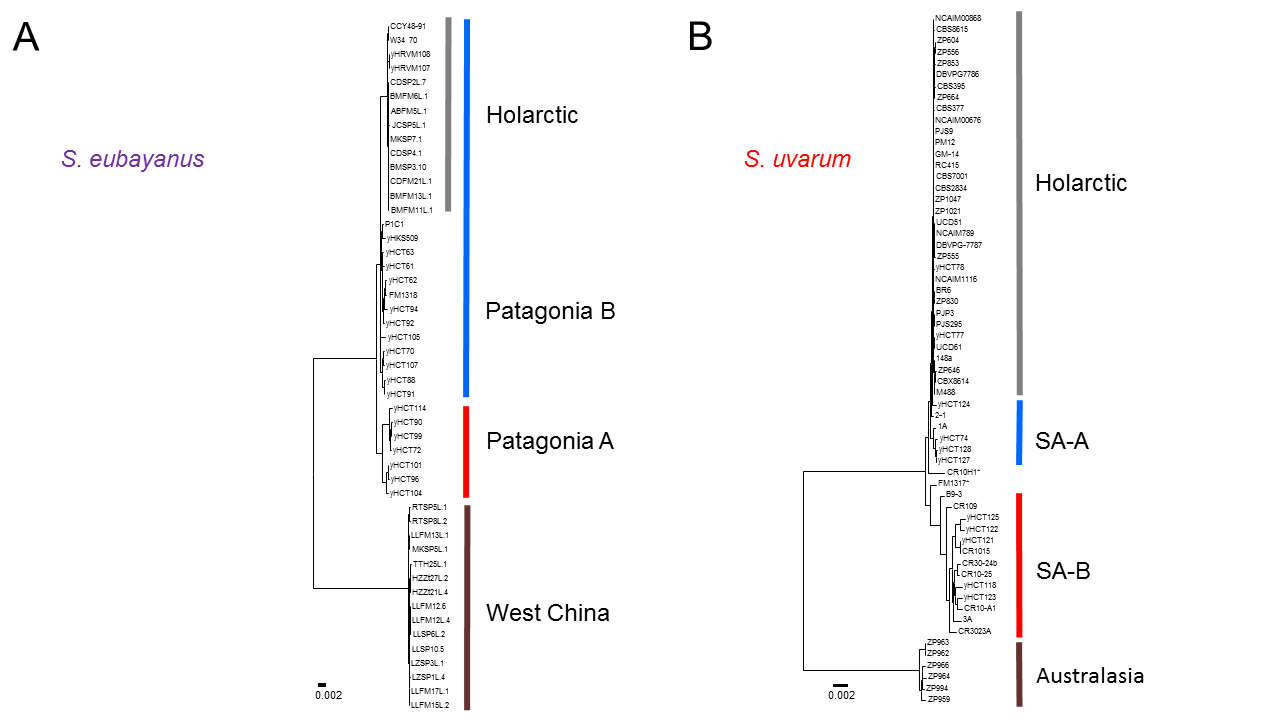

Supplement: S10 Fig — S. eubayanus and S. uvarum phylogenetic trees are shown in A) and B), respectively. Color bars represent populations for each species, and are colored according to the colors used in the previous S. eubayanus phylogenetic tree figures. Demographically similar populations of S. uvarum use the analogous colors from S. eubayanus. The multi-locus S. eubayanus phylogenetic tree is from Fig 1B, while the S. uvarum phylogenetic tree is reconstructed from Almeida et al. [23] after correcting branch lengths for the presence of invariant sites. Phylogenetic trees were rooted using S. uvarum (CBS7001) or S. eubayanus (FM1318) as the outgroup in A) and B), respectively. The scale bar represents the number of substitutions per site. (TIF) [file pgen.1006155.s017.TIF]

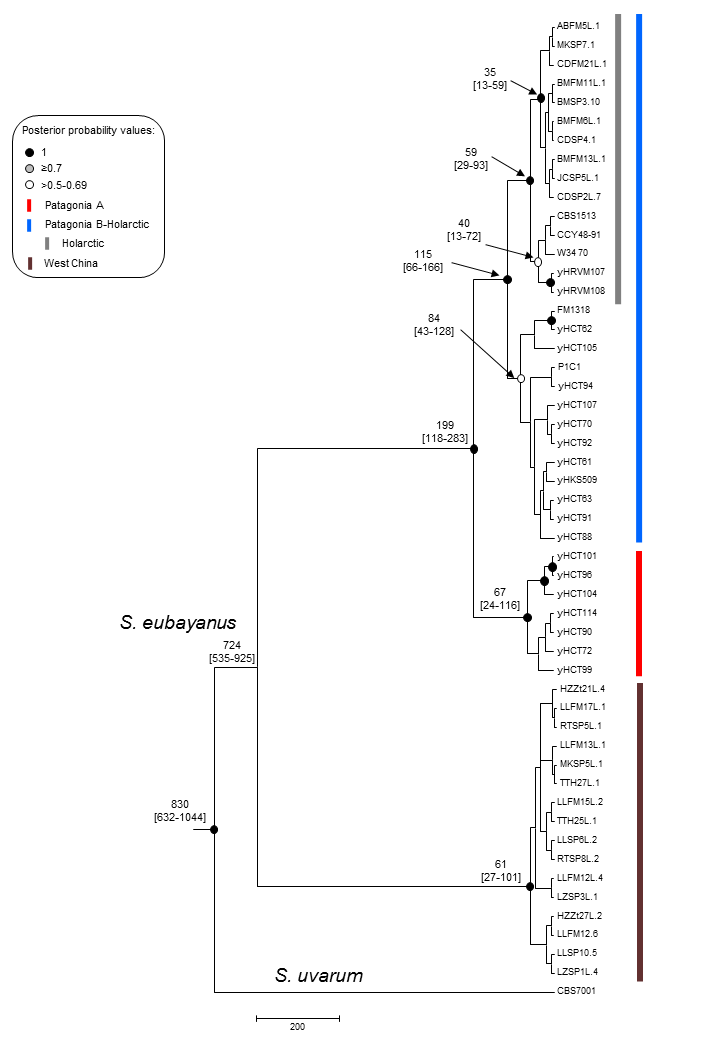

Supplement: S11 Fig — Blue, red, and brown bars indicate the population designation for Patagonia B-Holarctic, Patagonia A, and West China, respectively. The scale bar represents divergence time in thousands of years (kya). (TIF) [file pgen.1006155.s018.TIF]
