# Supplementary material for: Complex Ancestries of Lager-Brewing Hybrids Were Shaped by Standing Variation in the Wild Yeast Saccharomyces eubayanus
Source: PLoS Genet. 2016 Jul 6;12(7):e1006155. doi: 10.1371/journal.pgen.1006155 (PMC4934787; doi:10.1371/journal.pgen.1006155)
Supplement: S3 Fig — Each panel represent the phylogenetic tree reconstructed using A) CCA1, B) FSY1, C) FUN14, D) GDH1, E) HIS3, F) Intergenic region between APP1 and YPT53, G) Intergenic region between FAR8 and RSF1, H) Intergenic region between MSL1 and DSN1, I) MET2, J) MSL1, K) PDR10, L) RIP1, and M) COX2 sequence. Cases of introgression or incomplete lineage sorting can be observed between Patagonia A and Patagonia B strains, such as yHCT96 (Patagonia A) whose FUN14 allele is identical to the FUN14 allele of several Patagonia B-Holarctic strains (S9C Fig). Bootstrap values above 50 are reported to the left of their respective nodes. Scale bars represent nucleotide substitutions per site. (PDF) [file pgen.1006155.s010.pdf]

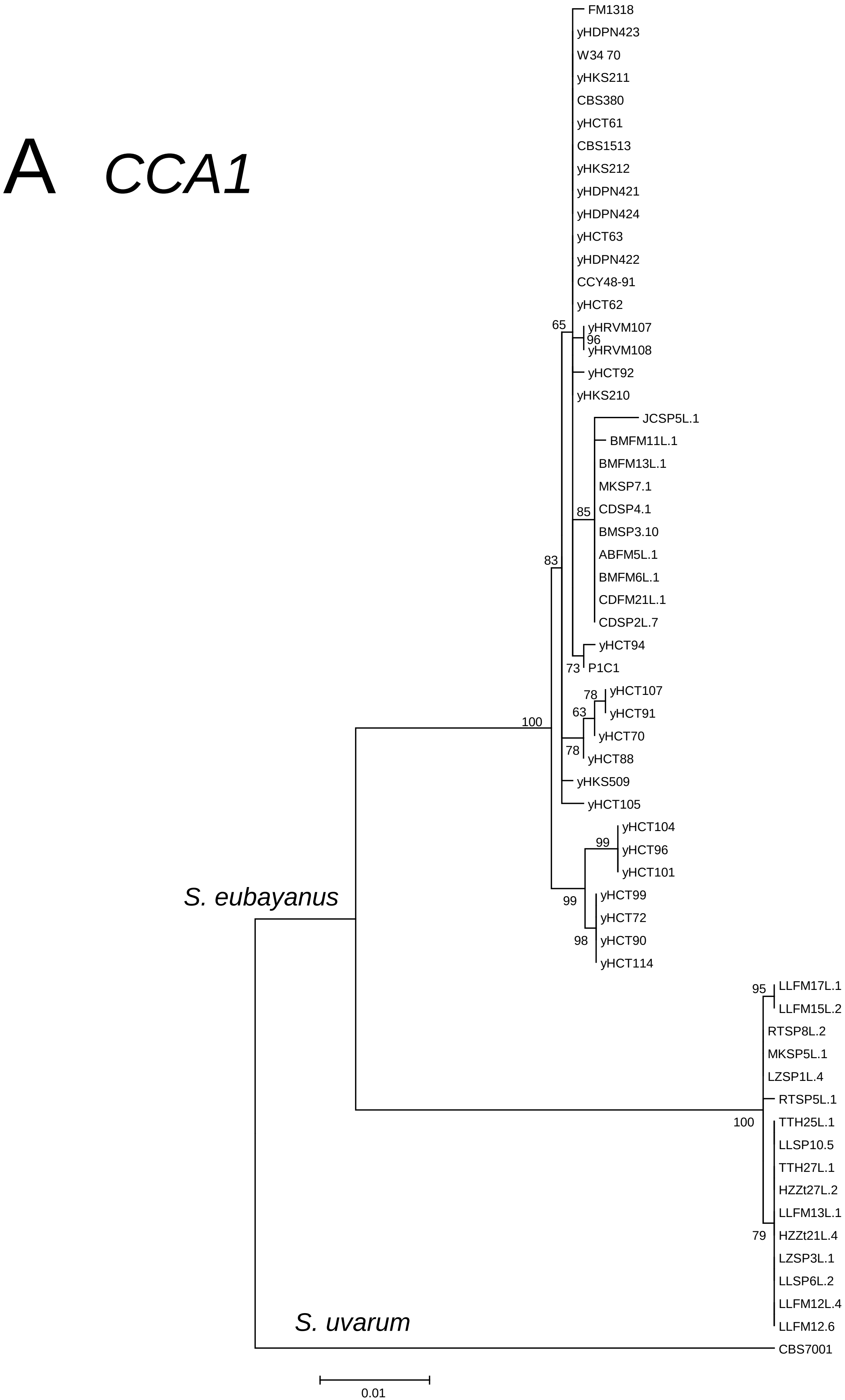
A
CCA1
FM1318
yHDPN423
W34 70
yHKS211
CBS380
yHCT61
CBS1513
yHKS212
yHDPN421
yHDPN424
yHCT63
yHDPN422
CCY48-91
yHCT62
65
yHRVM107
96
yHRVM108
yHCT92
yHKS210
JCSP5L.1
BMFM11L.1
BMFM13L.1
MKSP7.1
CDSP4.1
85
BMSP3.10
ABFM5L.1
83
BMFM6L.1
CDFM21L.1
CDSP2L.7
yHCT94
73
P1C1
78
yHCT107
63
yHCT91
100
yHCT70
78
yHCT88
yHKS509
yHCT105
yHCT104
99
yHCT96
yHCT101
S. eubayanus
99
yHCT99
yHCT72
98
yHCT90
yHCT114
95
LLFM17L.1
LLFM15L.2
RTSP8L.2
MKSP5L.1
LZSP1L.4
RTSP5L.1
100
TTH25L.1
LLSP10.5
TTH27L.1
HZZt27L.2
LLFM13L.1
79
HZZt21L.4
LZSP3L.1
LLSP6L.2
LLFM12L.4
S. uvarum
LLFM12.6
CBS7001
0.01

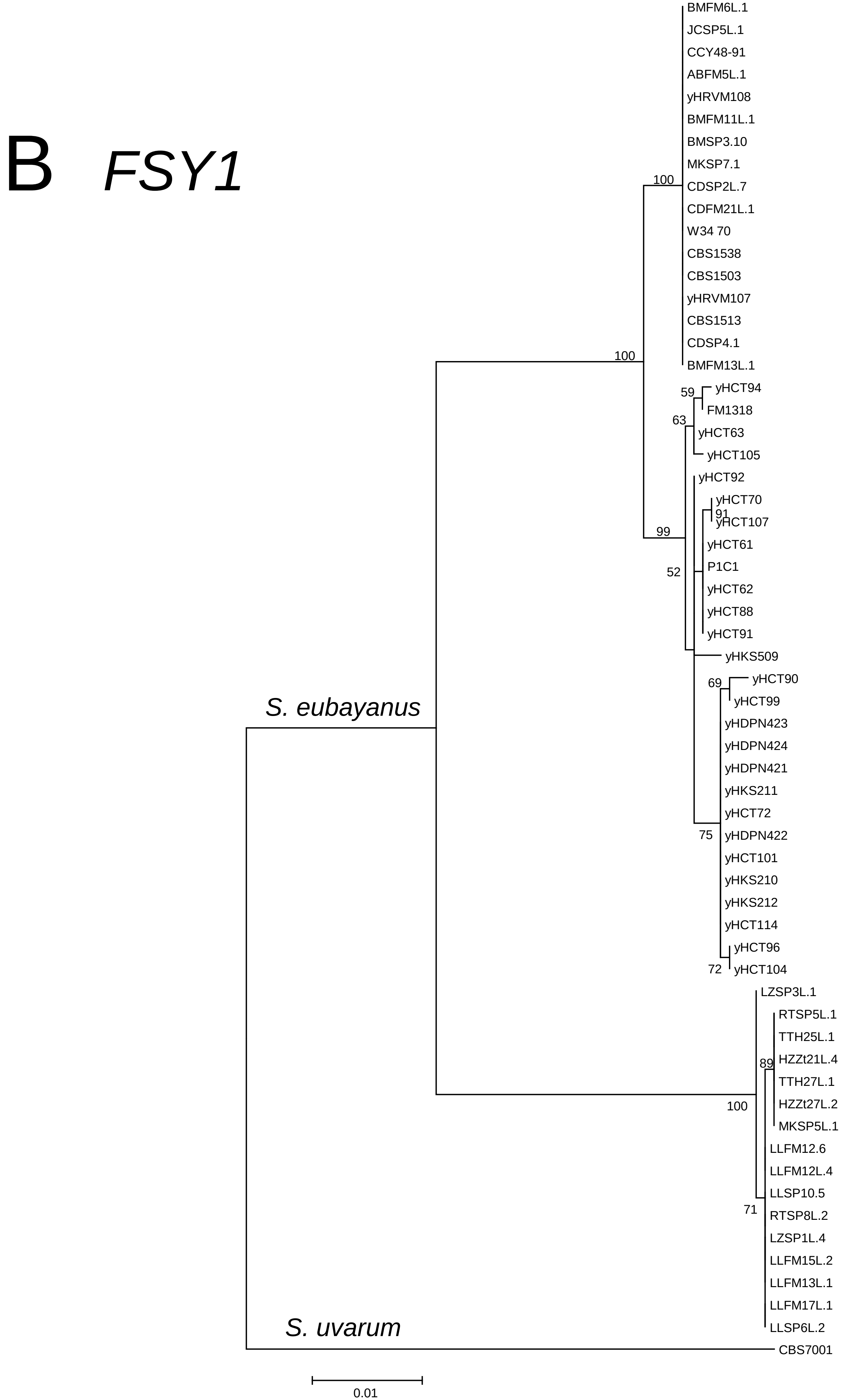
B
FSY1
BMFM6L.1
JCSP5L.1
CCY48-91
ABFM5L.1
yHRVM108
BMFM11L.1
BMSP3.10
MKSP7.1
100
CDSP2L.7
CDFM21L.1
W34 70
CBS1538
CBS1503
yHRVM107
CBS1513
CDSP4.1
100
BMFM13L.1
59
yHCT94
FM1318
63
yHCT63
yHCT105
yHCT92
yHCT70
91
yHCT107
99
yHCT61
52
P1C1
yHCT62
yHCT88
yHCT91
yHKS509
69
yHCT90
yHCT99
S. eubayanus
yHDPN423
yHDPN424
yHDPN421
yHKS211
yHCT72
75
yHDPN422
yHCT101
yHKS210
yHKS212
yHCT114
yHCT96
72
yHCT104
LZSP3L.1
RTSP5L.1
TTH25L.1
89
HZZt21L.4
TTH27L.1
100
HZZt27L.2
MKSP5L.1
LLFM12.6
LLFM12L.4
LLSP10.5
71
RTSP8L.2
LZSP1L.4
LLFM15L.2
LLFM13L.1
LLFM17L.1
S. uvarum
LLSP6L.2
CBS7001
0.01

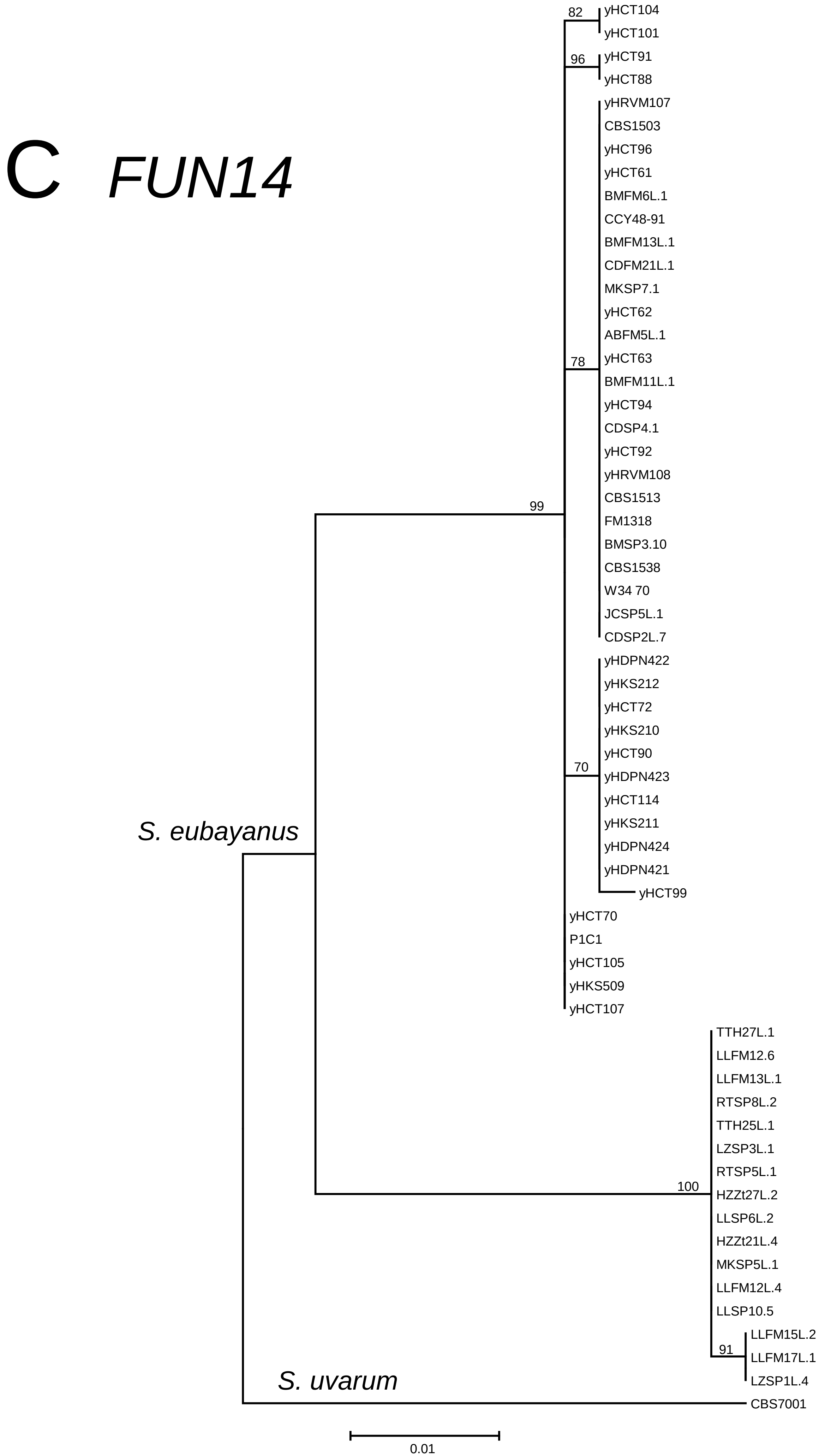
C
FUN14
S. eubayanus
S. uvarum
82
96
78
99
70
100
91
yHCT104
yHCT101
yHCT91
yHCT88
yHRVM107
CBS1503
yHCT96
yHCT61
BMFM6L.1
CCY48-91
BMFM13L.1
CDFM21L.1
MKSP7.1
yHCT62
ABFM5L.1
yHCT63
BMFM11L.1
yHCT94
CDSP4.1
yHCT92
yHRVM108
CBS1513
FM1318
BMSP3.10
CBS1538
W34 70
JCSP5L.1
CDSP2L.7
yHDPN422
yHKS212
yHCT72
yHKS210
yHCT90
yHDPN423
yHCT114
yHKS211
yHDPN424
yHDPN421
yHCT99
yHCT70
P1C1
yHCT105
yHKS509
yHCT107
TTH27L.1
LLFM12.6
LLFM13L.1
RTSP8L.2
TTH25L.1
LZSP3L.1
RTSP5L.1
HZZt27L.2
LLSP6L.2
HZZt21L.4
MKSP5L.1
LLFM12L.4
LLSP10.5
LLFM15L.2
LLFM17L.1
LZSP1L.4
CBS7001
0.01

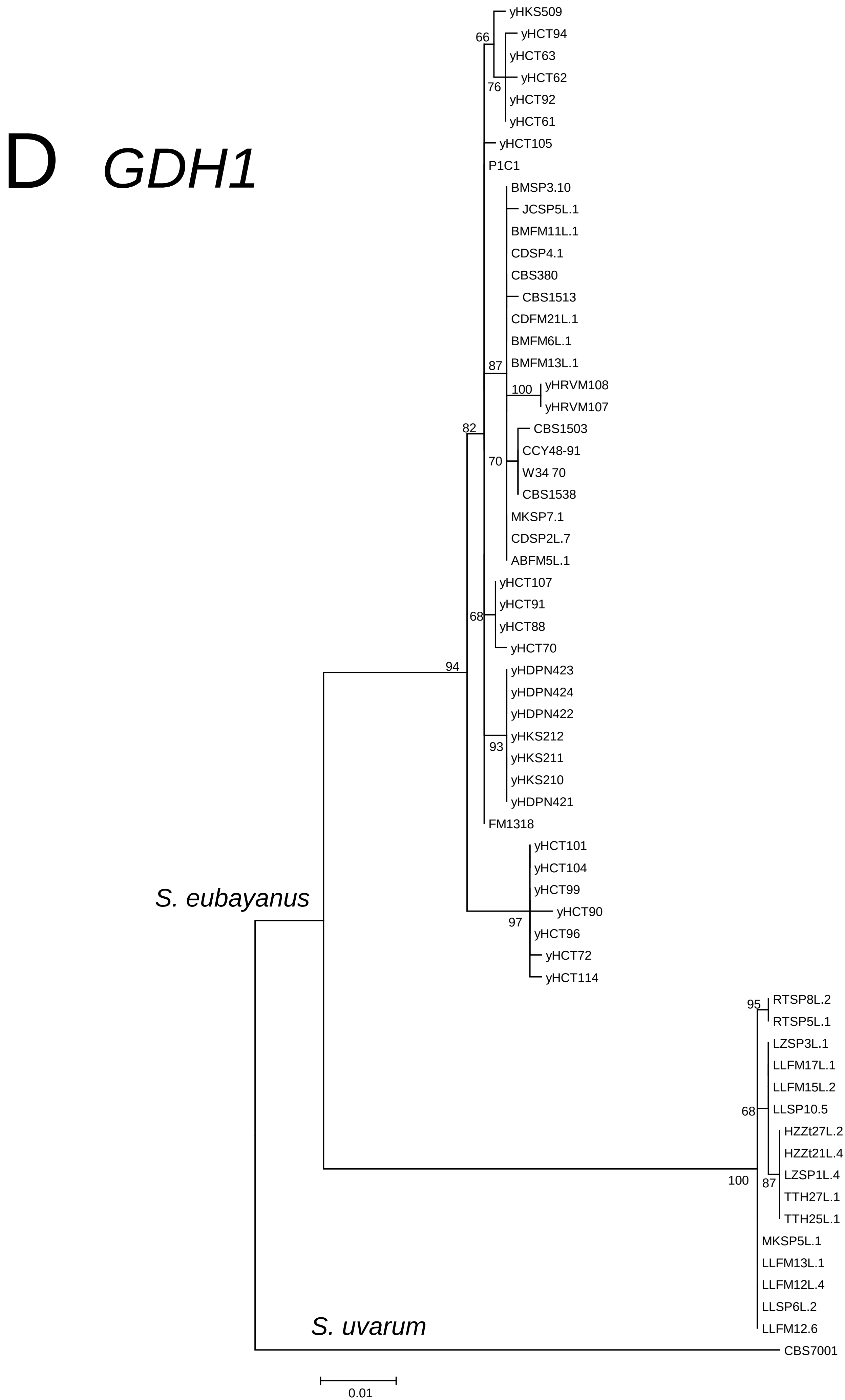
D
GDH1
yHKS509
yHCT94
yHCT63
yHCT62
yHCT92
yHCT61
yHCT105
P1C1
BMSP3.10
JCSP5L.1
BMFM11L.1
CDSP4.1
CBS380
CBS1513
CDFM21L.1
BMFM6L.1
BMFM13L.1
yHRVM108
yHRVM107
CBS1503
CCY48-91
W34 70
CBS1538
MKSP7.1
CDSP2L.7
ABFM5L.1
yHCT107
yHCT91
yHCT88
yHCT70
yHDPN423
yHDPN424
yHDPN422
yHKS212
yHKS211
yHKS210
yHDPN421
FM1318
yHCT101
yHCT104
yHCT99
yHCT90
yHCT96
yHCT72
yHCT114
RTSP8L.2
RTSP5L.1
LZSP3L.1
LLFM17L.1
LLFM15L.2
LLSP10.5
HZZt27L.2
HZZt21L.4
LZSP1L.4
TTH27L.1
TTH25L.1
MKSP5L.1
LLFM13L.1
LLFM12L.4
LLSP6L.2
LLFM12.6
CBS7001
66
76
87
100
82
70
68
94
93
97
95
68
100
87
S. eubayanus
S. uvarum
0.01

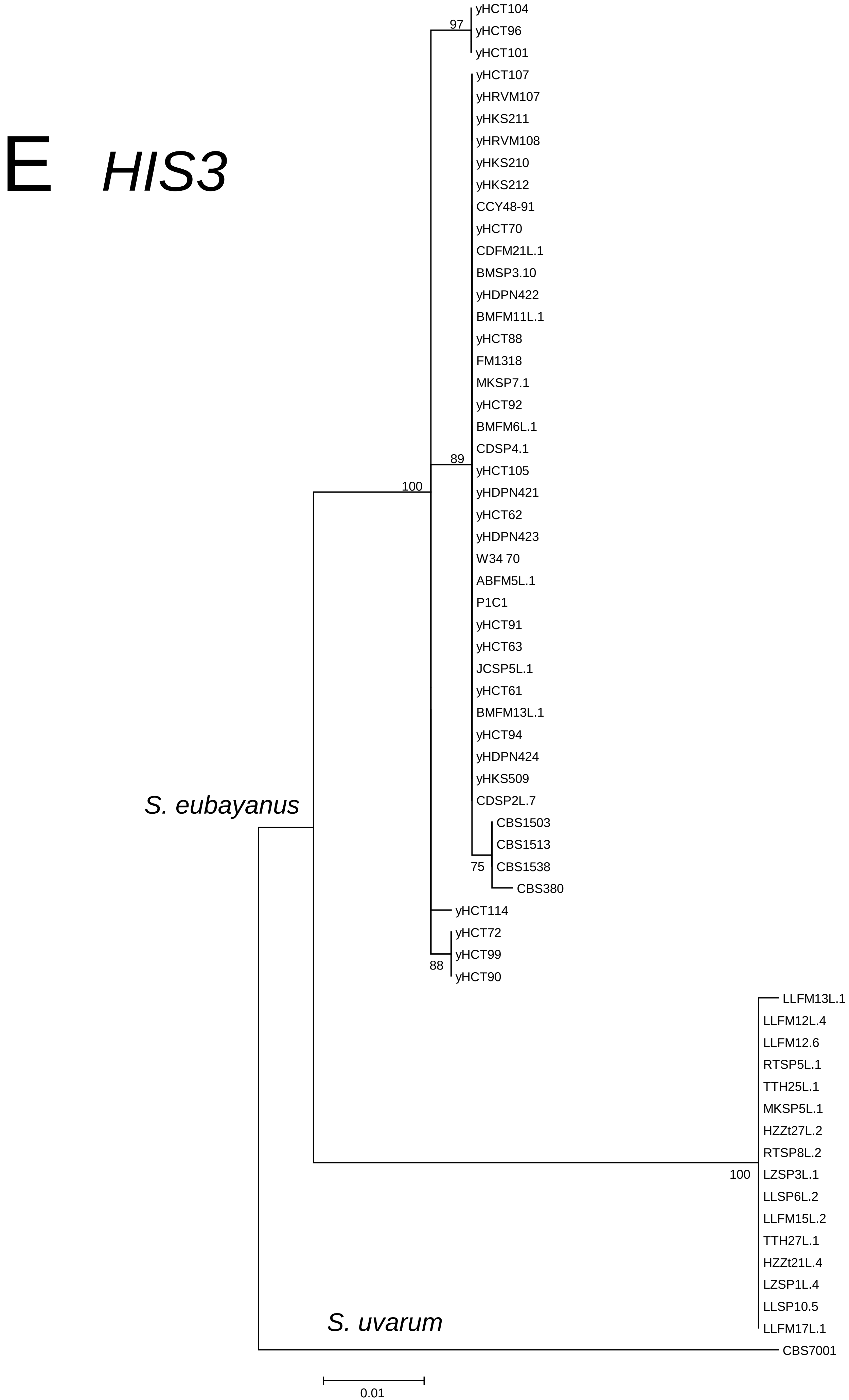
E
HIS3
yHCT104
yHCT96
yHCT101
97
yHCT107
yHRVM107
yHKS211
yHRVM108
yHKS210
yHKS212
CCY48-91
yHCT70
CDFM21L.1
BMSP3.10
yHDPN422
BMFM11L.1
yHCT88
FM1318
MKSP7.1
yHCT92
BMFM6L.1
CDSP4.1
89
yHCT105
100
yHDPN421
yHCT62
yHDPN423
W34 70
ABFM5L.1
P1C1
yHCT91
yHCT63
JCSP5L.1
yHCT61
BMFM13L.1
yHCT94
yHDPN424
yHKS509
S. eubayanus
CDSP2L.7
CBS1503
CBS1513
75
CBS1538
CBS380
yHCT114
yHCT72
yHCT99
88
yHCT90
LLFM13L.1
LLFM12L.4
LLFM12.6
RTSP5L.1
TTH25L.1
MKSP5L.1
HZZt27L.2
RTSP8L.2
100
LZSP3L.1
LLSP6L.2
LLFM15L.2
TTH27L.1
HZZt21L.4
LZSP1L.4
LLSP10.5
S. uvarum
LLFM17L.1
CBS7001
0.01

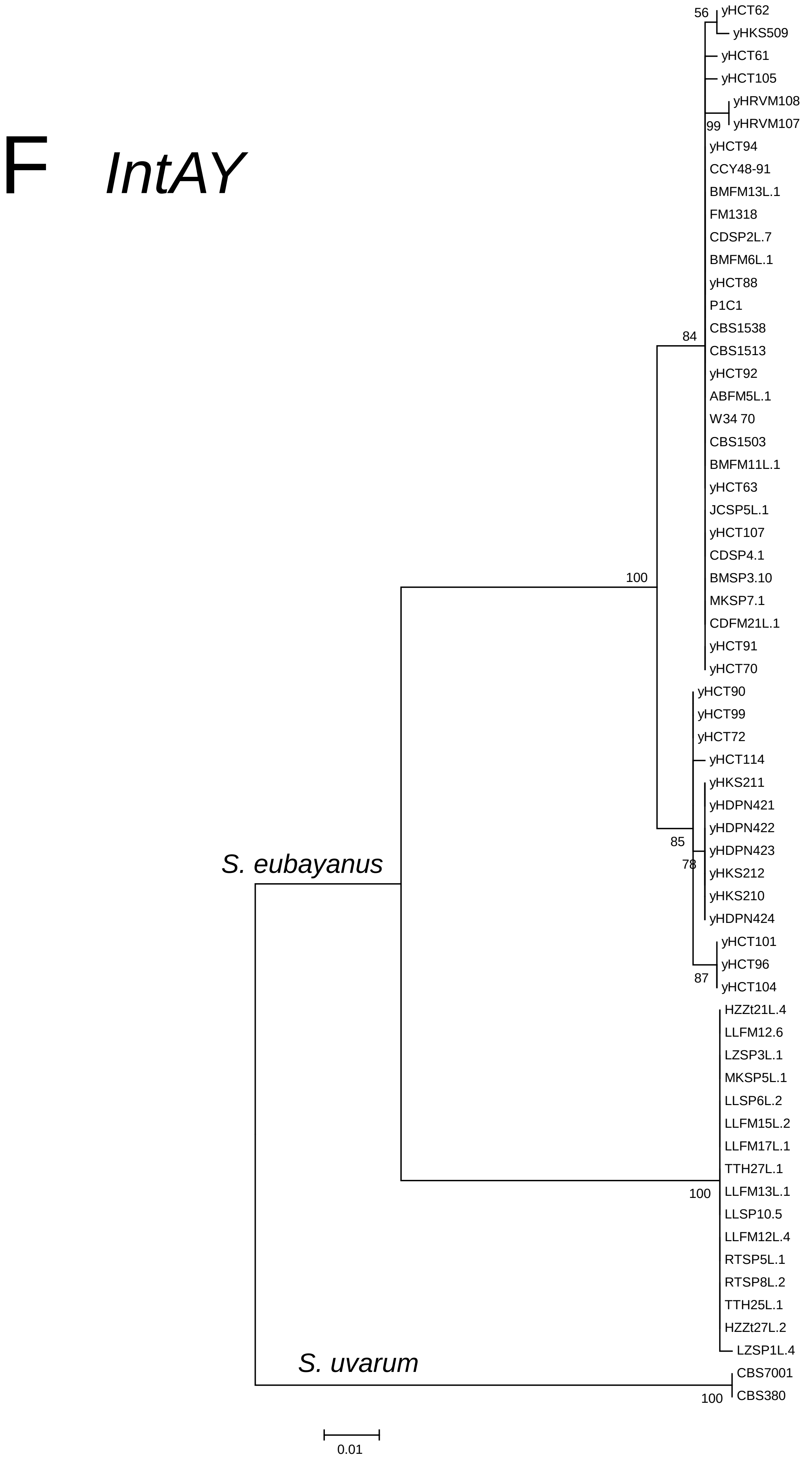
F
IntAY
56
yHCT62
yHKS509
yHCT61
yHCT105
yHRVM108
99
yHRVM107
yHCT94
CCY48-91
BMFM13L.1
FM1318
CDSP2L.7
BMFM6L.1
yHCT88
P1C1
84
CBS1538
CBS1513
yHCT92
ABFM5L.1
W34 70
CBS1503
BMFM11L.1
yHCT63
JCSP5L.1
yHCT107
CDSP4.1
100
BMSP3.10
MKSP7.1
CDFM21L.1
yHCT91
yHCT70
yHCT90
yHCT99
yHCT72
yHCT114
yHKS211
yHDPN421
yHDPN422
85
yHDPN423
78
S. eubayanus
yHKS212
yHKS210
yHDPN424
yHCT101
yHCT96
87
yHCT104
HZZt21L.4
LLFM12.6
LZSP3L.1
MKSP5L.1
LLSP6L.2
LLFM15L.2
LLFM17L.1
TTH27L.1
100
LLFM13L.1
LLSP10.5
LLFM12L.4
RTSP5L.1
RTSP8L.2
TTH25L.1
HZZt27L.2
LZSP1L.4
S. uvarum
CBS7001
100
CBS380
0.01

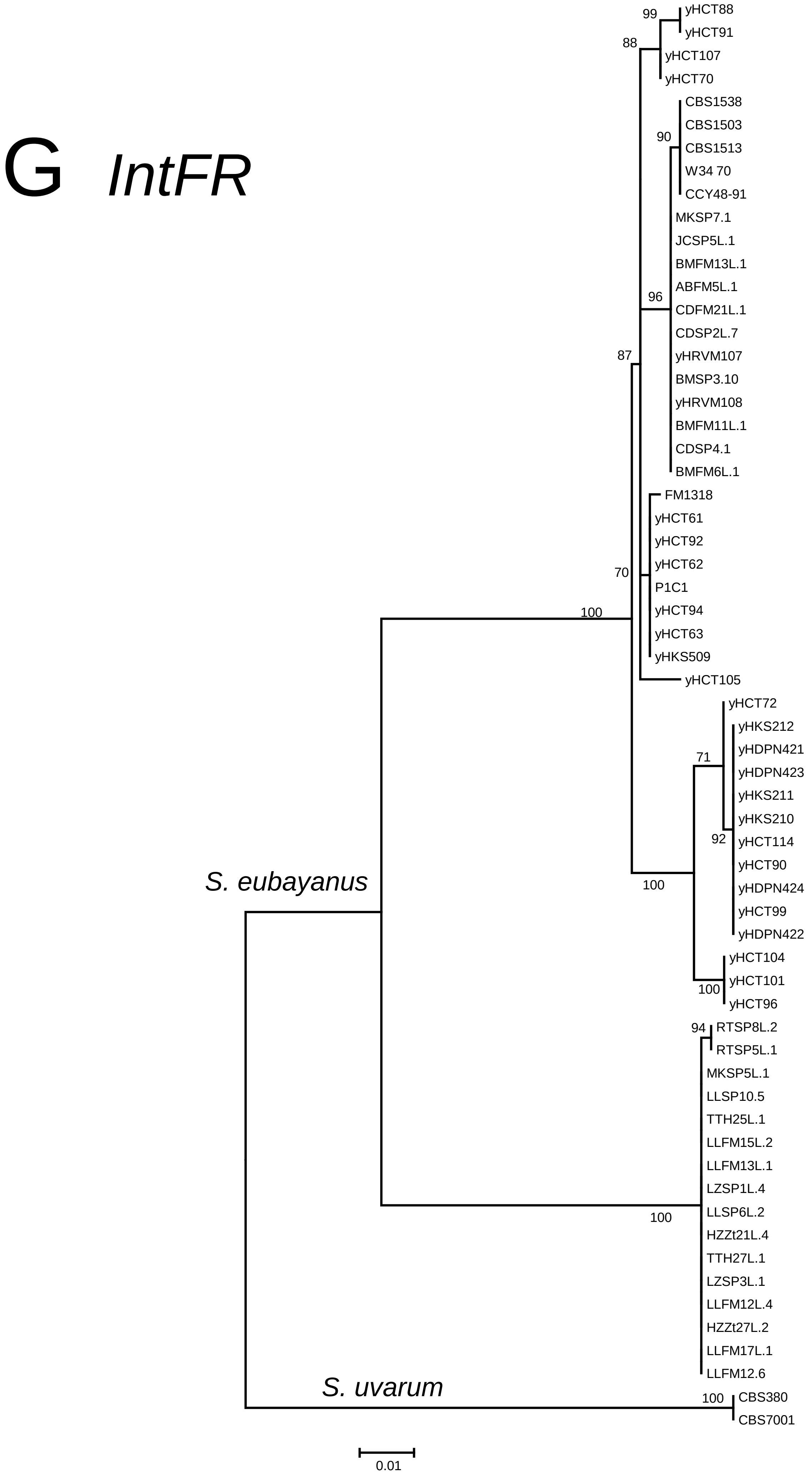
G
IntFR
yHCT88
yHCT91
yHCT107
yHCT70
CBS1538
CBS1503
CBS1513
W34 70
CCY48-91
MKSP7.1
JCSP5L.1
BMFM13L.1
ABFM5L.1
CDFM21L.1
CDSP2L.7
yHRVM107
BMSP3.10
yHRVM108
BMFM11L.1
CDSP4.1
BMFM6L.1
FM1318
yHCT61
yHCT92
yHCT62
P1C1
yHCT94
yHCT63
yHKS509
yHCT105
yHCT72
yHKS212
yHDPN421
yHDPN423
yHKS211
yHKS210
yHCT114
yHCT90
yHDPN424
yHCT99
yHDPN422
yHCT104
yHCT101
yHCT96
RTSP8L.2
RTSP5L.1
MKSP5L.1
LLSP10.5
TTH25L.1
LLFM15L.2
LLFM13L.1
LZSP1L.4
LLSP6L.2
HZZt21L.4
TTH27L.1
LZSP3L.1
LLFM12L.4
HZZt27L.2
LLFM17L.1
LLFM12.6
CBS380
CBS7001
S. eubayanus
S. uvarum
99
88
90
96
87
70
100
71
92
100
100
94
100
100
0.01

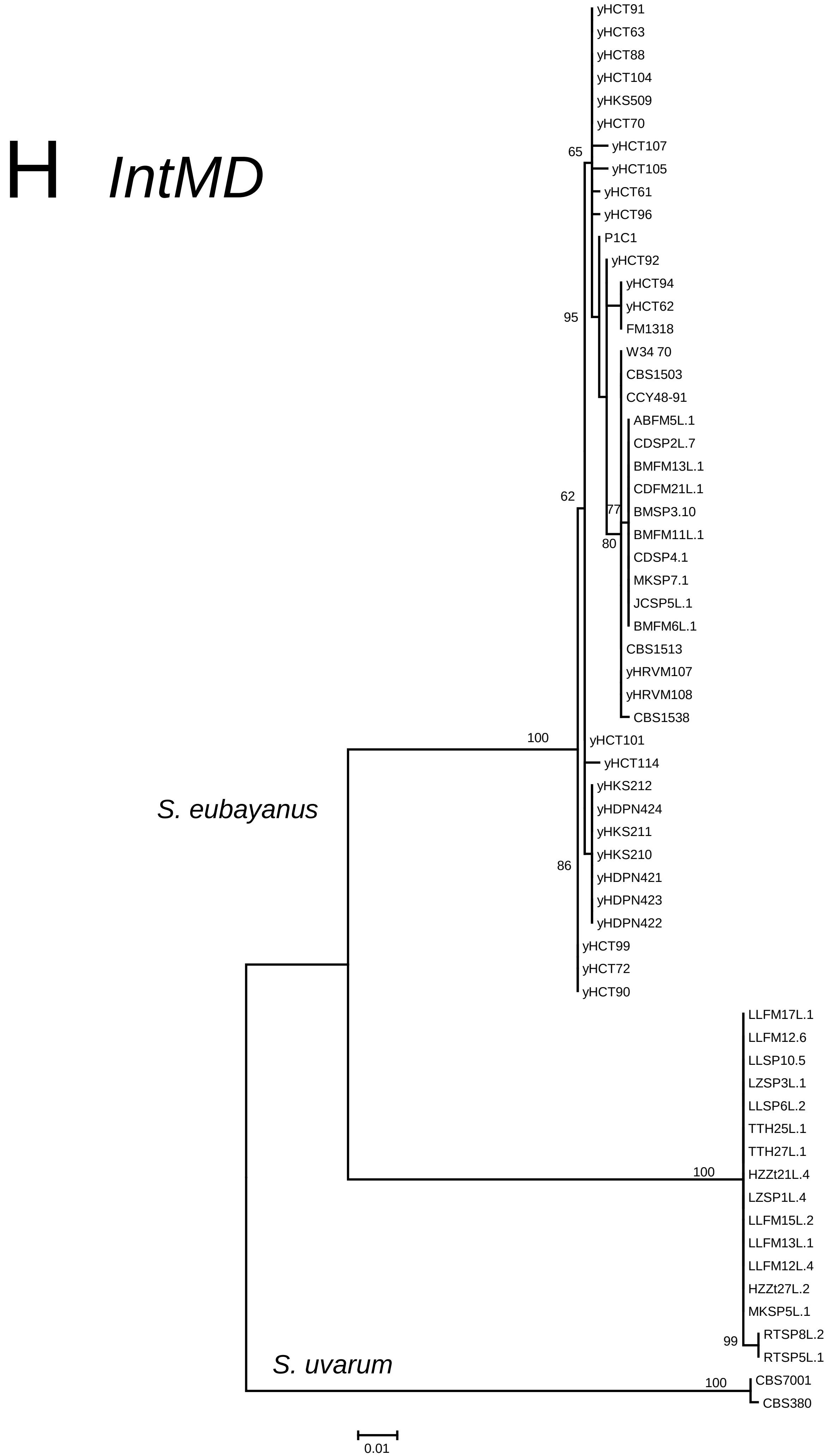
H
IntMD
yHCT91
yHCT63
yHCT88
yHCT104
yHKS509
yHCT70
yHCT107
yHCT105
yHCT61
yHCT96
P1C1
yHCT92
yHCT94
yHCT62
FM1318
W34 70
CBS1503
CCY48-91
ABFM5L.1
CDSP2L.7
BMFM13L.1
CDFM21L.1
BMSP3.10
BMFM11L.1
CDSP4.1
MKSP7.1
JCSP5L.1
BMFM6L.1
CBS1513
yHRVM107
yHRVM108
CBS1538
yHCT101
yHCT114
yHKS212
yHDPN424
yHKS211
yHKS210
yHDPN421
yHDPN423
yHDPN422
yHCT99
yHCT72
yHCT90
LLFM17L.1
LLFM12.6
LLSP10.5
LZSP3L.1
LLSP6L.2
TTH25L.1
TTH27L.1
HZZt21L.4
LZSP1L.4
LLFM15L.2
LLFM13L.1
LLFM12L.4
HZZt27L.2
MKSP5L.1
RTSP8L.2
RTSP5L.1
CBS7001
CBS380
65
95
62
77
80
100
86
100
99
100
S. eubayanus
S. uvarum
0.01

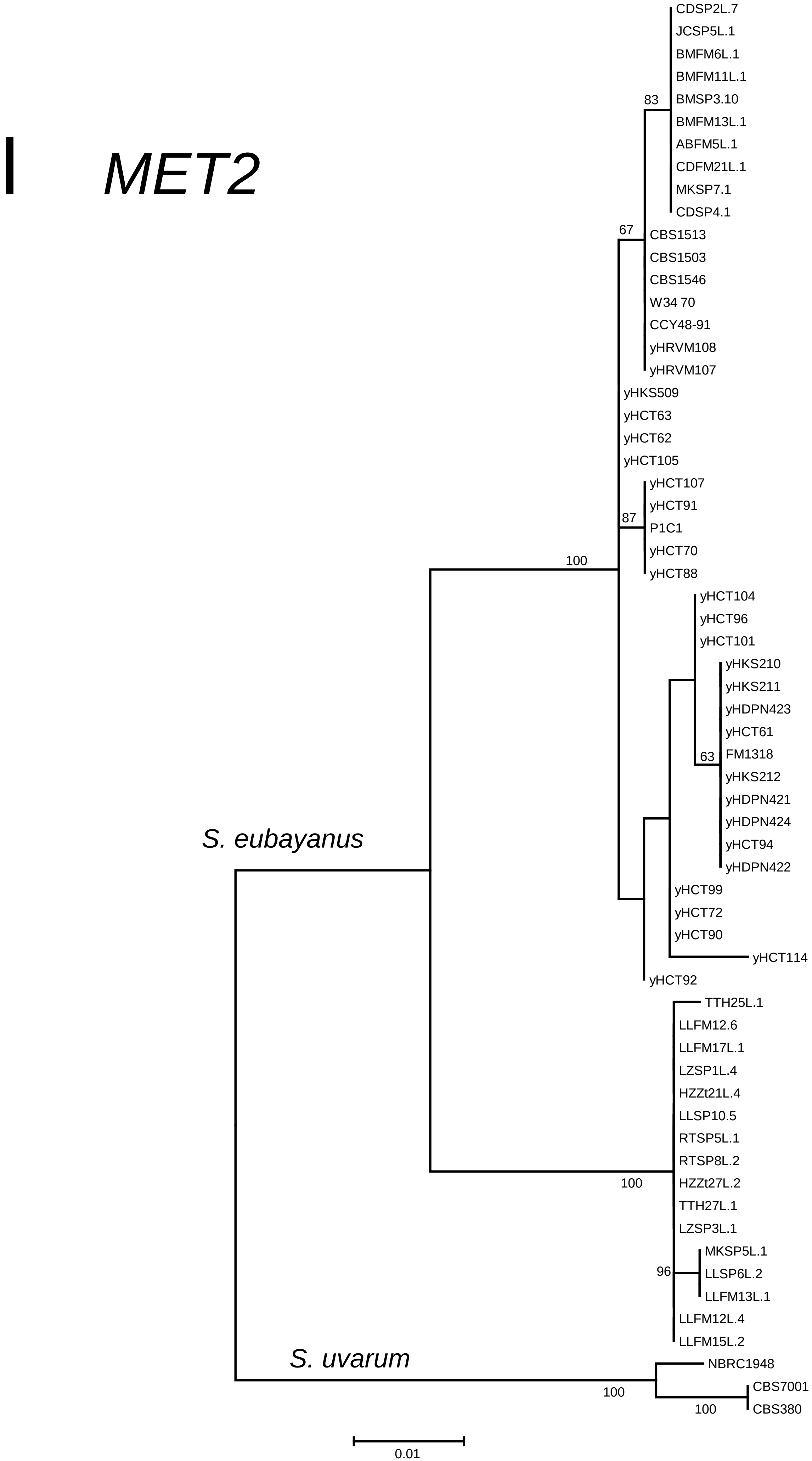
I
MET2
S. eubayanus
S. uvarum
CDSP2L.7
JCSP5L.1
BMFM6L.1
BMFM11L.1
BMSP3.10
BMFM13L.1
ABFM5L.1
CDFM21L.1
MKSP7.1
CDSP4.1
CBS1513
CBS1503
CBS1546
W34 70
CCY48-91
yHRVM108
yHRVM107
yHKS509
yHCT63
yHCT62
yHCT105
yHCT107
yHCT91
P1C1
yHCT70
yHCT88
yHCT104
yHCT96
yHCT101
yHKS210
yHKS211
yHDPN423
yHCT61
FM1318
yHKS212
yHDPN421
yHDPN424
yHCT94
yHDPN422
yHCT99
yHCT72
yHCT90
yHCT114
yHCT92
TTH25L.1
LLFM12.6
LLFM17L.1
LZSP1L.4
HZZt21L.4
LLSP10.5
RTSP5L.1
RTSP8L.2
HZZt27L.2
TTH27L.1
LZSP3L.1
MKSP5L.1
LLSP6L.2
LLFM13L.1
LLFM12L.4
LLFM15L.2
NBRC1948
CBS7001
CBS380
83
67
87
100
63
100
96
100
100
0.01

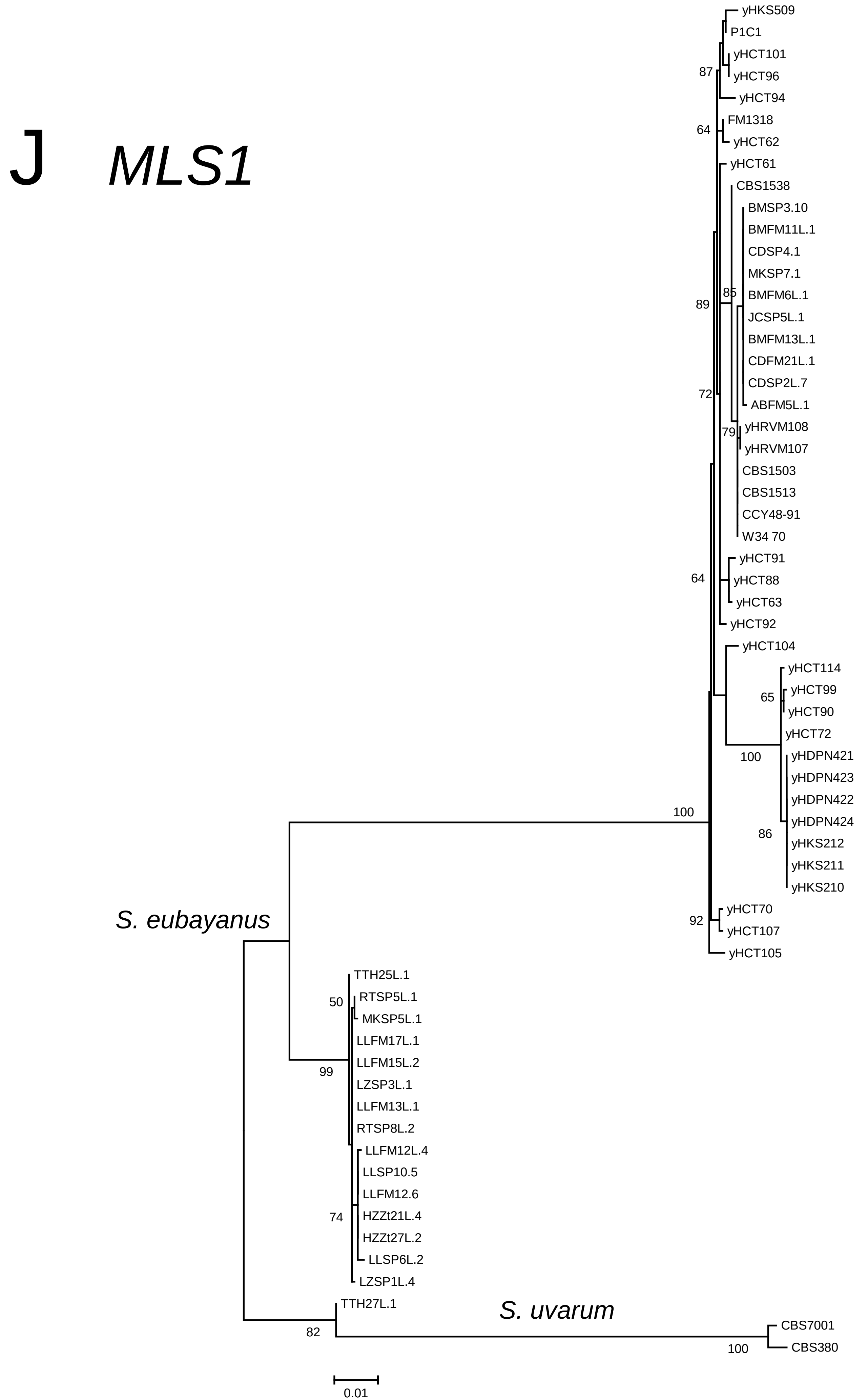
J
MLS1
yHKS509
P1C1
yHCT101
yHCT96
yHCT94
FM1318
yHCT62
yHCT61
CBS1538
BMSP3.10
BMFM11L.1
CDSP4.1
MKSP7.1
BMFM6L.1
JCSP5L.1
BMFM13L.1
CDFM21L.1
CDSP2L.7
ABFM5L.1
yHRVM108
yHRVM107
CBS1503
CBS1513
CCY48-91
W34 70
yHCT91
yHCT88
yHCT63
yHCT92
yHCT104
yHCT114
yHCT99
yHCT90
yHCT72
yHDPN421
yHDPN423
yHDPN422
yHDPN424
yHKS212
yHKS211
yHKS210
yHCT70
yHCT107
yHCT105
87
64
89
85
72
79
64
65
100
86
100
92
S. eubayanus
TTH25L.1
RTSP5L.1
MKSP5L.1
LLFM17L.1
LLFM15L.2
LZSP3L.1
LLFM13L.1
RTSP8L.2
LLFM12L.4
LLSP10.5
LLFM12.6
HZZt21L.4
HZZt27L.2
LLSP6L.2
LZSP1L.4
50
99
74
TTH27L.1
S. uvarum
CBS7001
CBS380
82
100
0.01

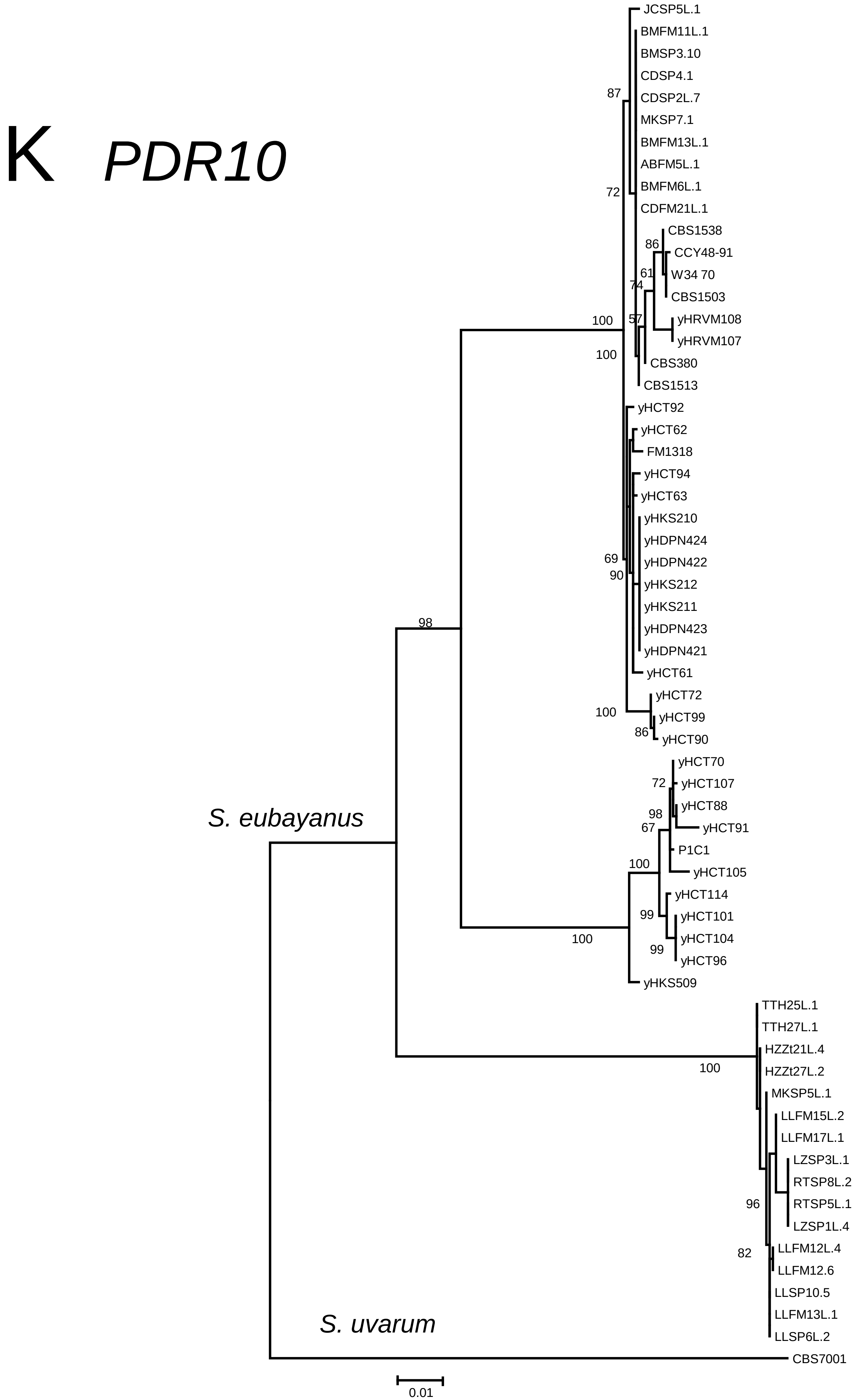

K PDR10
JCSP5L.1
BMFM11L.1
BMSP3.10
CDSP4.1
CDSP2L.7
MKSP7.1
BMFM13L.1
ABFM5L.1
BMFM6L.1
CDFM21L.1
CBS1538
CCY48-91
W34 70
CBS1503
yHRVM108
yHRVM107
CBS380
CBS1513
yHCT92
yHCT62
FM1318
yHCT94
yHCT63
yHKS210
yHDPN424
yHDPN422
yHKS212
yHKS211
yHDPN423
yHDPN421
yHCT61
yHCT72
yHCT99
yHCT90
yHCT70
yHCT107
yHCT88
yHCT91
P1C1
yHCT105
yHCT114
yHCT101
yHCT104
yHCT96
yHKS509
TTH25L.1
TTH27L.1
HZZt21L.4
HZZt27L.2
MKSP5L.1
LLFM15L.2
LLFM17L.1
LZSP3L.1
RTSP8L.2
RTSP5L.1
LZSP1L.4
LLFM12L.4
LLFM12.6
LLSP10.5
LLFM13L.1
LLSP6L.2
CBS7001
S. eubayanus
S. uvarum
0.01

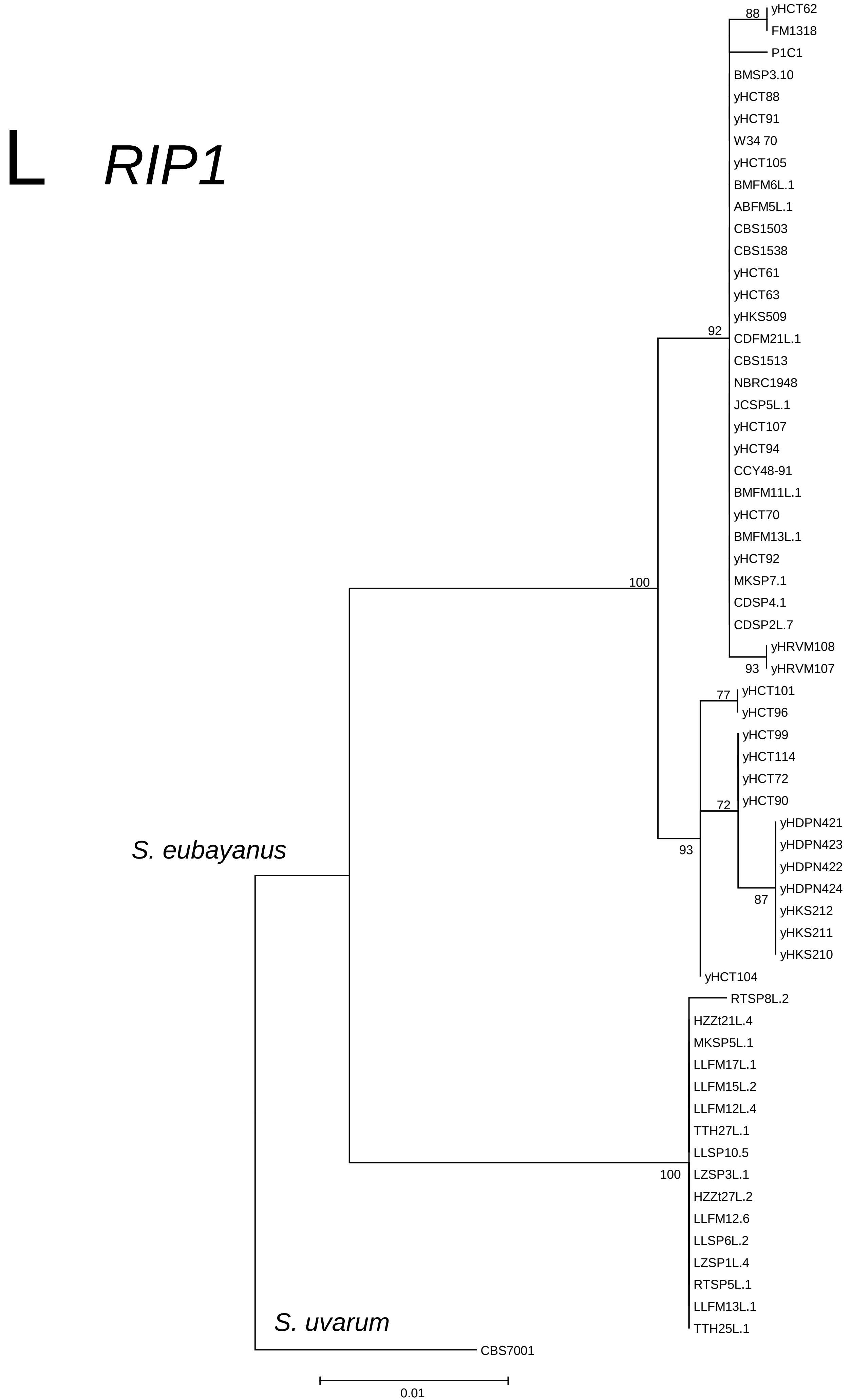
L
RIP1
S. eubayanus
S. uvarum
yHCT62
FM1318
P1C1
BMSP3.10
yHCT88
yHCT91
W34 70
yHCT105
BMFM6L.1
ABFM5L.1
CBS1503
CBS1538
yHCT61
yHCT63
yHKS509
CDFM21L.1
CBS1513
NBRC1948
JCSP5L.1
yHCT107
yHCT94
CCY48-91
BMFM11L.1
yHCT70
BMFM13L.1
yHCT92
MKSP7.1
CDSP4.1
CDSP2L.7
yHRVM108
yHRVM107
yHCT101
yHCT96
yHCT99
yHCT114
yHCT72
yHCT90
yHDPN421
yHDPN423
yHDPN422
yHDPN424
yHKS212
yHKS211
yHKS210
yHCT104
RTSP8L.2
HZZt21L.4
MKSP5L.1
LLFM17L.1
LLFM15L.2
LLFM12L.4
TTH27L.1
LLSP10.5
LZSP3L.1
HZZt27L.2
LLFM12.6
LLSP6L.2
LZSP1L.4
RTSP5L.1
LLFM13L.1
TTH25L.1
CBS7001
88
92
100
93
77
72
93
87
100
0.01

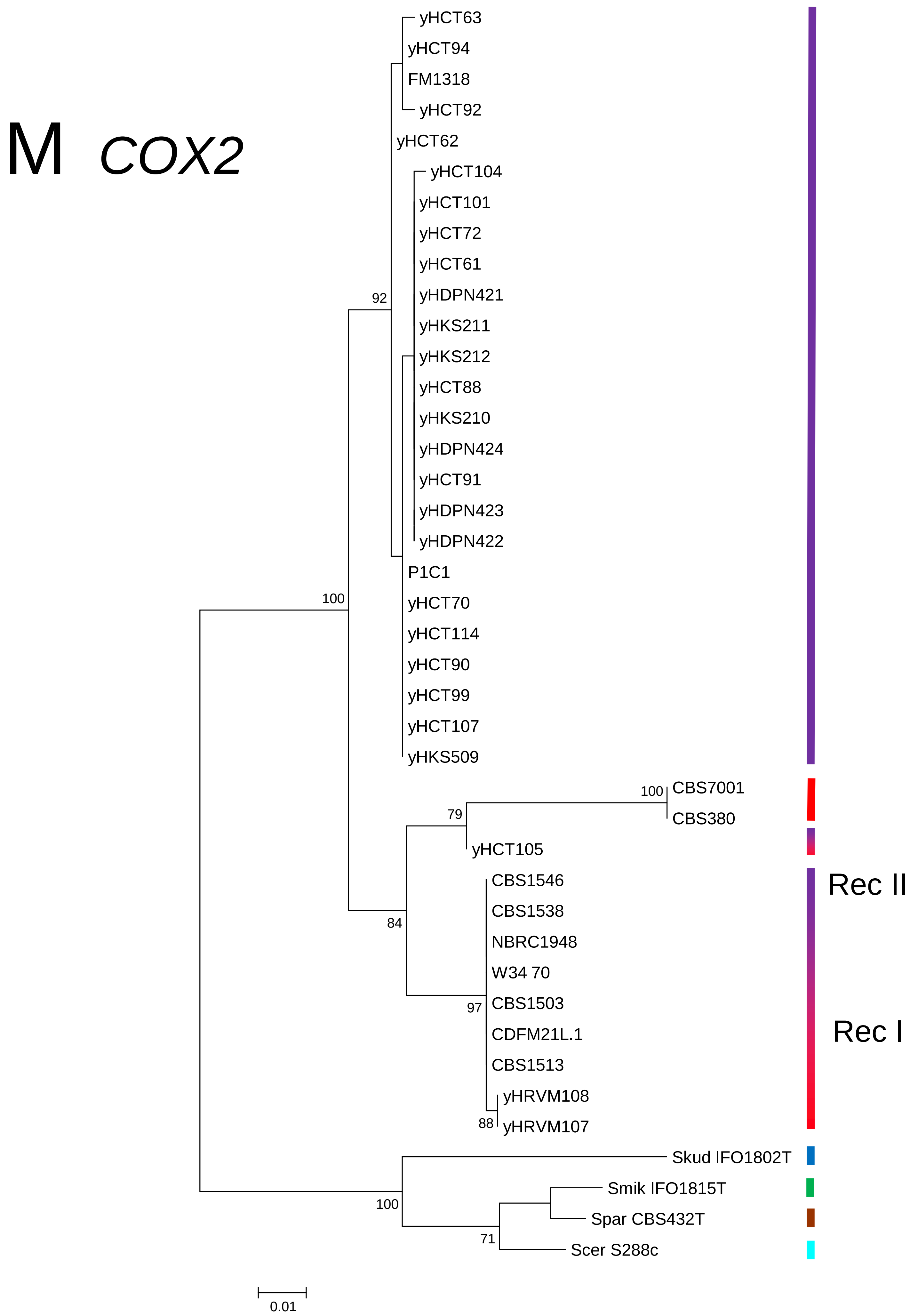
M
COX2
yHCT63
yHCT94
FM1318
yHCT92
yHCT62
yHCT104
yHCT101
yHCT72
yHCT61
yHDPN421
yHKS211
yHKS212
yHCT88
yHKS210
yHDPN424
yHCT91
yHDPN423
yHDPN422
P1C1
yHCT70
yHCT114
yHCT90
yHCT99
yHCT107
yHKS509
92
100
CBS7001
CBS380
100
79
yHCT105
CBS1546
CBS1538
NBRC1948
W34 70
CBS1503
CDFM21L.1
CBS1513
yHRVM108
yHRVM107
84
97
88
Rec II
Rec I
Skud IFO1802T
Smik IFO1815T
Spar CBS432T
Scer S288c
100
71
0.01
