# Supplementary material for: Complex Ancestries of Lager-Brewing Hybrids Were Shaped by Standing Variation in the Wild Yeast Saccharomyces eubayanus
Source: PLoS Genet. 2016 Jul 6;12(7):e1006155. doi: 10.1371/journal.pgen.1006155 (PMC4934787; doi:10.1371/journal.pgen.1006155)
Supplement: S9 Fig — Coverage levels normalized using the median value of coverage for the complete genome are shown for the S. eubayanus subgenome in the Saaz (CBS1503) and Frohberg (W34/70) in A) and B). Normalized coverage levels for non-hybrid strains of S. eubayanus are shown in C) CDFM21L.1, D) yHRVM108, E) yHCT61, F) yHCT70, G) yHCT96, H) yHCT114, I) yHKS212, and J) FM1318. The chromosome copy numbers of hybrids were inferred by establishing the lowest average coverage values for one copy (i.e. chromosome II of the Saaz, CBS1503, and chromosome I of the Frohberg, W34/70). Absolute counts of homozygous and heterozygous SNPs (using 50-kbp windows) compared with the FM1318 reference genome are shown in the bottom graph for each strain. High levels of heterozygosity were detected in subtelomeric regions and a handful of other regions outside of the regions of interest (S9 Fig). These regions of high heterozygosity were shared among strains, including the monosporic and homozygous strain FM1318 (panel J), suggesting they were false positives. The regions of interest (S8 Fig) have less heterozygosity (1.08*10−4 and 8.49*10−5 heterozygous site/bp for Saaz and Frohberg, respectively) than the average heterozygosity detected genome-wide (2.08*10−4 and 4.86*10−4 heterozygous site/bp for Saaz and Frohberg, respectively). Moreover, heterozygosity was not positively correlated with an increase in the number of copies inferred (linear regression r2 = 0.097, p-value = 0.381). Nucleotide diversity levels of the annotated genes within the regions of interest (S8 Fig, S2 Table) were, in general, lower than the average value found genome-wide among the strains from the Patagonia A-Patagonia B-Holarctic clade (0.57%). For 14 of 44 genes the values were higher but less than twice the genome-wide diversity values. Based on comparisons to the multi-locus dataset, the false positive rate of our pipeline at calling non-heterozygous sites was low (4.63*10−5SNPs/site) and not sufficient to influence conclu [file pgen.1006155.s016.pdf]

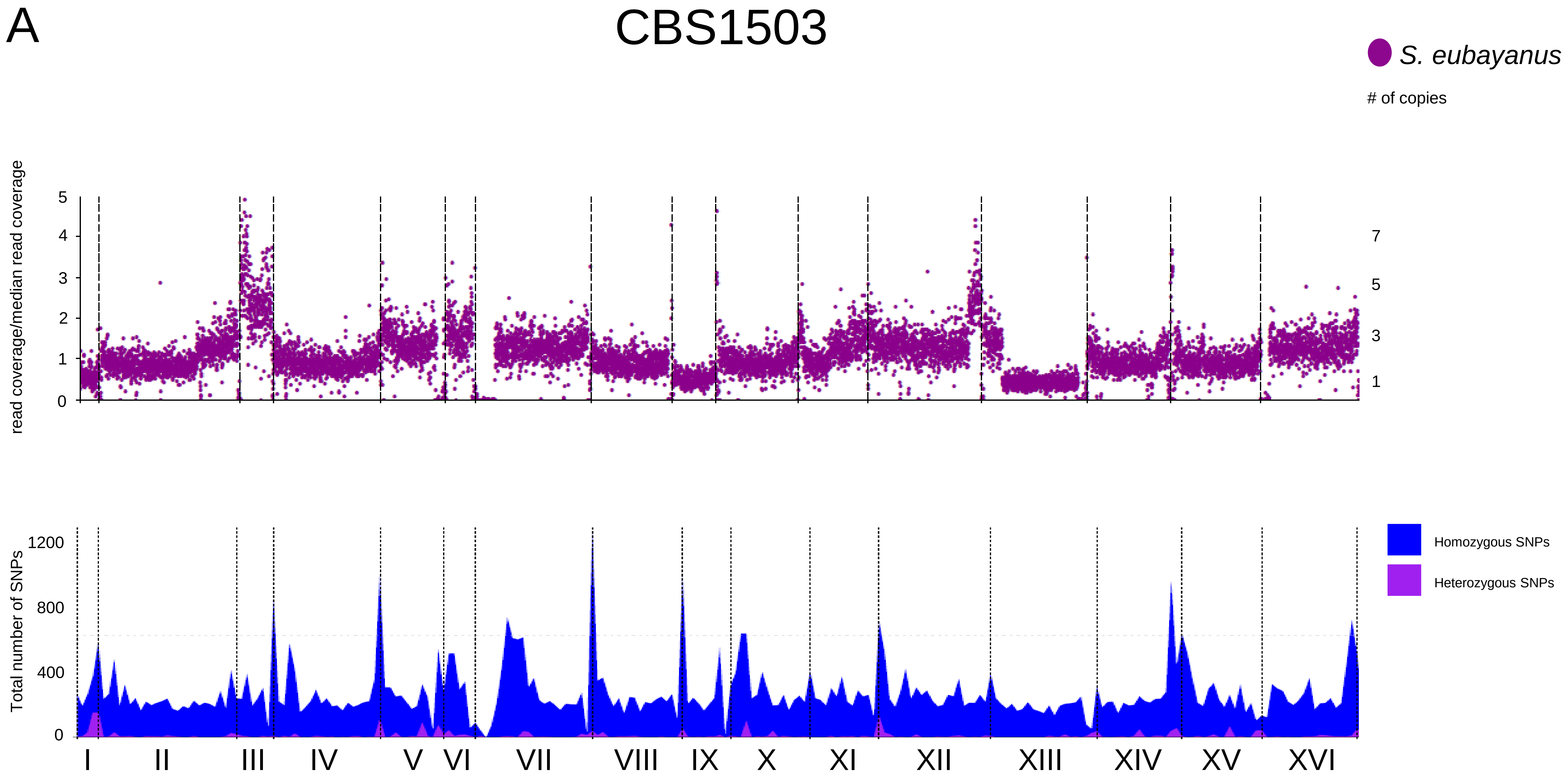
A
CBS1503
S. eubayanus
# of copies
read coverage/median read coverage
5
4
3
2
1
0
7
5
3
1
Total number of SNPs
1200
800
400
0
Homozygous SNPs
Heterozygous SNPs
I
II
III
IV
V
VI
VII
VIII
IX
X
XI
XII
XIII
XIV
XV
XVI

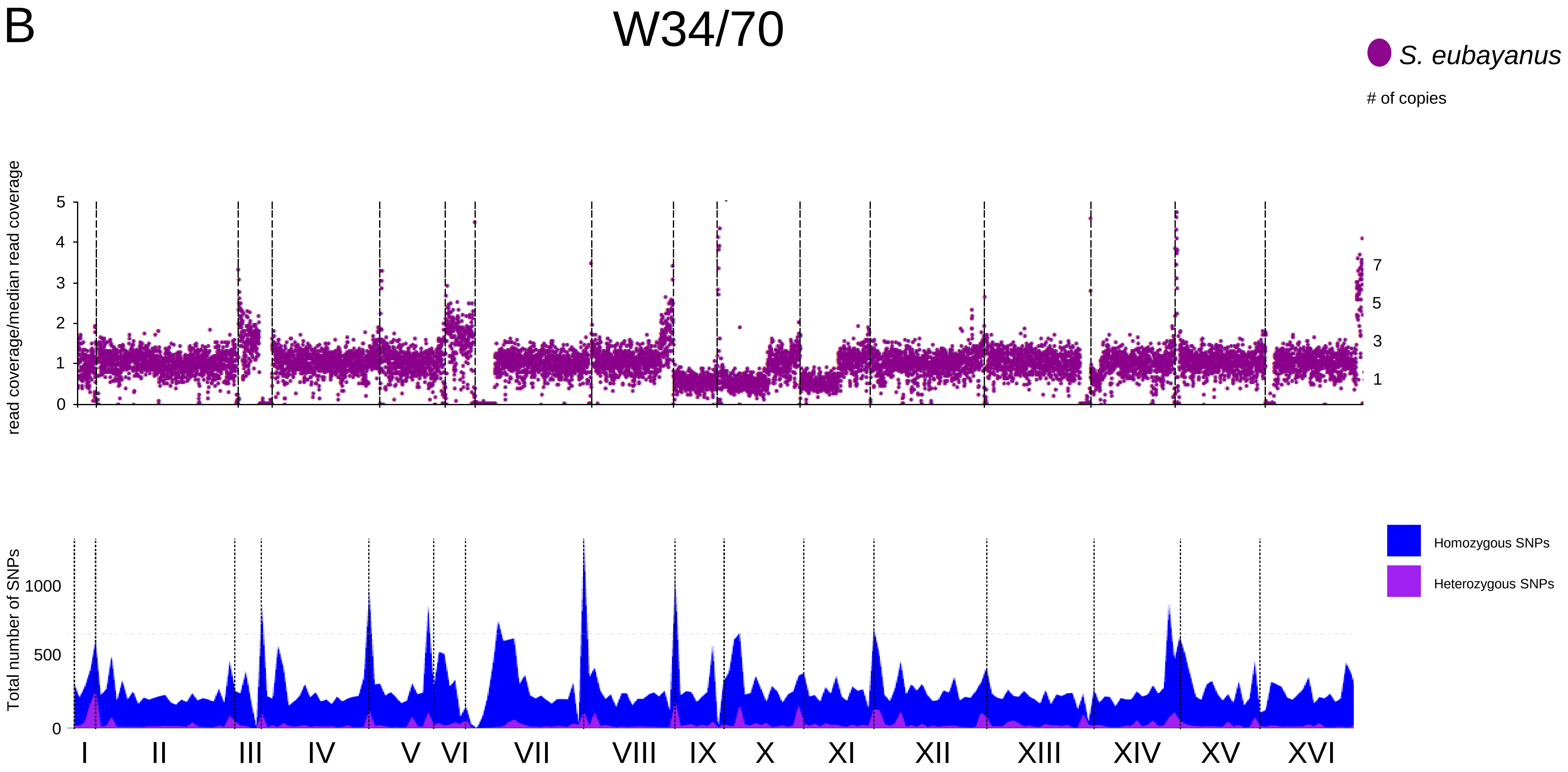

B
W34/70
S. eubayanus
# of copies
read coverage/median read coverage
0
1
2
3
4
5
1
3
5
7
Homozygous SNPs
Heterozygous SNPs
Total number of SNPs
0
500
1000
I
II
III
IV
V
VI
VII
VIII
IX
X
XI
XII
XIII
XIV
XV
XVI

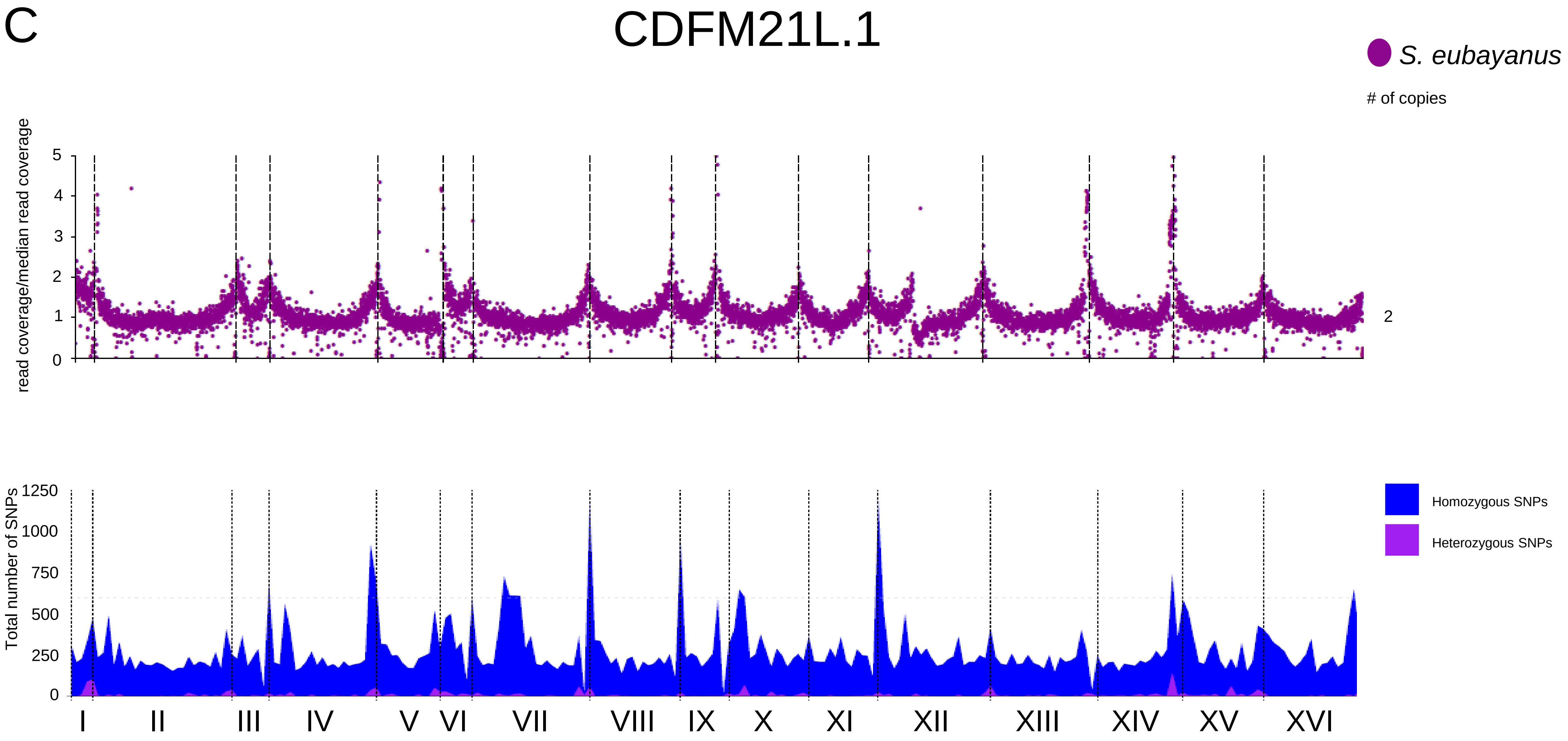
C
CDFM21L.1
S. eubayanus
# of copies
read coverage/median read coverage
5
4
3
2
1
0
2
Total number of SNPs
1250
1000
750
500
250
0
Homozygous SNPs
Heterozygous SNPs
I
II
III
IV
V
VI
VII
VIII
IX
X
XI
XII
XIII
XIV
XV
XVI

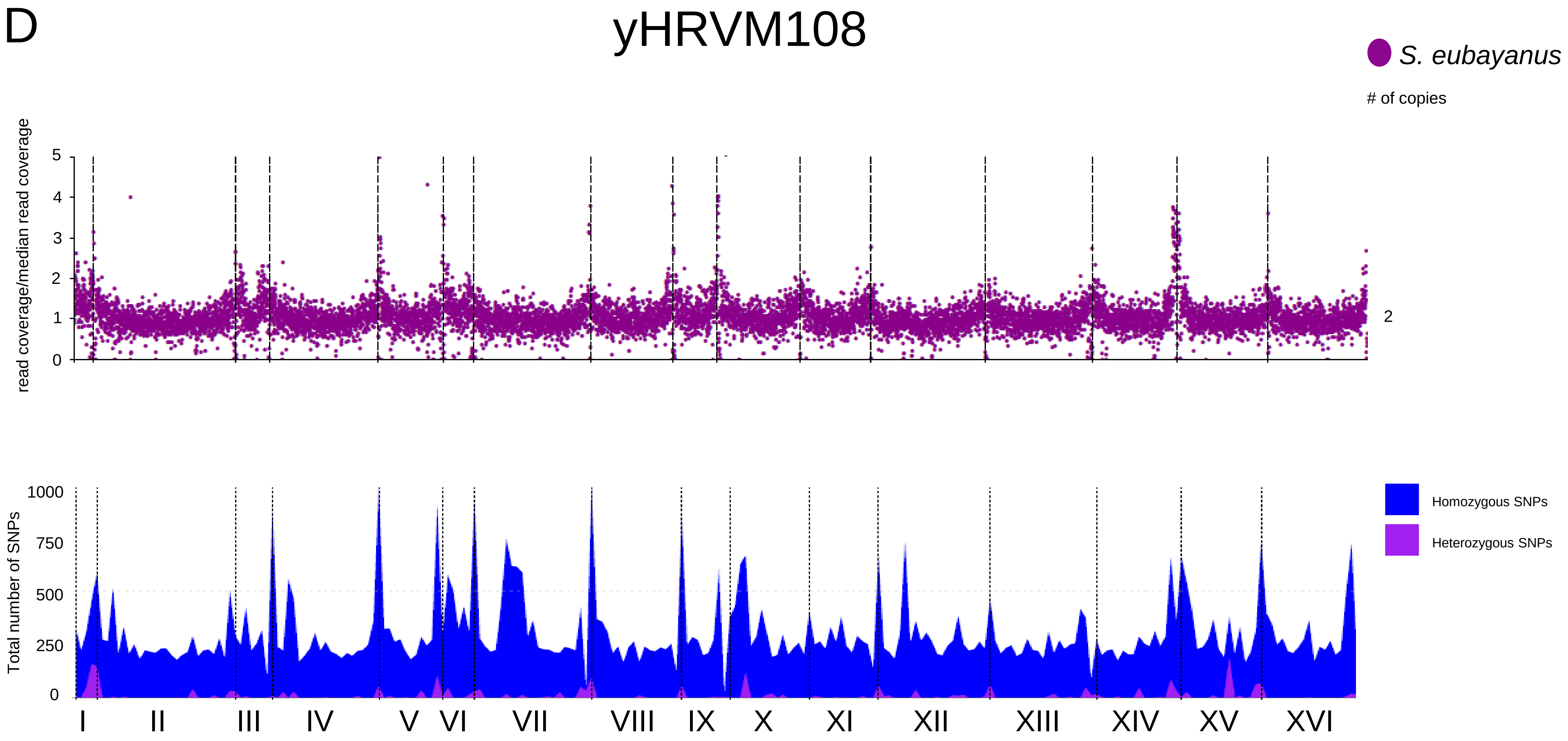
D
yHRVM108
S. eubayanus
# of copies
read coverage/median read coverage
0
1
2
3
4
5
2
Total number of SNPs
0
250
500
750
1000
Homozygous SNPs
Heterozygous SNPs
I
II
III
IV
V
VI
VII
VIII
IX
X
XI
XII
XIII
XIV
XV
XVI

E

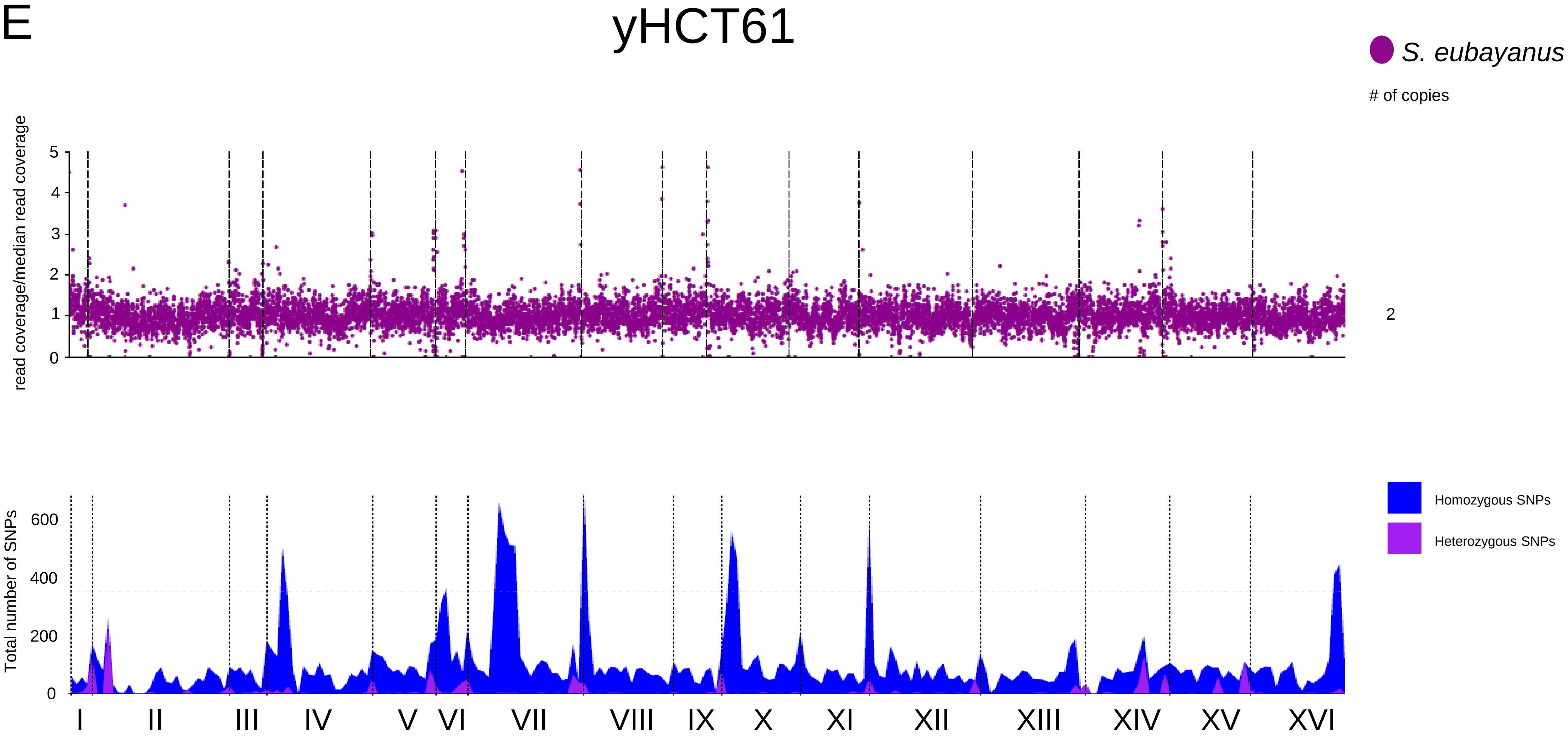

yHCT61
S. eubayanus
# of copies
read coverage/median read coverage
0
1
2
3
4
5
2
Total number of SNPs
0
200
400
600
Homozygous SNPs
Heterozygous SNPs
I
II
III
IV
V
VI
VII
VIII
IX
X
XI
XII
XIII
XIV
XV
XVI

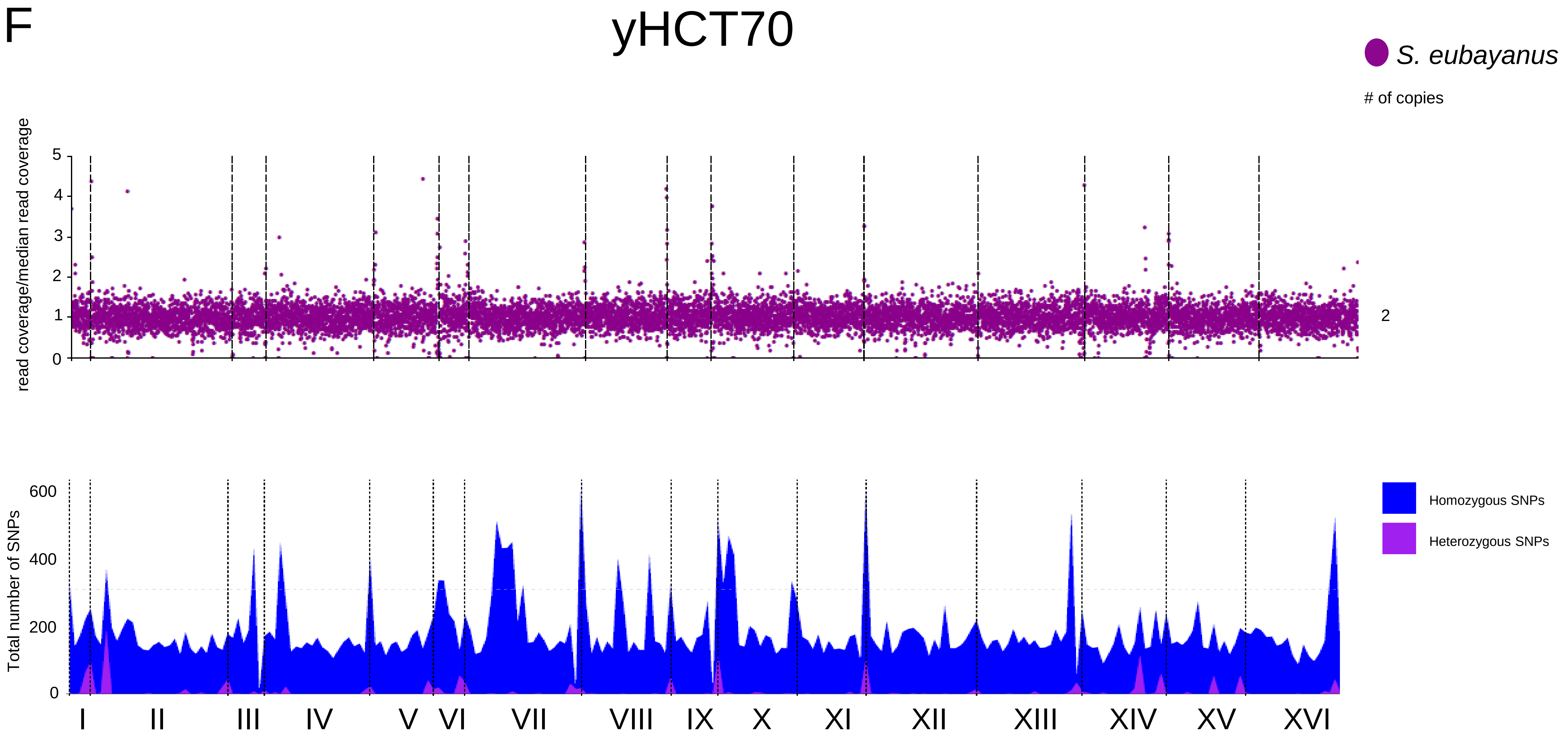

F
yHCT70
S. eubayanus
# of copies
read coverage/median read coverage
5
4
3
2
1
0
2
Total number of SNPs
600
400
200
0
Homozygous SNPs
Heterozygous SNPs
I
II
III
IV
V
VI
VII
VIII
IX
X
XI
XII
XIII
XIV
XV
XVI

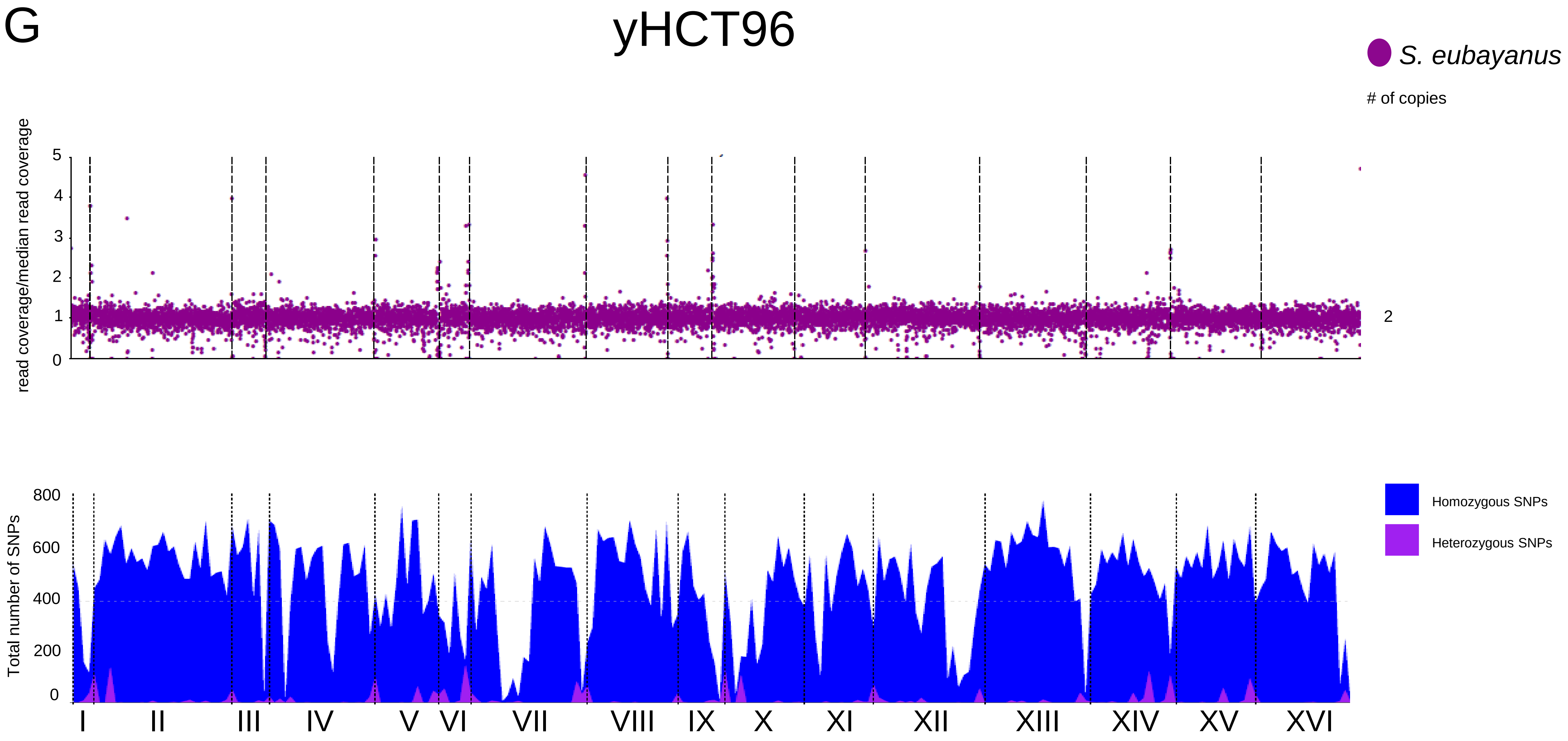
G
yHCT96
S. eubayanus
# of copies
read coverage/median read coverage
5
4
3
2
1
0
2
Total number of SNPs
800
600
400
200
0
Homozygous SNPs
Heterozygous SNPs
I
II
III
IV
V
VI
VII
VIII
IX
X
XI
XII
XIII
XIV
XV
XVI

H

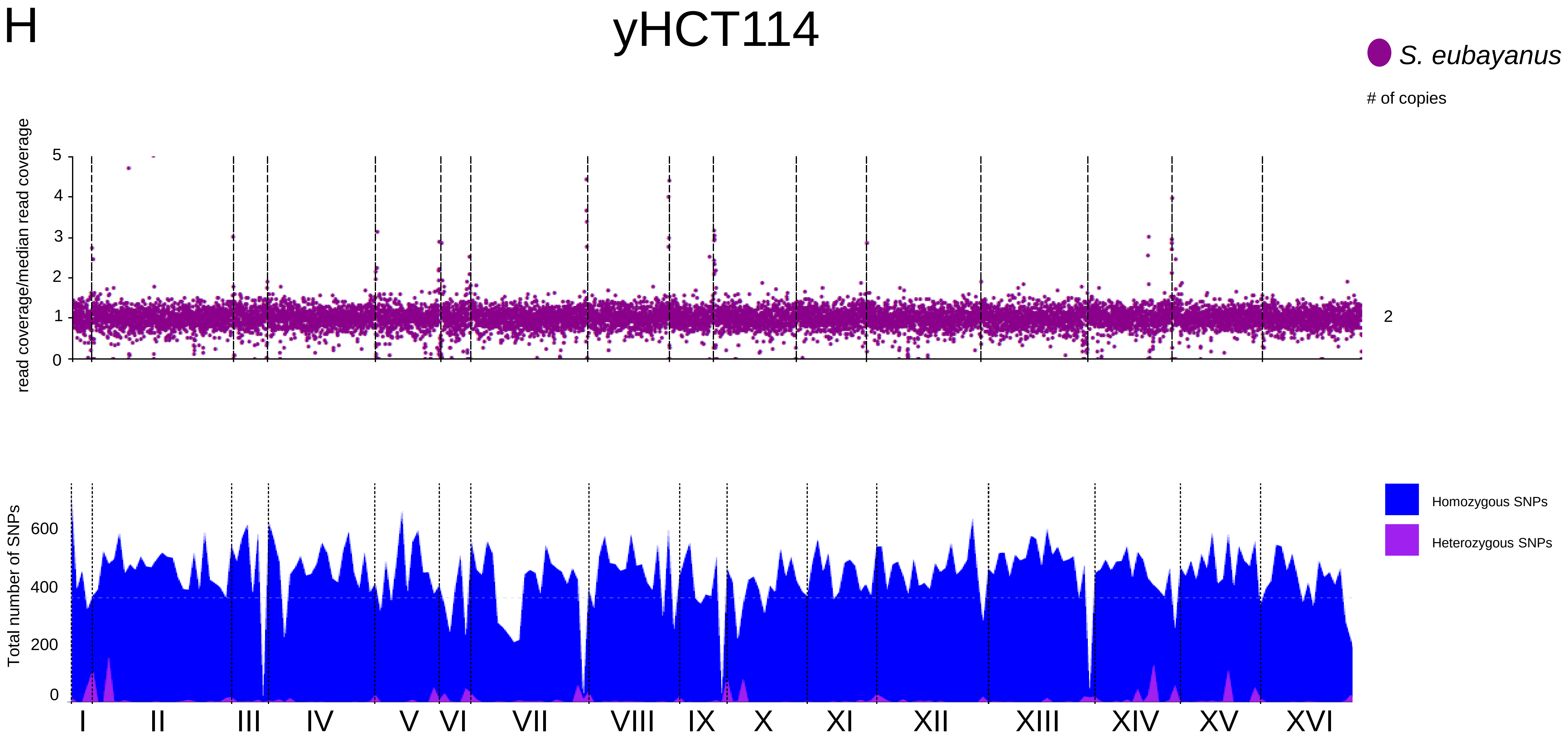
yHCT114
S. eubayanus
# of copies
read coverage/median read coverage
5
4
3
2
1
0
2
Total number of SNPs
600
400
200
0
Homozygous SNPs
Heterozygous SNPs
I
II
III
IV
V
VI
VII
VIII
IX
X
XI
XII
XIII
XIV
XV
XVI

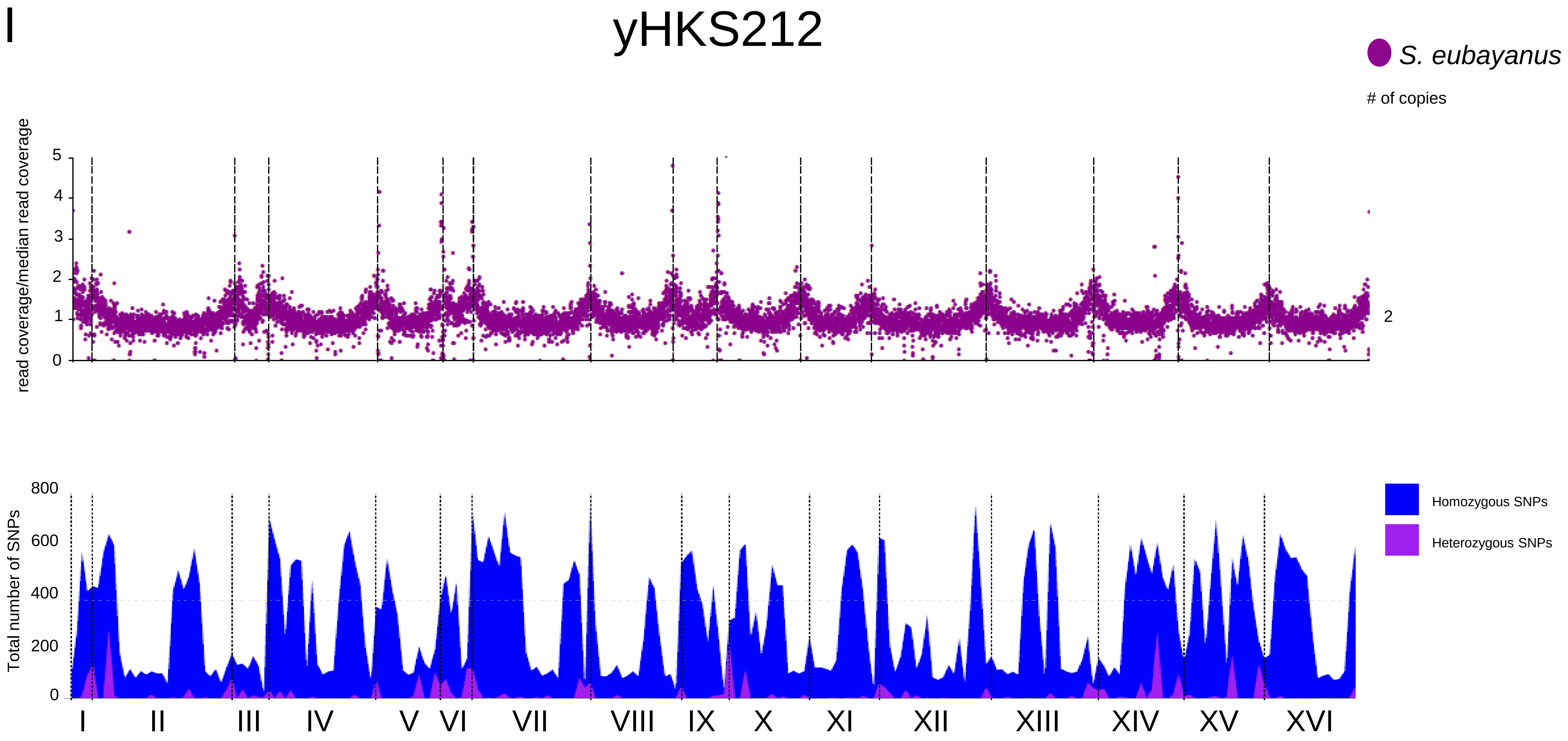
I
yHKS212
S. eubayanus
# of copies
read coverage/median read coverage
5
4
3
2
1
0
2
Total number of SNPs
800
600
400
200
0
Homozygous SNPs
Heterozygous SNPs
I
II
III
IV
V
VI
VII
VIII
IX
X
XI
XII
XIII
XIV
XV
XVI

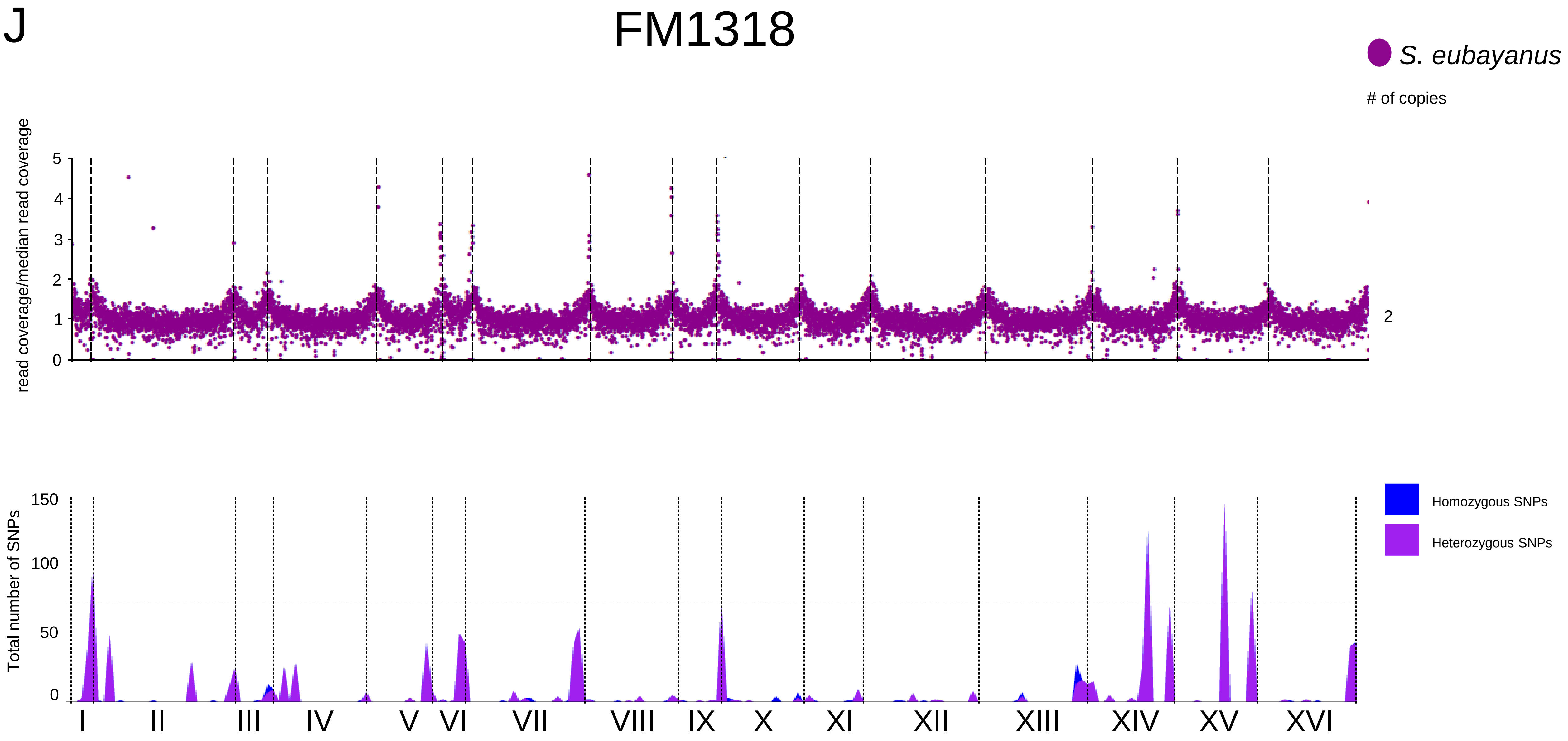
J
FM1318
S. eubayanus
# of copies
read coverage/median read coverage
5
4
3
2
1
0
2
Total number of SNPs
150
100
50
0
Homozygous SNPs
Heterozygous SNPs
I
II
III
IV
V
VI
VII
VIII
IX
X
XI
XII
XIII
XIV
XV
XVI
