# Supplementary material for: Complex Ancestries of Lager-Brewing Hybrids Were Shaped by Standing Variation in the Wild Yeast Saccharomyces eubayanus
Source: PLoS Genet. 2016 Jul 6;12(7):e1006155. doi: 10.1371/journal.pgen.1006155 (PMC4934787; doi:10.1371/journal.pgen.1006155)
Supplement: Supplementary file 11 [file pgen.1006155.s019.docx]

```
                                                    1111111111111111111111111222222222222222333333333333333333333333444444444444444444444444455
                 222333334444444555556666777777777889900002222222233356667779122457778899000123334444455567788890011111223445566677899990 0
              380451234523467891346567902345789672456782345679245002945835480506846370677338912359236014149025156790463568289079014912
CDFM21L.1     TCATTATCTATCTAACACGACGTAAGCGAGACAAACTACTTGATGTTACTAACCTCACGGGTTACCGCACCCGTCGCACGGAAAGTTTTATGGAGGGGCGAGTTTTATCTTCCCTAATGT
FM1318        ...................................T..T...............A.....................G...............A.......................G..
TTH25L.1      .TTAA.CTCCA.GG..GTAGTT.GCAGTGC.T..TTAG.C..C.A.C......T..T.A.ACC......TTTAGT.TG.....GA..A.C...GAAC..A..CGC.GA..G.T..C...C
TTH27L.1      .TTAA.CTCCA.GG..GTAGTT.GCAGTGC.T..TTAG.C..C.A.C......T..T.A.ACC......TTTAGT.TG.....GA..A.C...GAAC..A..CGC.GA..G.T..C...C
CBS7001       GTTA.G.....AA.TG.-A.TAG.CAGTGCTAGG.T....GAGGCCCGTCTGATA..T.CA..TATATGT....TCTGACCCGGACC.C.CACGA..AG.TCGGCC..TCGG.TC.C.AG

                                                                                     11111111111111111111111111111111111111111111111
              555555555555555555555555555666666666666666666667777777777778888888888999999000000000001111111111222222222223333333333444444
              000122233334445556667777899001111112333335667900122345667900123555504567812346677880234445669013447889903455677892345688
              589034746891672364570567507231567898167893054917058718143107576147851627332764709021435685693812147140214308136611987317
CDFM21L.1     TATCGCCAAAACCCTTCAAACGAGAATTAGAAGGATTCAATAAACGCCCCATTAGCATGTGTAATCCCATCTAGGTCCTTCTGAGCCACGGCTCTTTGCGCACGTATTCGCAAAATATGC
FM1318        ...................T.....................G....G.........................................................................
TTH25L.1      CGC...T.T.C.T.C.T.GT..TA.C.C.A.GA..AGAGGCCG..AG..G.C.T.T.C.CA..GAT....T..AC.AAC..C.CA...T.ATCTCC..T.T..A.....ATG..TCG.AT
TTH27L.1      CGC...T.T.C.T.C.T.GT..TA.C.C.A.GA..AGAGGCC.GT.GTT.G.CGA.GCAC.CGG..ATCC.CGCAC.T.ATCACATTG.AATC..CCC.ATGG.CGCCT.TGGGTC.CA.
CBS7001       ..CTAT.GTGTT.T.C.GGTTATATCC.TAG..AGAG..GCC.GT.GTT.G.CGA.GCAC.CGG..ATCC.CGCAC.T.ATCACATTG.AATC..CCC.ATGG.CGCCT.TGGGTC.CA.
```
